# Supplementary material for: An Interactive Lifestyle Medicine Curriculum for Third-Year Medical Students to Promote Student and Patient Wellness
Source: MedEdPORTAL. 2020 Sep 18;16:10972. doi: 10.15766/mep_2374-8265.10972 (PMC7499809; doi:10.15766/mep_2374-8265.10972)
Supplement: Supplementary file 1 — Introduction & Stress Management Presentation.pptxIntroduction & Stress Management Facilitator Guide.docxUnhealthy Thoughts Handout.pdfGood Things Worksheet.pdfNutrition Presentation.pptxNutrition Facilitator Guide.docxPhysical Activity Presentation.pptxPhysical Activity Facilitator Guide.docxPresession Evaluation.docxPostsession Evaluation.docxSession Evaluation.docx [file mep_2374-8265.10972-s001.zip › _Educational_Summary_Report_10972.pdf]

MedEdPORTAL® The AAMC Journal of Teaching and Learning Resources

Original Publication

Open Access

An Interactive Lifestyle Medicine Curriculum for Third-Year Medical Students to Promote Student and Patient Wellness

Jennifer Rockfeld, MD*, Jonathan Koppel, MD, Alexander Buell, Rebecca Zucconi, MD

*Corresponding author: jennifer.rockfeld@quinnipiac.edu

Abstract

Introduction: Although lifestyle intervention and behavior modification are effective in the prevention and treatment of chronic disease, few medical schools provide specific training in stress management, nutrition, or physical activity. While the prevalence of chronic disease rises, medical students and physicians lack sufficient knowledge and skills to promote their patients' as well as their own wellness across these domains. **Methods:** We developed three hour-long workshops delivered to third-year medical students. We employed interactive lectures, small-group discussions, and reflective activities to teach the pillars of lifestyle medicine. These sessions focused on knowledge and skills to advance lifestyle counseling and behavior modification interventions with patients and to promote student wellness. We assessed student satisfaction with each session as well as self-perceived knowledge, skills, and attitudes toward lifestyle medicine and behavior change before and after the curriculum. **Results:** Over 2 years, 183 students participated in the workshop series. The sessions received high ratings, with a mean of 4.2 on a 5-point Likert scale. Participating in the curriculum significantly enhanced students' understanding of the connection between lifestyle factors and the health of patients and improved their confidence about counseling for behavioral change. **Discussion:** Lifestyle medicine provides an evidence-based framework for teaching students about the impact of lifestyle modification on chronic disease. While receiving knowledge and skills to advance patient care in the domains of stress management, nutrition, and physical activity, students who completed this curriculum also had the opportunity to reflect on their own health promotion, which could mitigate professional burnout.

Keywords
Lifestyle Medicine, Healthy Lifestyle, Student Wellness, Nutrition, Nutritional Science, Physical Activity, Stress Management, Virtual Learning

Educational Objectives

By the end of this curriculum, learners will be able to:

1. Describe the pillars of lifestyle medicine.
2. Examine the contribution of lifestyle and behavioral factors to health and wellness.
3. Counsel patients about positive behavioral changes in the areas of stress management, nutrition, and physical activity.
4. Identify opportunities to support their own health and wellness.

Citation:
Rockfeld J, Koppel J, Buell A, Zucconi R. An interactive lifestyle medicine curriculum for third-year medical students to promote student and patient wellness. *MedEdPORTAL*. 2020;16:10972.
https://doi.org/10.15766/mep_2374-8265.10972

Introduction

Chronic diseases, especially heart disease, cancer, and type 2 diabetes, are the leading causes of death and disability in the United States and place a significant economic burden on our health care system.[1] Public health agencies and professional societies routinely include lifestyle intervention and behavior modification in their guidelines for the prevention and treatment of chronic diseases.[2-5] Despite these recommendations, however, few medical schools are providing students with skills to effectively counsel patients about behavior change in the domains of stress management, nutrition, and physical activity.[6]

The American College of Lifestyle Medicine defines lifestyle medicine as

> the use of evidence-based lifestyle therapeutic approaches, such as a whole food, plant-predominant dietary lifestyle, regular physical activity, restorative sleep, stress management, avoidance of risky substances

Copyright © 2020 Rockfeld et al. This is an open-access publication distributed under the terms of the Creative Commons Attribution license.

and positive social connection as a primary therapeutic modality for treatment and reversal of chronic disease.[7]

Practitioners of lifestyle medicine assist patients and families in adopting and sustaining behaviors that will improve their health and quality of life. Following the recommendations of professional stakeholders to include lifestyle medicine education in undergraduate medical education curricula, our Lifestyle Medicine and Medical Student Wellness workshops are aimed at third-year medical students and designed to enhance their attitude, clinical skills, and self-efficacy in three of these domains: stress management, nutrition, and physical activity.[8,9]

Concurrent with the rising prevalence of chronic diseases in their patients, medical students themselves are experiencing high levels of stress, burnout, and a lack of self-efficacy in their own health care promotion.[10]

Educating medical students about lifestyle medicine early in their training and providing resources to support their wellness may mitigate burnout and promote resiliency.[11] Furthermore, there is evidence of a positive association between the attitudes and behaviors of physicians and the efficacy of preventive health interventions with their patients.[12]

Although an expanding array of resources exists to train graduates and practicing physicians in the competencies of lifestyle medicine, we found few published resources providing comprehensive knowledge and skills aimed specifically at medical students. Several *MedEdPORTAL* publications deal with obesity counseling and behavioral change curricula, and one offers a wellness program for undergraduate medical students, but none specifically focus on the discipline of lifestyle medicine that simultaneously emphasizes patient and personal wellness.[13-17]

Recognizing these curricular gaps at our own institution and appreciating the unique stressors and obstacles our students face in trying to make positive lifestyle choices, we developed and delivered a series of interactive workshops with the goal of teaching students about the lifestyle and behavioral factors that affect the health of both the patients they care for and themselves. We used the combined instructional modalities of didactic presentations, small-group discussions, and individual reflective practices to deliver curricular content and engage students. Our novel approach encouraged students to reflect on their own goals, behaviors, and opportunities for change, an educational priority for us. Each of the three sessions in this series could be delivered individually to emphasize objectives within a specific domain (stress management, nutrition, physical activity). Ideally, however, students would develop a more robust appreciation for the impact of lifestyle factors on disease and wellness through the sequential experience of all three sessions.

Methods

At the Frank H. Netter MD School of Medicine at Quinnipiac University, third-year medical students return to campus for 1 week four times a year to attend mandatory sessions on a range of topics, including ethics and professionalism, narrative medicine, and student advising. We piloted our Lifestyle Medicine and Medical Student Wellness series during these weeks in the 2017-2018 academic year and repeated it in 2018-2019. Students were not expected to prepare for these sessions; however, they had been introduced to the concept of lifestyle medicine, as well as behavioral change theory, during their clinical skills and behavioral and social sciences courses in the preclinical curriculum.

The content was developed and delivered by two physicians (JR and RZ). Each session focused on one of the pillars of lifestyle medicine and incorporated the most current evidence-based literature. The workshops each contained the same components: discussion of the relevance of the topic to wellness and the prevention of chronic disease, reflection on the students' personal experiences, and application of behavioral change theory to patient care. Each session listed below had the following associated materials:

- Lifestyle Medicine and Student Wellness: Introduction and Stress Management (Appendix A: PowerPoint, Appendix B: facilitator guide, Appendix C: handout,[18] Appendix D: handout).
- Lifestyle Medicine and Student Wellness: Nutrition (Appendix E: PowerPoint, Appendix F: facilitator guide).
- Lifestyle Medicine and Student Wellness: Physical Activity (Appendix G: PowerPoint, Appendix H: facilitator guide).

There were approximately 90 students present for each session, which was held in a large room with tables of eight to 10 students each. Each 1-hour workshop required AV equipment, flip charts or whiteboards, and blank paper for reflective activities. We utilized a combination of PowerPoint presentations to deliver content and breakout activities to engage the students. Most of the sessions began with a brief introduction to the topic and learning objectives followed by a reflective exercise with a table discussion (e.g., draw out your dinner plate, write three sources of stress). The facilitators then provided background information on the topic in lecture format. For several of the workshops,

Copyright © 2020 Rockfeld et al. This is an open-access publication distributed under the terms of the Creative Commons Attribution license.

we had students share their strategies to develop or maintain a healthy lifestyle behavior. In the nutrition workshop, the students shared barriers to healthy eating that we wrote on each flip chart. Subsequently, students who had overcome that barrier wrote out their tips, and a reporter read off their collective wisdom to the entire group. At the end of the workshop, the facilitators pulled the strategies together in a document that we distributed to the class. Whenever possible, we practiced techniques as a group; for example, we performed a guided deep breathing activity in the stress management workshop and went for a walk at the end of the physical activity session.

We developed a survey to assess each individual session as well as an overall pre/post evaluation of students' self-perceived knowledge, skills, and attitudes about lifestyle medicine and behavioral counseling skills (Appendices I and J). Students indicated on a 5-point Likert scale (1 = *strongly disagree,* 5 = *strongly agree*) whether they felt lifestyle factors were an important contributor to health and wellness, whether they understood the relationship between lifestyle factors and wellness, and whether they felt confident in counseling about positive behavior changes. The evaluation specifically separated how students felt about patient care from their views on their own health and wellness. After each workshop, the students rated the session on 5-point Likert scale (1 = *poor*, 5 = *excellent*), with narrative comments about what they liked best and what they would change (Appendix K). We utilized a paper-based format to get the highest possible response rate.

Results

Over the course of 2 years, 183 students participated in the Lifestyle Medicine and Medical Student Wellness curriculum. A total of 161 students (88%) completed the presession evaluation (Appendix I) prior to the first session, and 108 students (59%) completed the postsession evaluation (Appendix J) at the end of the third session. Both before and after the curriculum, most students felt that lifestyle factors were important to their patients' and their own health and wellness. After the intervention, students were significantly more likely to understand the relationship between lifestyle factors and their patients' wellness and had increased confidence in their ability to counsel patients about positive behavioral changes. In terms of students' own health, there was a nonsignificant improvement in their confidence about making their own positive behavioral changes (Table 1).

The sessions received high ratings from the students on the session evaluation (Appendix K), with a mean of 4.2 on a 5-point Likert scale (1 = *poor,* 5 = *excellent*; Table 2).

Table 1. Ratings of Lifestyle Medicine and Student Wellness Curriculum Pre-/Postevaluations[a]

Item	Pre (N = 161)		Post (N = 108)		
	M	*SD*	*M*	*SD*	*p*
Lifestyle factors important to patient's wellness.	4.8	0.4	4.9	0.4	.212
Lifestyle factors important to own wellness.	4.8	0.4	4.8	0.4	.736
Relationship between lifestyle and wellness of patient.	4.5	0.7	4.7	0.5	.003
Relationship between lifestyle factors and own wellness.	4.6	0.6	4.7	0.6	.166
Confident counsel patients about behavioral changes.	3.8	0.8	4.3	0.6	<.001
Confident make own behavioral changes.	3.8	0.8	3.9	0.9	.372

[a]Rated on a 5-point Likert scale (1 = *strongly disagree,* 5 = *strongly agree*).

Students were asked open-ended questions regarding what they liked best about each session and what they would change. Several comments reflected on the interactive nature of the sessions:

- "I liked that the session was interactive and had a positive message for all of us and our patients."
- "I liked addressing barriers and coming up with solutions as a group."

Many commented on the curriculum's timeliness and value to their own lives. They appreciated the concrete suggestions provided for each topic and how these could be applied to both their patients and themselves:

- "Useful student input and real solutions to problems, demonstration that other people struggle with the same things."
- "I like learning about concrete steps I can take to reduce my stress."
- "Important to our lives, helpful info to tell patients."

Students noted that wellness was a topic frequently overlooked in the formal curriculum:

- "It was a fun and important topic that is often taken for granted and it was nice to have it addressed."

Table 2. Ratings of Each Session on a 5-Point Likert Scale (1 = *poor,* 5 = *excellent*)

Session	Topic	*M*	*SD*
1	Stress management	4.18	0.73
2	Nutrition	4.15	0.75
3	Physical activity	4.19	0.83

Copyright © 2020 Rockfeld et al. This is an open-access publication distributed under the terms of the Creative Commons Attribution license.

- “Thank you for taking the time to share this with us and lead us through this exercise. Thank you for recognizing the importance of medical student self-care!”

The biggest consistent criticism of the sessions was that the students wanted the sessions to be longer to allow more time for discussions with classmates and practicing new skills. Students also critiqued the lack of broader systemic changes to support their personal wellness:

- “Tactics are good but acknowledge the two-faced elements of the system we are in. There’s a lot of pretending it doesn’t exist.”
- “It would be extremely helpful to have a larger gym on campus to take this initiative seriously.”
- “I know how to be well, but I don’t have time to be well. Until that factor is addressed, ‘wellness’ won’t get better.”

Discussion

Lifestyle medicine provides an evidence-based framework for teaching students about the impact of lifestyle modification on the treatment and prevention of chronic disease. Overall, this curriculum was very well received by students. They enjoyed the interactive nature of the sessions and collaborating with their peers, especially during a time of training when they are usually separated at diverse sites. Students believed that lifestyle factors were important to their own and their patients’ health prior to undergoing the curriculum, and those beliefs remained stable at the end. Addressing knowledge and skills in a formal curriculum improved both students’ understanding of the relationship between lifestyle factors and patients’ health and students’ self-perceived behavioral counseling skills.

Not surprisingly, students had less confidence in their ability to make positive behavioral changes for themselves. Most of the critical feedback on the sessions focused on systemic issues out of students’ control that impeded their ability to implement healthy choices. We attempt to promote these values at our institution but are aware of the challenges associated with maintaining wellness during medical training. Students did appreciate the focus on their wellness and the positive message conveyed during these sessions. We have brought some of their suggestions back to our administrative leadership, and students have also taken the initiative to build upon the concepts learned. For example, the current fourth-years are in the process of creating a healthy, low-budget electronic cookbook for our community, and they share a weekly wellness tip in our Student Affairs Newsletter.

We learned several lessons when implementing this curriculum. Given that curricular time is difficult to obtain, we capitalized on an opportunity to fill a scheduling gap that occurred between clerkship blocks during third year. Perhaps not surprisingly, we found that delivering this content when students did not have competing clinical responsibilities allowed for greater engagement. This timing gave students space to reflect and provided an opportunity for them to commiserate in a productive manner. Facilitators need to be adept at redirecting students to be constructive, rather than critical, when reflecting on their own wellness within the limitations of our current medical system.

One major limitation of the curriculum was its predominantly lecture-based format offered in one large classroom. We found that having a space with several round tables fit small-group discussions better than a lecture hall setting did. The curriculum was also distributed over the course of the year, rather than condensed into a block of time, which was a challenge as students may have perceived the sessions as stand-alone events.

Each session contained at least one component to engage the students in the process of improving their own health, and we attempted to make the discussions as interactive as possible. Preferably, however, these sessions would have utilized more active learning principles and focused on skills training. An alternative flipped classroom approach could incorporate an assignment prior to the session to deliver knowledge and prompt students to reflect on their own lifestyle choices. In this model, more classroom time could be dedicated to group discussion and skills practice with role-playing activities.

At this time, we have evaluated only self-perceived knowledge, skills, and attitudes, rather than an assessment of students’ knowledge and skills in either simulated or actual practice. Given the large number of clinical settings and diverse student schedules, it would have been difficult to assess whether students applied the concepts to subsequent rotations while controlling for other variables. In the future, we are considering an objective structured clinical examination to look at students’ behavioral counseling skills with standardized patients in a controlled setting.

We continue to teach these sessions at our institution and are exploring future lifestyle medicine electives in areas such as culinary medicine. Part of the strategic plan for our school includes a focus on student wellness, which will hopefully provide institutional support for the tenets we promote in our sessions. The best measure of our success will be whether these students

Copyright © 2020 Rockfeld et al. This is an open-access publication distributed under the terms of the Creative Commons Attribution license.

continue to value the impact of lifestyle choices on health and utilize these skills in their residencies and future careers.

Appendices

A. Introduction & Stress Management Presentation.pptx
B. Introduction & Stress Management Facilitator Guide.docx
C. Unhealthy Thoughts Handout.pdf
D. Good Things Worksheet.pdf
E. Nutrition Presentation.pptx
F. Nutrition Facilitator Guide.docx
G. Physical Activity Presentation.pptx
H. Physical Activity Facilitator Guide.docx
I. Presession Evaluation.docx
J. Postsession Evaluation.docx
K. Session Evaluation.docx

All appendices are peer reviewed as integral parts of the Original Publication.

Jennifer Rockfeld, MD: Assistant Professor, Department of Medical Sciences, Frank H. Netter MD School of Medicine at Quinnipiac University; Assistant Course Director, Clinical Arts and Sciences Course, Frank H. Netter MD School of Medicine at Quinnipiac University

Jonathan Koppel, MD: Intern, Primary Care Residency Program, Icahn School of Medicine at Mount Sinai

Alexander Buell: Fourth-Year Medical Student, Frank H. Netter MD School of Medicine at Quinnipiac University

Rebecca Zucconi, MD: Assistant Professor, Department of Medical Sciences, Frank H. Netter MD School of Medicine at Quinnipiac University; Course Director, Foundations of Medicine Course, Frank H. Netter MD School of Medicine at Quinnipiac University

Disclosures
None to report.

Funding/Support
None to report.

Ethical Approval:
Reported as not applicable.

References

1. About chronic diseases. Centers for Disease Control and Prevention: National Center for Chronic Disease Prevention and Health Promotion. Accessed August 10, 2019. https://www.cdc.gov/chronicdisease/about/index.htm
2. World Health Organization. *Global Strategy on Diet, Physical Activity and Health*. World Health Organization; 2004. Accessed August 10, 2019. https://www.who.int/dietphysicalactivity/strategy/eb11344/strategy_english_web.pdf
3. Eckel RH, Jakicic JM, Ard JD, et al. 2013 AHA/ACC guideline on lifestyle management to reduce cardiovascular risk: a report of the American College of Cardiology/American Heart Association Task Force on Practice Guidelines. *J Am Coll Cardiol*. 2014;63(25)(pt B):2960-2984. https://doi.org/10.1016/j.jacc.2013.11.003
4. American Diabetes Association. 5. Lifestyle management: *Standards of Medical Care in Diabetes—2019. Diabetes Care.* 2019;42(suppl 1):S46-S60. https://doi.org/10.2337/dc19-S005
5. Kushi LH, Doyle C, McCullough M, et al; American Cancer Society 2010 Nutrition and Physical Activity Guidelines Advisory Committee. American Cancer Society guidelines on nutrition and physical activity for cancer prevention. *CA Cancer J Clin*. 2012;62(1):30-67. https://doi.org/10.3322/caac.20140
6. Phillips E, Pojednic R, Polak R, Bush J, Trilk J. Including lifestyle medicine in undergraduate medical curricula. *Med Educ Online*. 2015;20(1):26150. https://doi.org/10.3402/meo.v20.26150
7. Mission/vision. American College of Lifestyle Medicine. *Accessed August* 10, 2019. https://www.lifestylemedicine.org/ACLM/About/Mission_Vision/ACLM/About/Mission_Vision.aspx?hkey=0c26bcd1-f424-416a-9055-2e3af80777f6
8. Trilk J, Nelson L, Briggs A, Muscato D. Including lifestyle medicine in medical education: rationale for American College of Preventive Medicine/American Medical Association resolution 959. *Am J Prev Med*. 2019;56(5):e169-e175. https://doi.org/10.1016/j.amepre.2018.10.034
9. Lifestyle Medicine Education Collaborative. Accessed August 13, 2019. https://lifestylemedicineeducation.org/
10. Dyrbye LN, West CP, Satele D, et al. Burnout among U.S. medical students, residents, and early career physicians relative to the general U.S. population. *Acad Med*. 2014;89(3):443-451. https://doi.org/10.1097/ACM.0000000000000134
11. Sklar DP. Fostering student, resident, and faculty wellness to produce healthy doctors and a healthy population. *Acad Med*. 2016;91(9):1185-1188. https://doi.org/10.1097/ACM.0000000000001298
12. Frank E, Dresner Y, Shani M, Vinker S. The association between physicians' and patients' preventive health practices. *CMAJ*. 2013;185(8):649-653. https://doi.org/10.1503/cmaj.121028
13. Craven K, Schmidt S, Kolasa K. Obesity counseling experience for third year medical students. *MedEdPORTAL*. 2014;10:9872. https://doi.org/10.15766/mep_2374-8265.9872
14. Pasarica M, Harris DM, Simms-Cendan J, Gorman AL. Collaborative learning activity utilizing evidence-based medicine

Copyright © 2020 Rockfeld et al. This is an open-access publication distributed under the terms of the Creative Commons Attribution license.

to improve medical student learning of the lifestyle management of obesity. *MedEdPORTAL*. 2016;12:10426. https://doi.org/10.15766/mep_2374-8265.10426

15. Pasarica M, Topping D. An evidence-based approach to teaching obesity management to medical students. *MedEdPORTAL*. 2017; 13:10662. https://doi.org/10.15766/mep_2374-8265.10662
16. Kolasa K, Craven K, Byrd J, Merricks P. Helping your patient change: a patient-centered behavioral counseling presentation for second-year medical students. *MedEdPORTAL*. 2015;11: 10166. https://doi.org/10.15766/mep_2374-8265.10166
17. Zackoff M, Sastre E, Rodgers S. Vanderbilt Wellness Program: model and implementation guide. *MedEdPORTAL*. 2012;8:9111. https://doi.org/10.15766/mep_2374-8265.9111
18. A dozen posters to combat stress. Center for Early Childhood Mental Health Consultation. *Accessed November* 25, 2019. https://www.ecmhc.org/documents/CECMHC_GrafittiPosters.pdf

Received: October 10, 2019
Accepted: March 16, 2020
Published: September 18, 2020

Copyright © 2020 Rockfeld et al. This is an open-access publication distributed under the terms of the Creative Commons Attribution license.
