# Supplementary material for: An Interactive Lifestyle Medicine Curriculum for Third-Year Medical Students to Promote Student and Patient Wellness
Source: MedEdPORTAL. 2020 Sep 18;16:10972. doi: 10.15766/mep_2374-8265.10972 (PMC7499809; doi:10.15766/mep_2374-8265.10972)
Supplement: Supplementary file 1 — Introduction & Stress Management Presentation.pptxIntroduction & Stress Management Facilitator Guide.docxUnhealthy Thoughts Handout.pdfGood Things Worksheet.pdfNutrition Presentation.pptxNutrition Facilitator Guide.docxPhysical Activity Presentation.pptxPhysical Activity Facilitator Guide.docxPresession Evaluation.docxPostsession Evaluation.docxSession Evaluation.docx [file mep_2374-8265.10972-s001.zip › A. Introduction & Stress Management Presentation.pptx]

## Slide 1
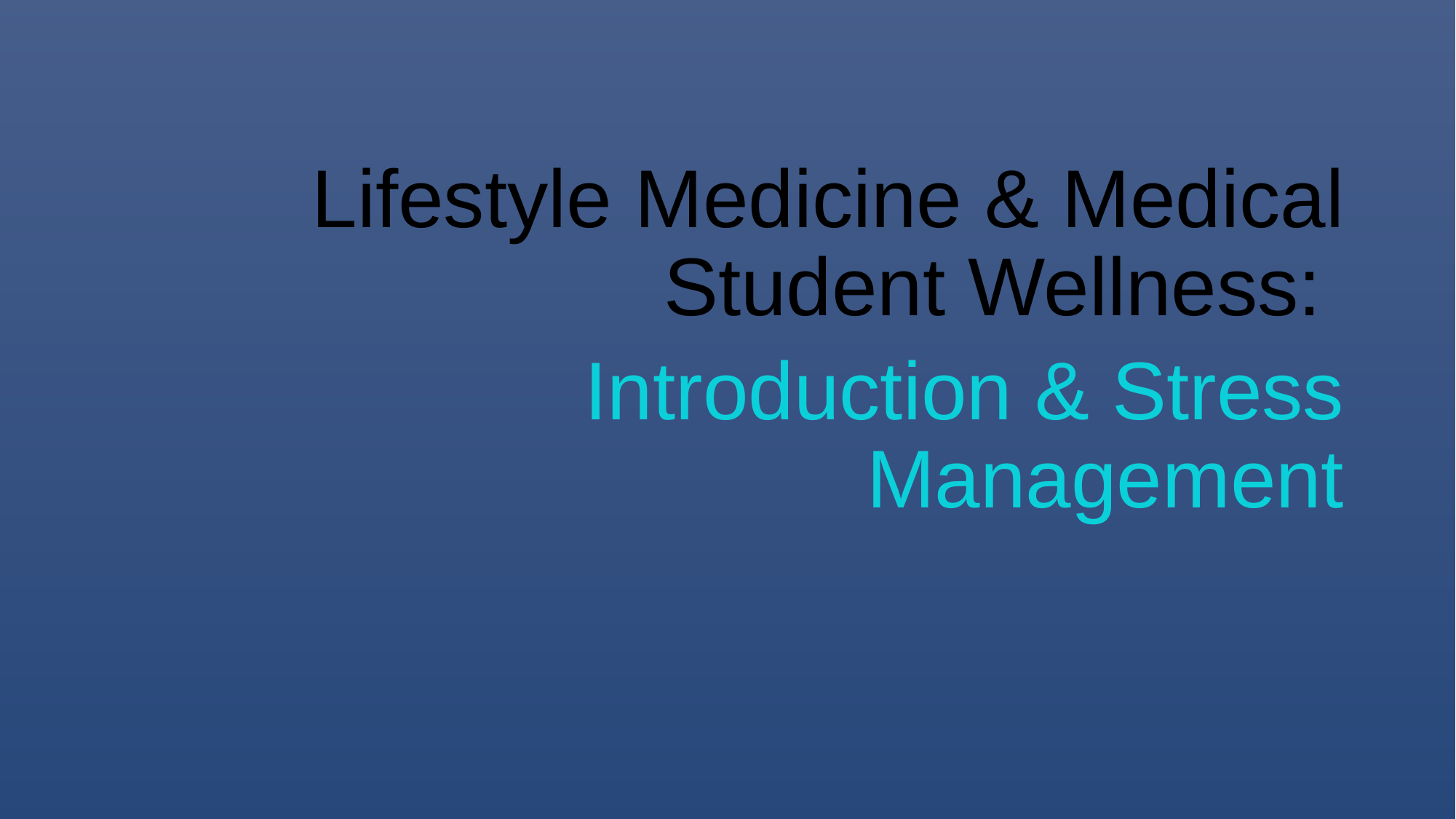

Lifestyle Medicine & Medical Student Wellness:
Introduction & Stress Management

## Slide 2
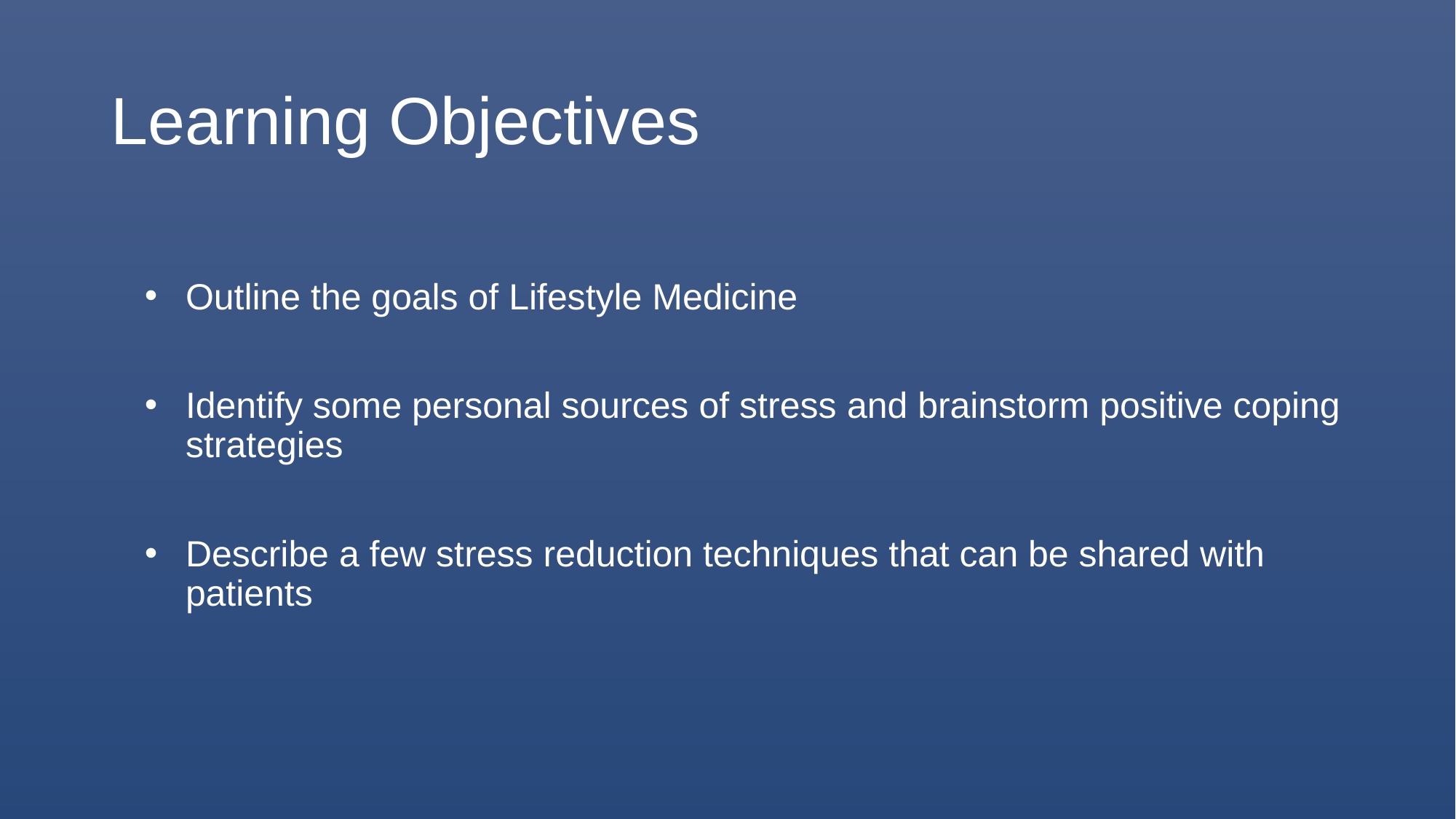

# Learning Objectives
Outline the goals of Lifestyle Medicine
Identify some personal sources of stress and brainstorm positive coping strategies
Describe a few stress reduction techniques that can be shared with patients

## Slide 3
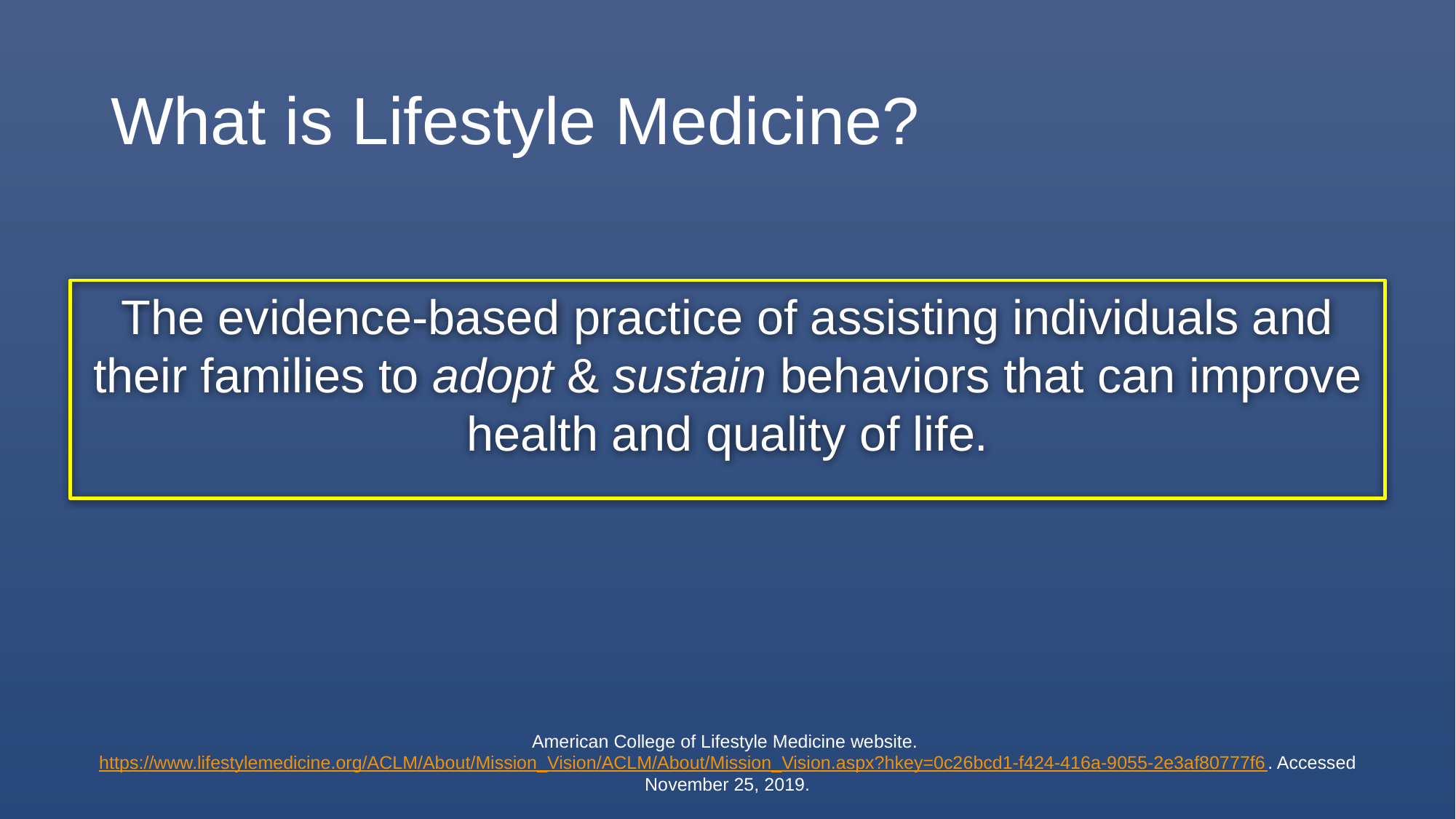

# What is Lifestyle Medicine?
The evidence-based practice of assisting individuals and their families to adopt & sustain behaviors that can improve health and quality of life.
American College of Lifestyle Medicine website. https://www.lifestylemedicine.org/ACLM/About/Mission_Vision/ACLM/About/Mission_Vision.aspx?hkey=0c26bcd1-f424-416a-9055-2e3af80777f6. Accessed November 25, 2019.

## Slide 4
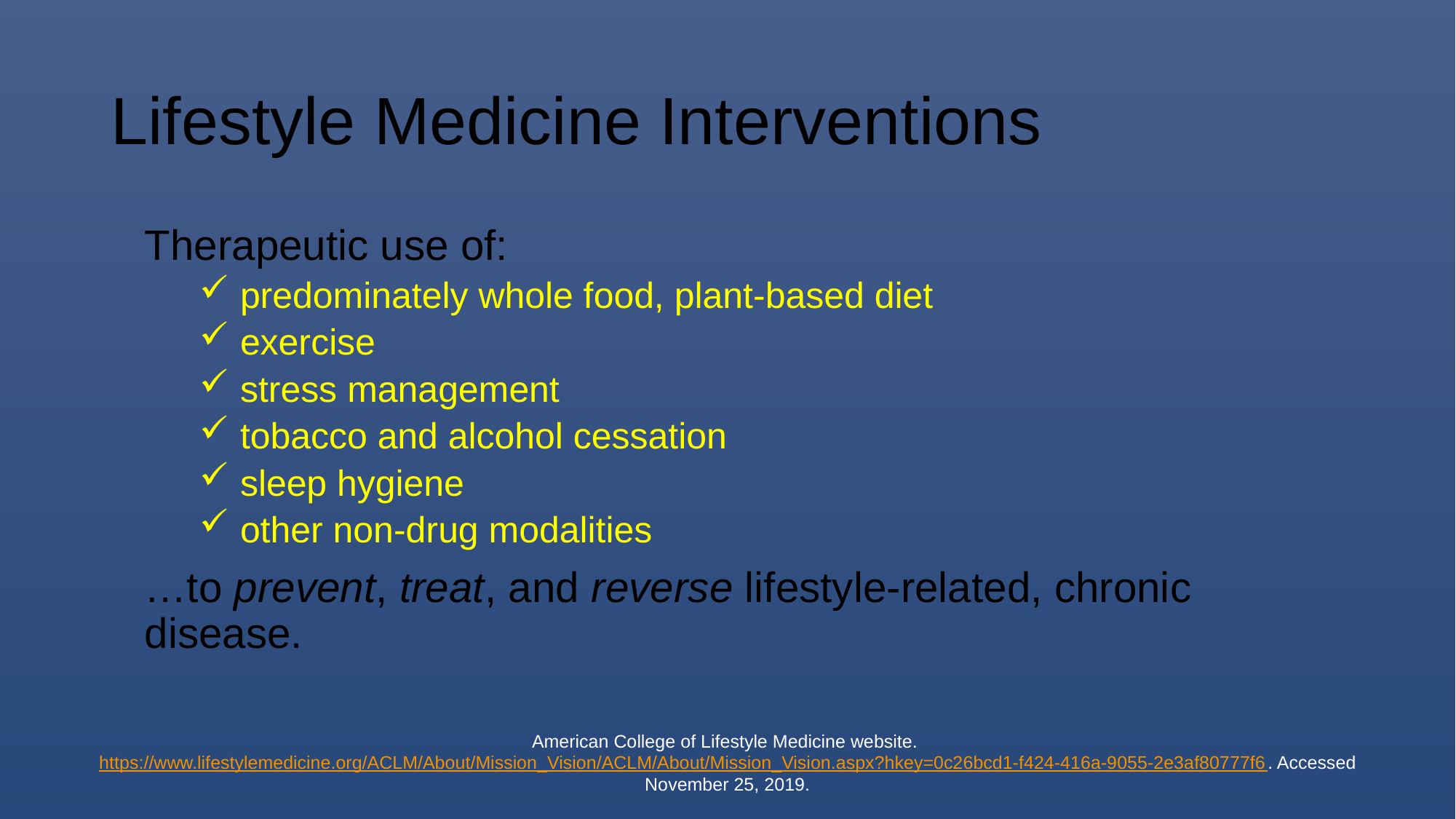

# Lifestyle Medicine Interventions
Therapeutic use of:
predominately whole food, plant-based diet
exercise
stress management
tobacco and alcohol cessation
sleep hygiene
other non-drug modalities
…to prevent, treat, and reverse lifestyle-related, chronic disease.
American College of Lifestyle Medicine website. https://www.lifestylemedicine.org/ACLM/About/Mission_Vision/ACLM/About/Mission_Vision.aspx?hkey=0c26bcd1-f424-416a-9055-2e3af80777f6. Accessed November 25, 2019.

## Slide 5
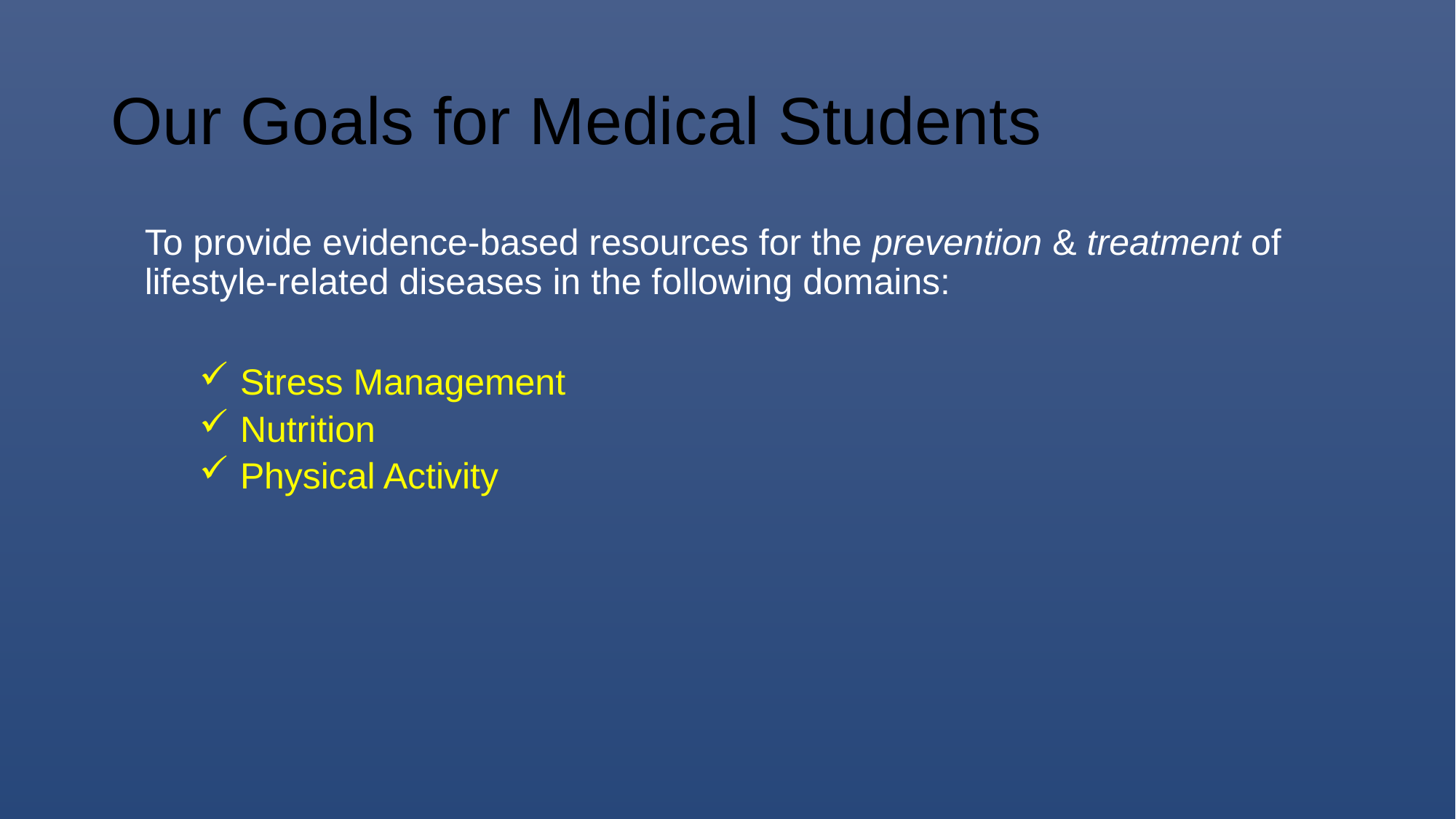

# Our Goals for Medical Students
To provide evidence-based resources for the prevention & treatment of lifestyle-related diseases in the following domains:
Stress Management
Nutrition
Physical Activity

## Slide 6
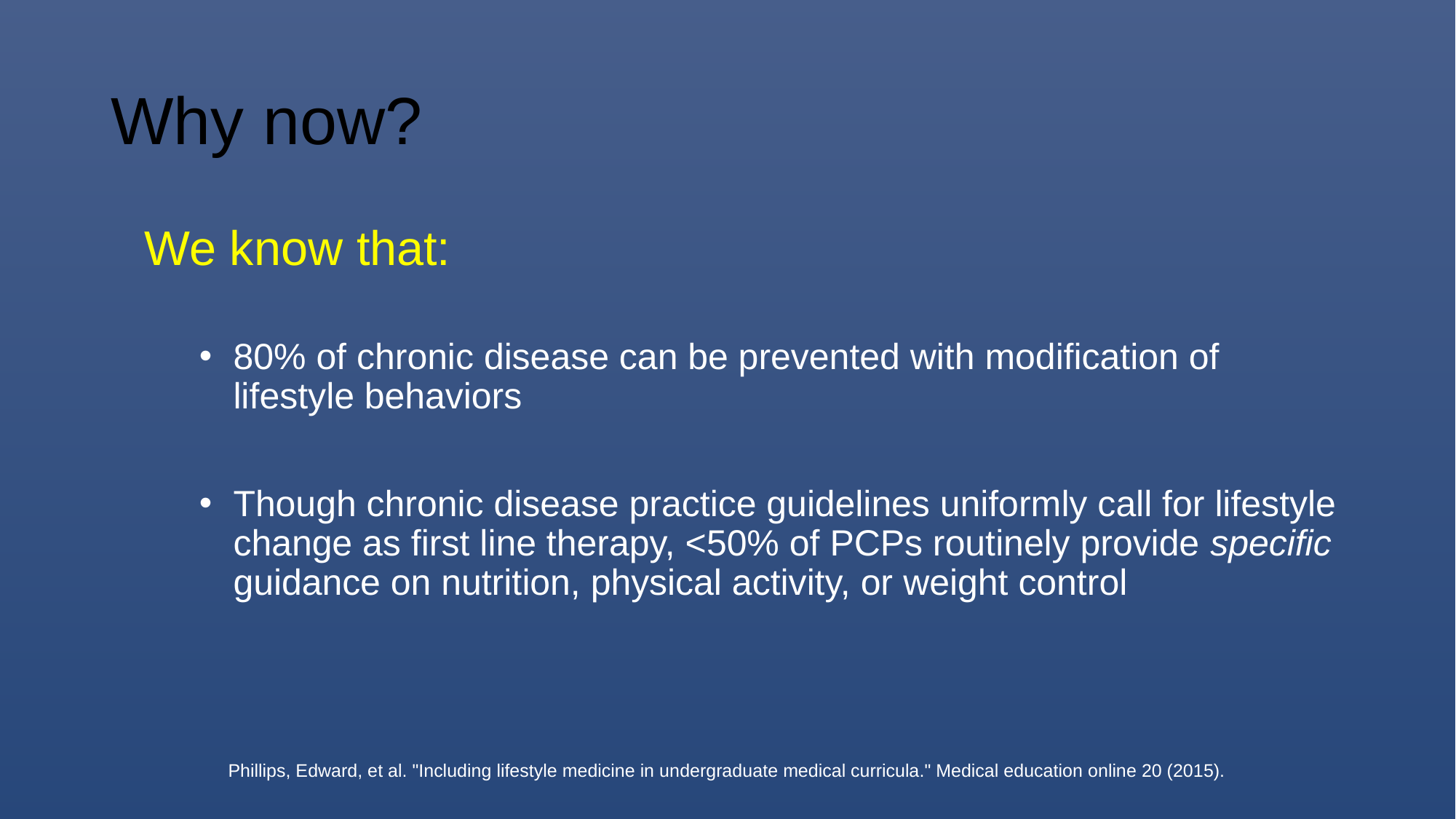

# Why now?
We know that:
80% of chronic disease can be prevented with modification of lifestyle behaviors
Though chronic disease practice guidelines uniformly call for lifestyle change as first line therapy, <50% of PCPs routinely provide specific guidance on nutrition, physical activity, or weight control
Phillips, Edward, et al. "Including lifestyle medicine in undergraduate medical curricula." Medical education online 20 (2015).

## Slide 7
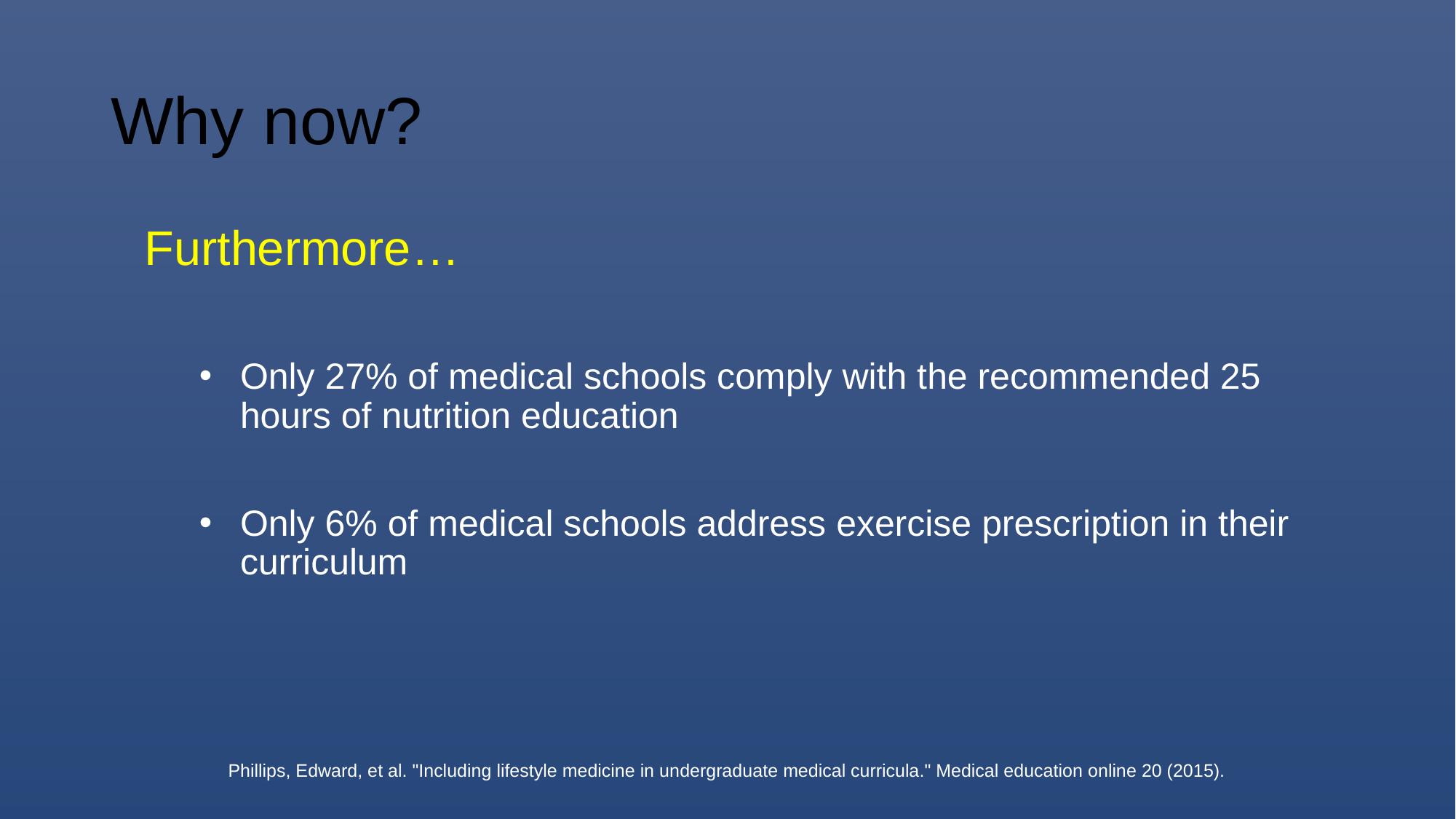

# Why now?
Furthermore…
Only 27% of medical schools comply with the recommended 25 hours of nutrition education
Only 6% of medical schools address exercise prescription in their curriculum
Phillips, Edward, et al. "Including lifestyle medicine in undergraduate medical curricula." Medical education online 20 (2015).

## Slide 8
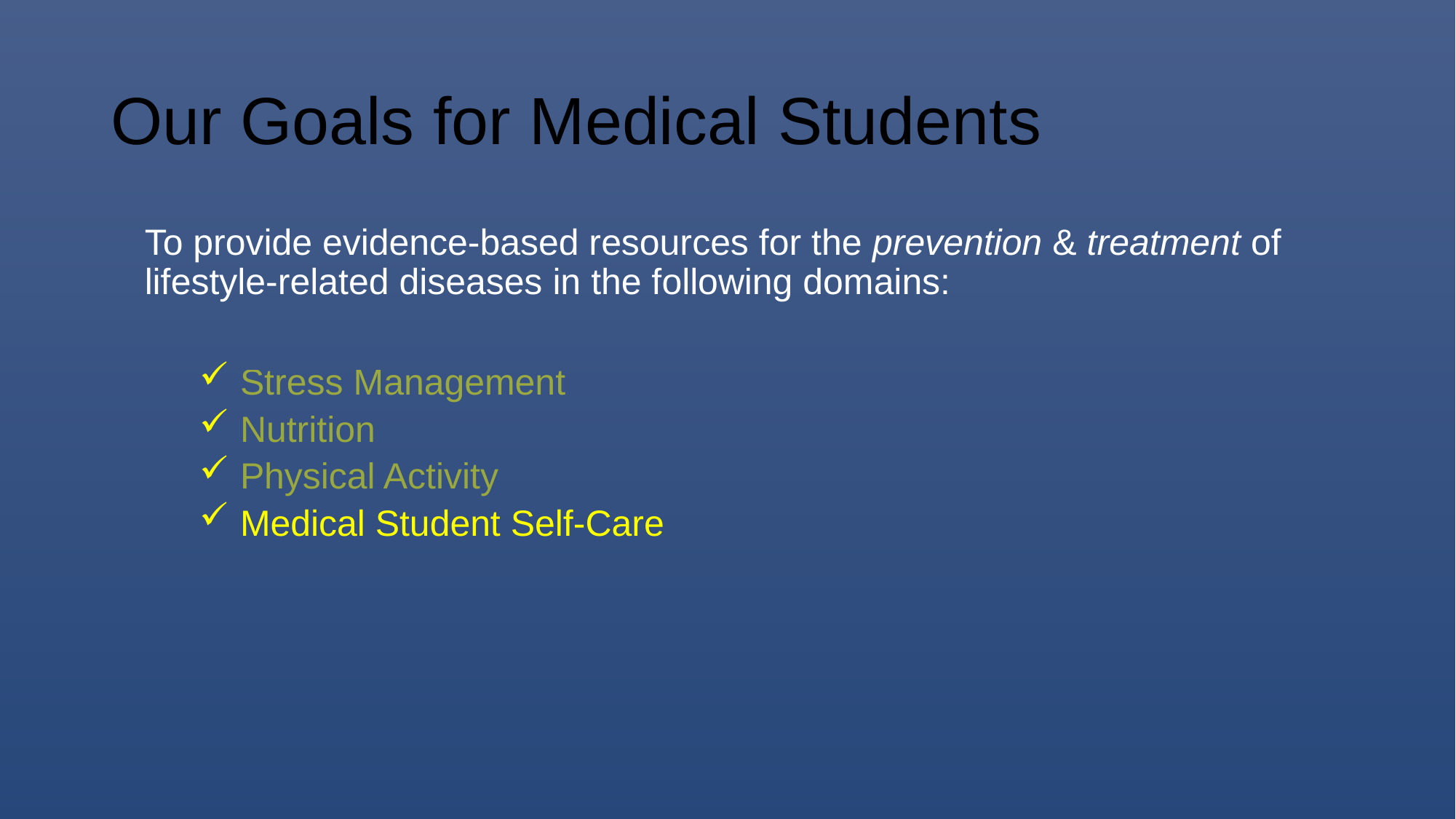

# Our Goals for Medical Students
To provide evidence-based resources for the prevention & treatment of lifestyle-related diseases in the following domains:
Stress Management
Nutrition
Physical Activity
Medical Student Self-Care

## Slide 9
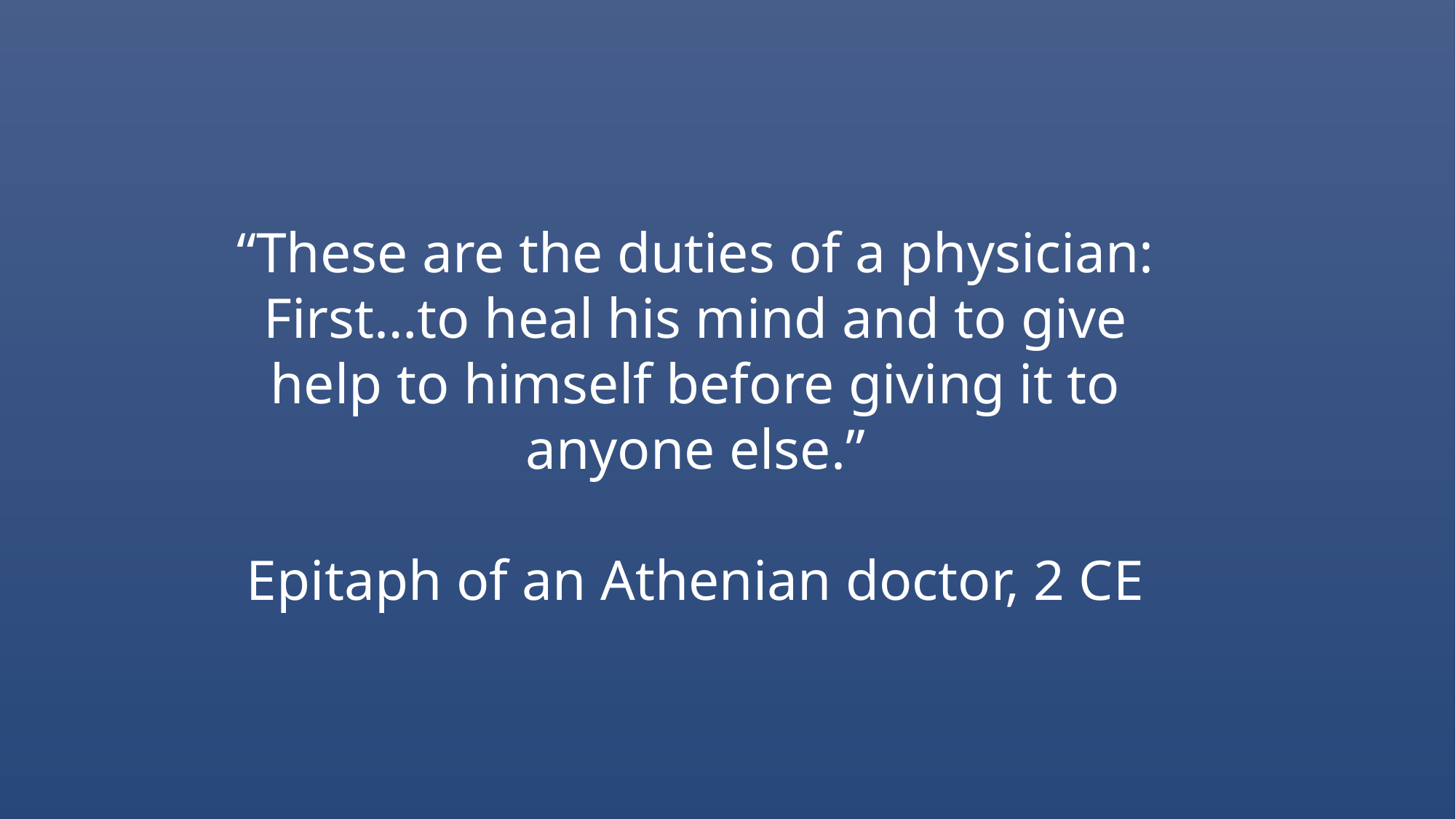

“These are the duties of a physician: First…to heal his mind and to give help to himself before giving it to anyone else.”
Epitaph of an Athenian doctor, 2 CE

## Slide 10
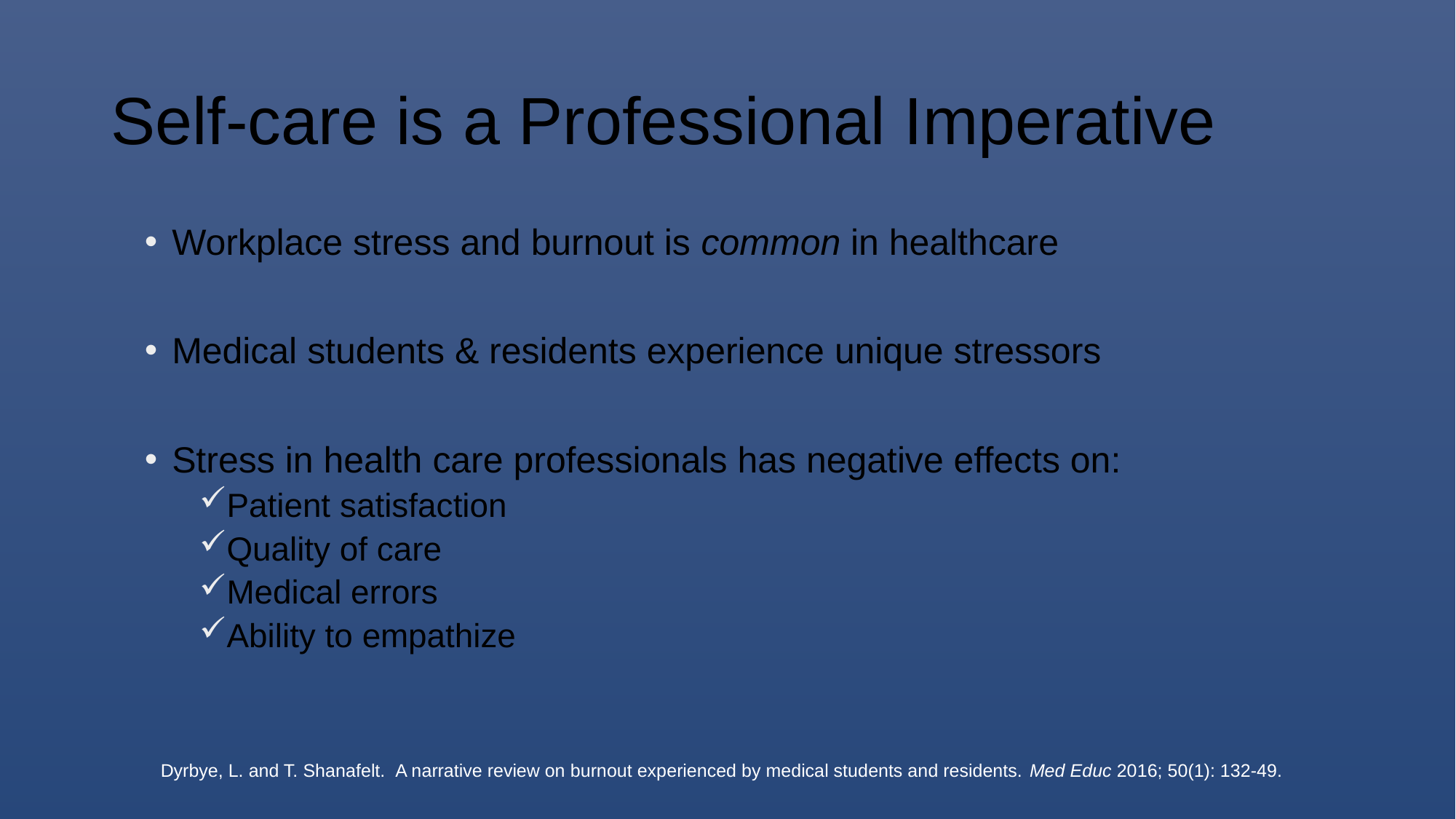

# Self-care is a Professional Imperative
Workplace stress and burnout is common in healthcare
Medical students & residents experience unique stressors
Stress in health care professionals has negative effects on:
Patient satisfaction
Quality of care
Medical errors
Ability to empathize
Dyrbye, L. and T. Shanafelt.  A narrative review on burnout experienced by medical students and residents. Med Educ 2016; 50(1): 132-49.

## Slide 11
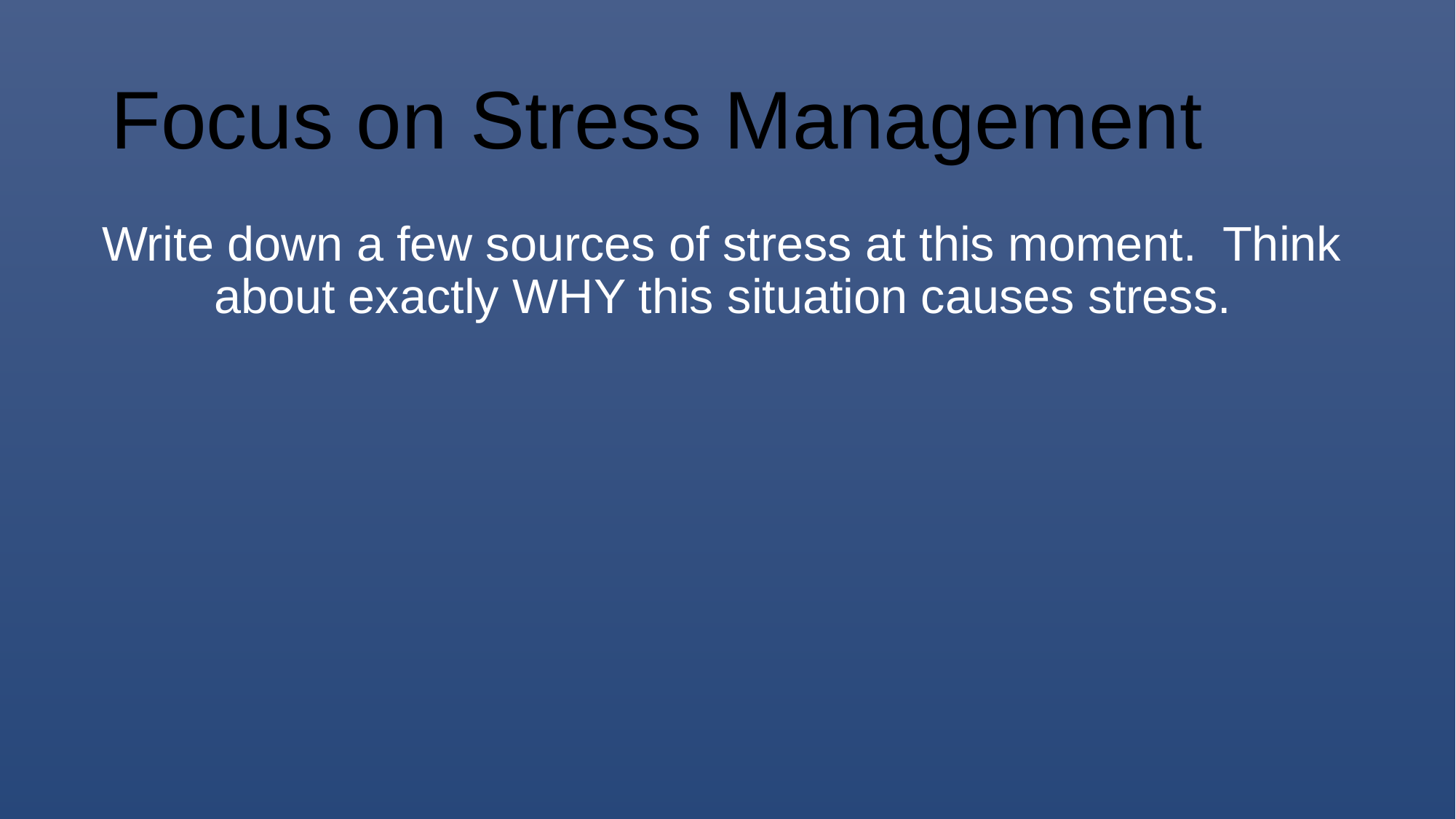

# Focus on Stress Management
Write down a few sources of stress at this moment.  Think about exactly WHY this situation causes stress.

## Slide 12
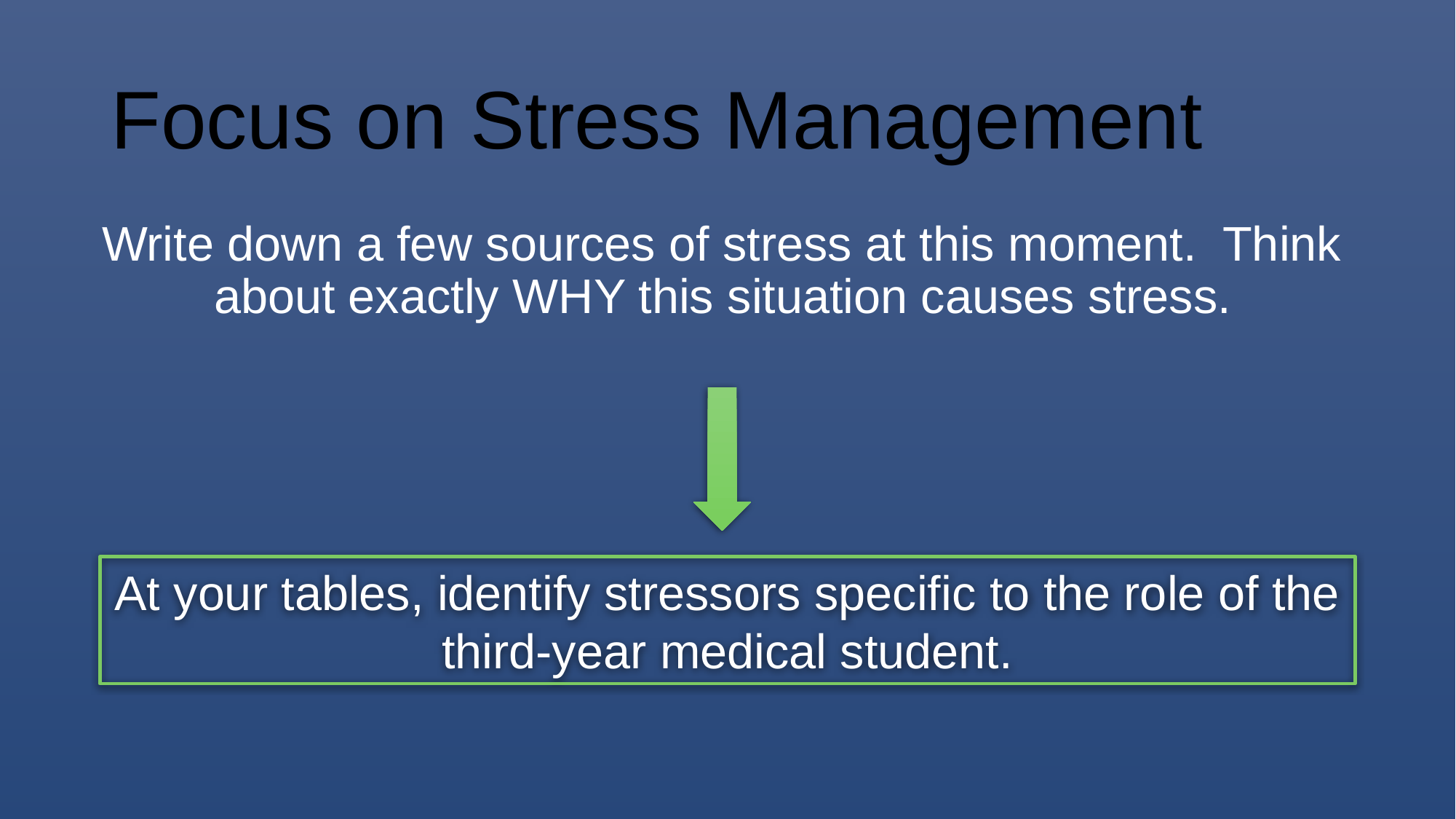

# Focus on Stress Management
Write down a few sources of stress at this moment.  Think about exactly WHY this situation causes stress.
At your tables, identify stressors specific to the role of the third-year medical student.

## Slide 13
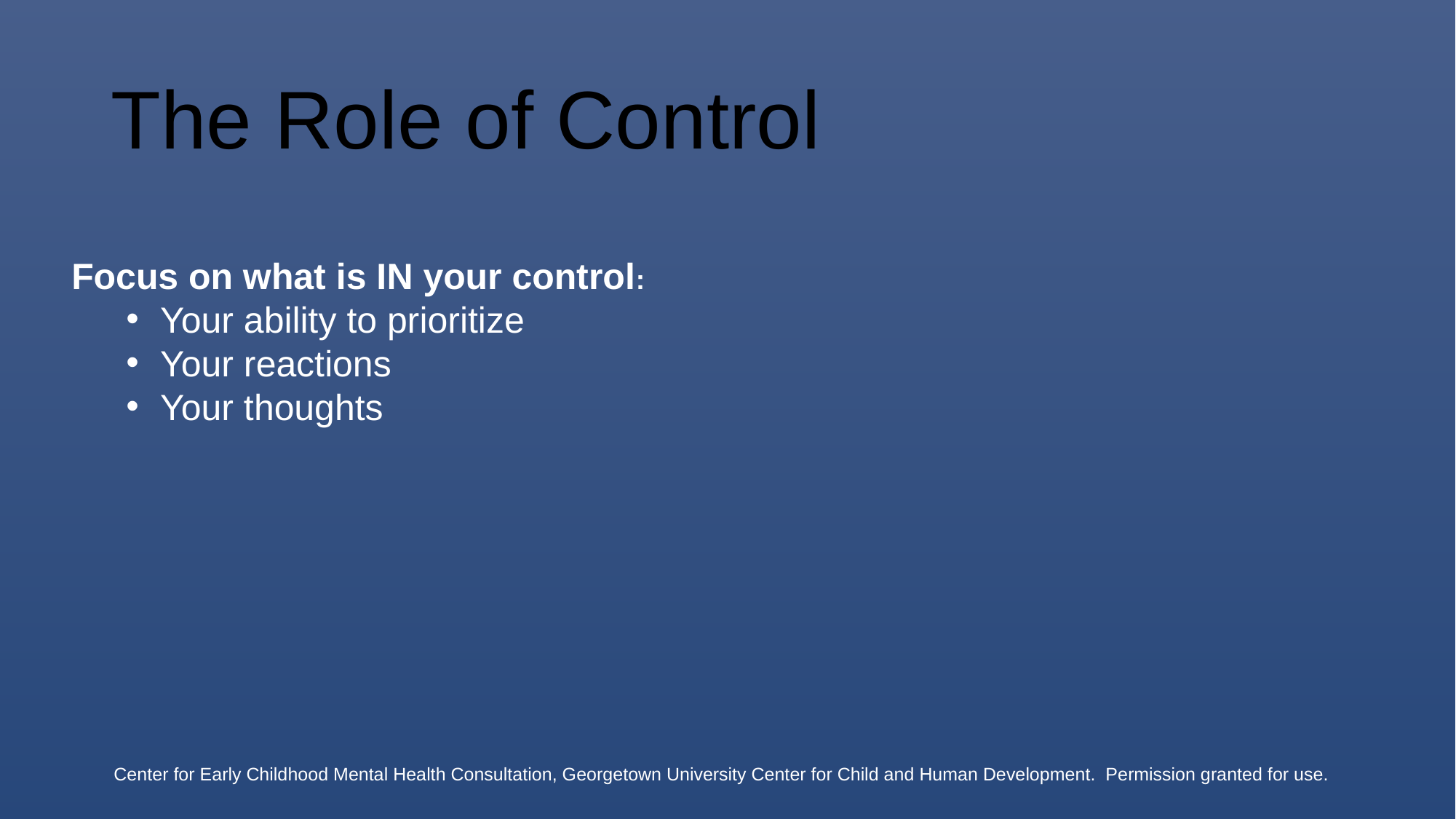

# The Role of Control
Focus on what is IN your control:
Your ability to prioritize
Your reactions
Your thoughts
Center for Early Childhood Mental Health Consultation, Georgetown University Center for Child and Human Development.  Permission granted for use.

## Slide 14
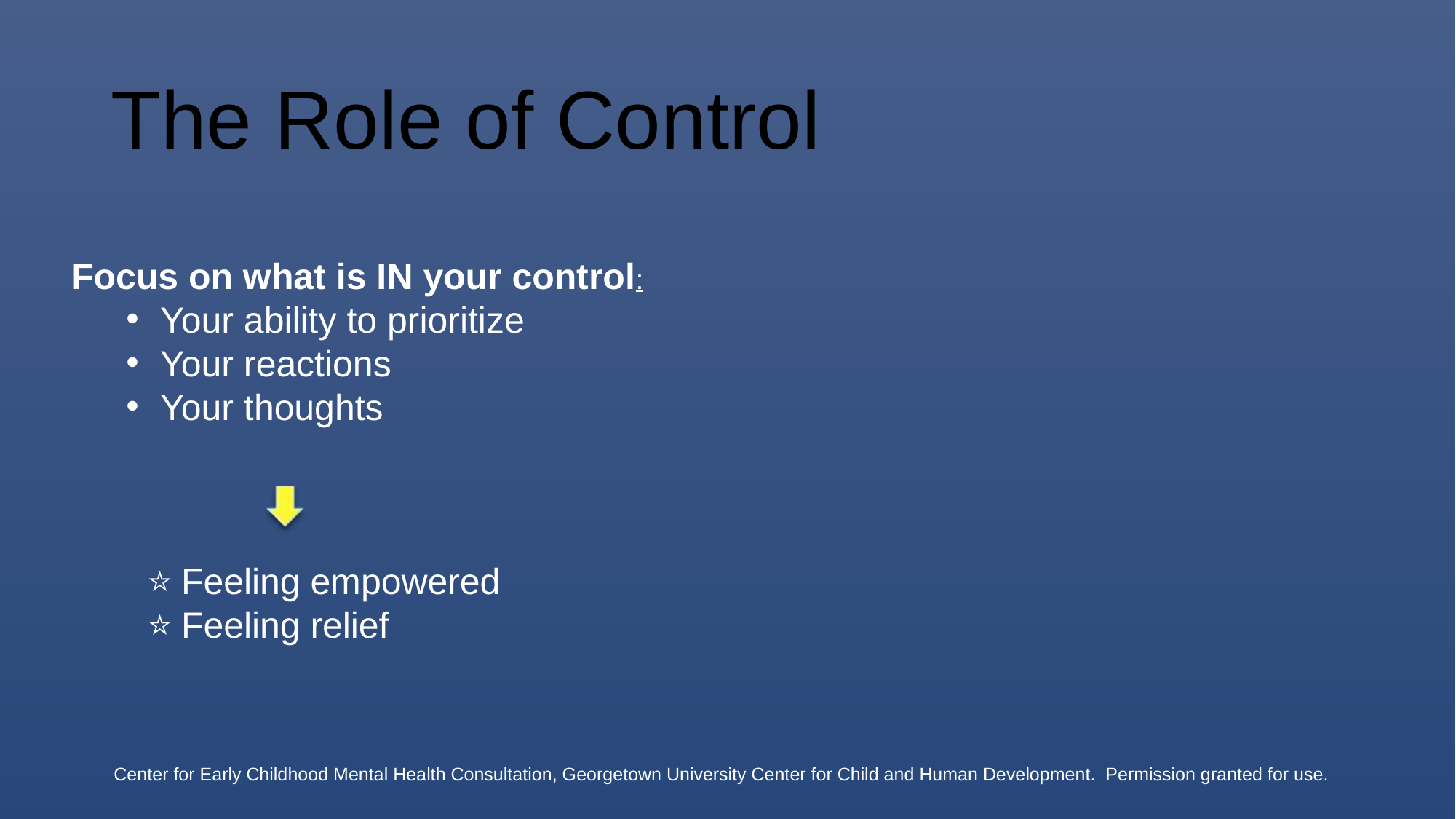

# The Role of Control
Focus on what is IN your control:
Your ability to prioritize
Your reactions
Your thoughts
Feeling empowered
Feeling relief
Center for Early Childhood Mental Health Consultation, Georgetown University Center for Child and Human Development.  Permission granted for use.

## Slide 15
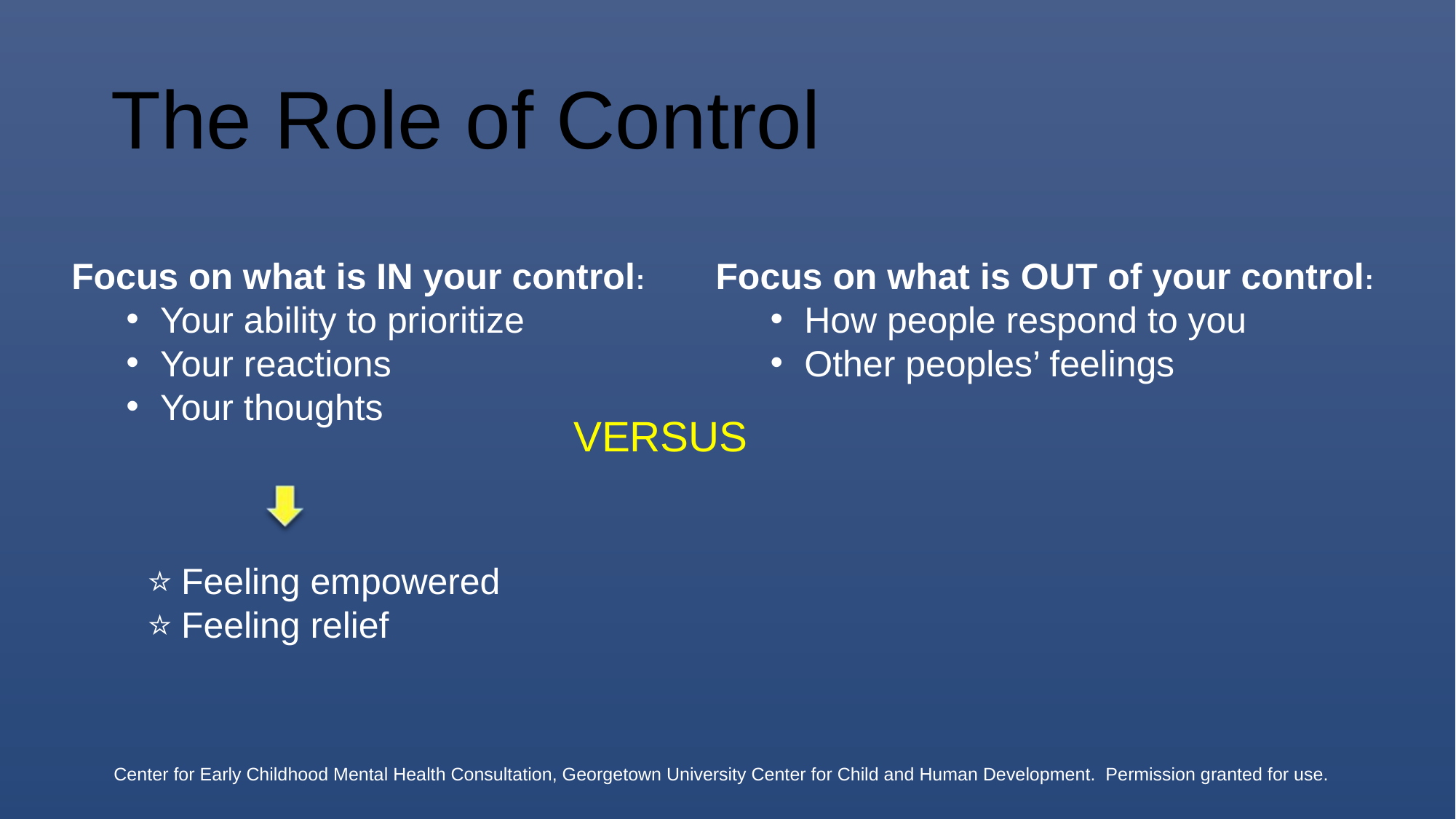

# The Role of Control
Focus on what is IN your control:
Your ability to prioritize
Your reactions
Your thoughts
Focus on what is OUT of your control:
How people respond to you
Other peoples’ feelings
VERSUS
Feeling empowered
Feeling relief
Center for Early Childhood Mental Health Consultation, Georgetown University Center for Child and Human Development.  Permission granted for use.

## Slide 16
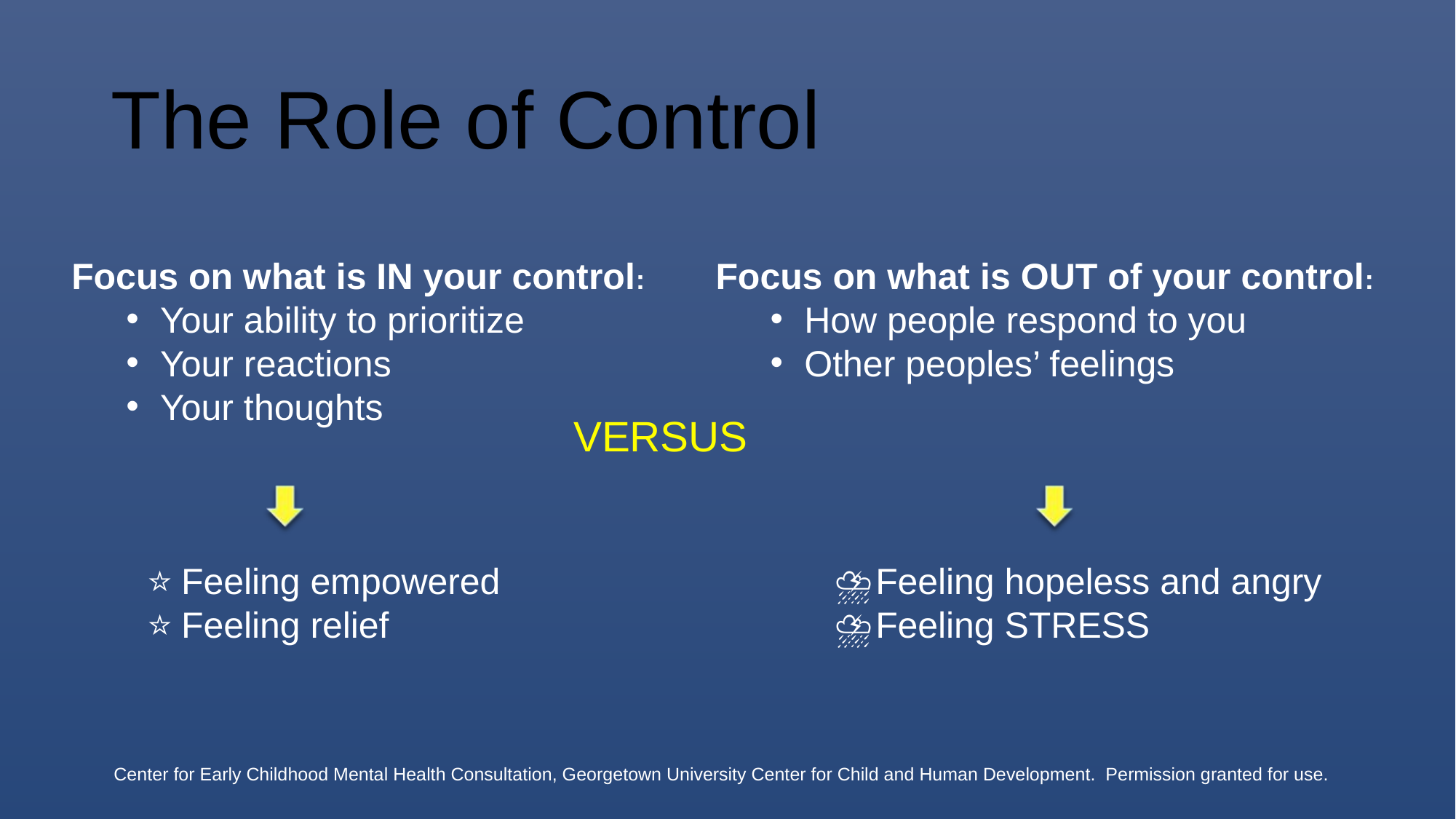

# The Role of Control
Focus on what is IN your control:
Your ability to prioritize
Your reactions
Your thoughts
Focus on what is OUT of your control:
How people respond to you
Other peoples’ feelings
VERSUS
Feeling empowered
Feeling relief
Feeling hopeless and angry
Feeling STRESS
Center for Early Childhood Mental Health Consultation, Georgetown University Center for Child and Human Development.  Permission granted for use.

## Slide 17
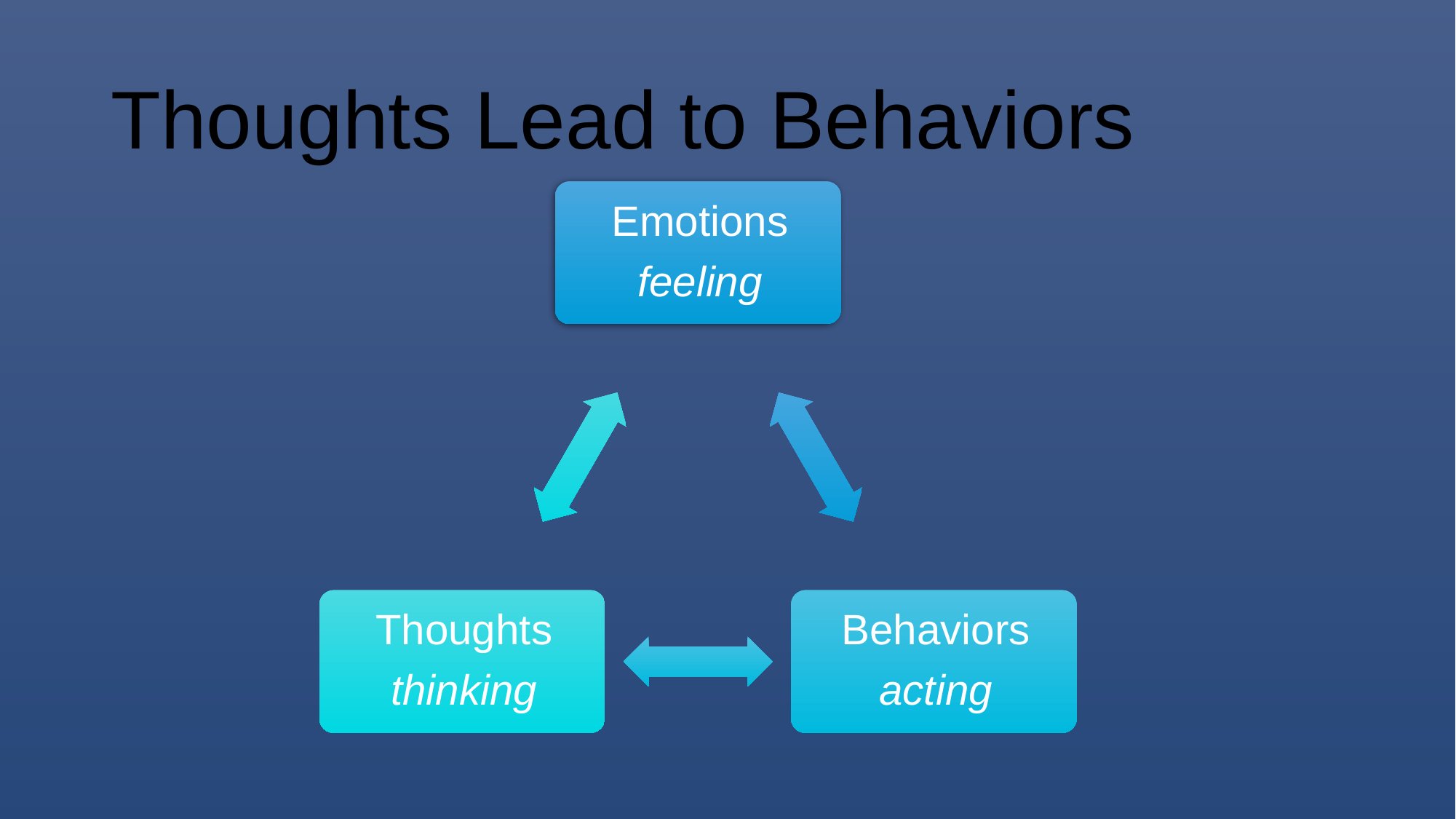

# Thoughts Lead to Behaviors

## Slide 18
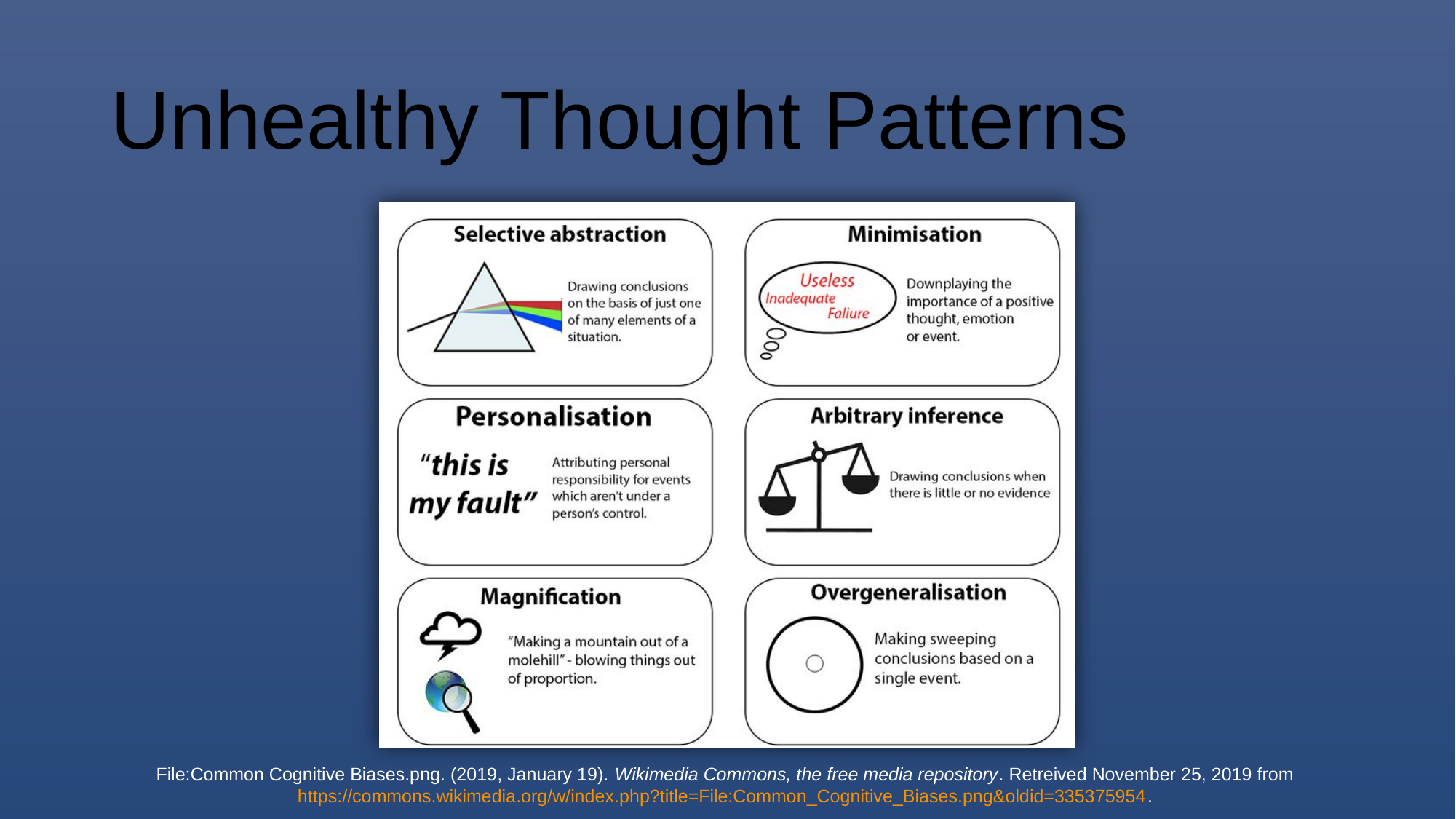

# Unhealthy Thought Patterns
File:Common Cognitive Biases.png. (2019, January 19). Wikimedia Commons, the free media repository. Retreived November 25, 2019 from https://commons.wikimedia.org/w/index.php?title=File:Common_Cognitive_Biases.png&oldid=335375954.

## Slide 19
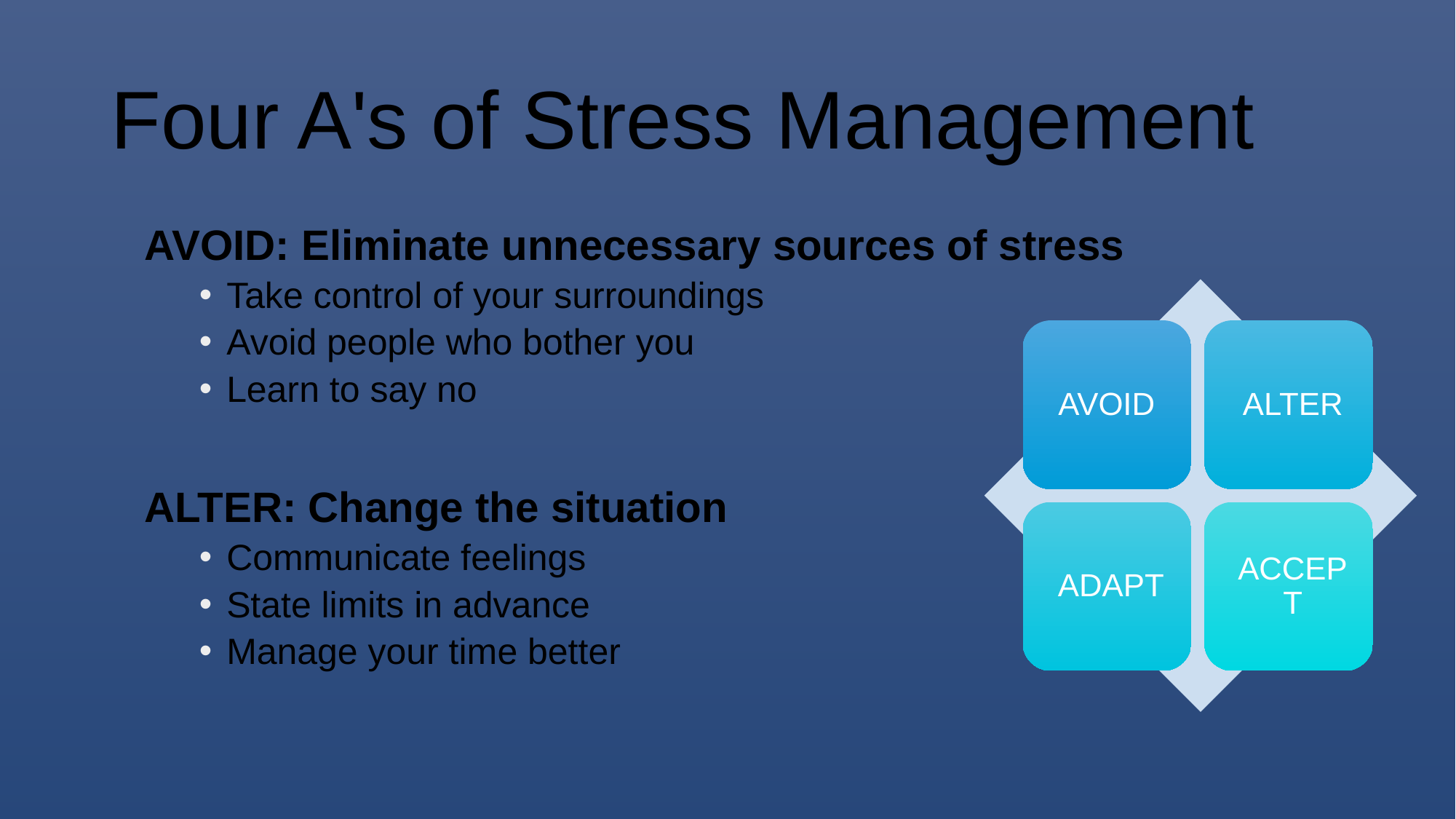

# Four A's of Stress Management
AVOID: Eliminate unnecessary sources of stress
Take control of your surroundings
Avoid people who bother you
Learn to say no
ALTER: Change the situation
Communicate feelings
State limits in advance
Manage your time better

## Slide 20
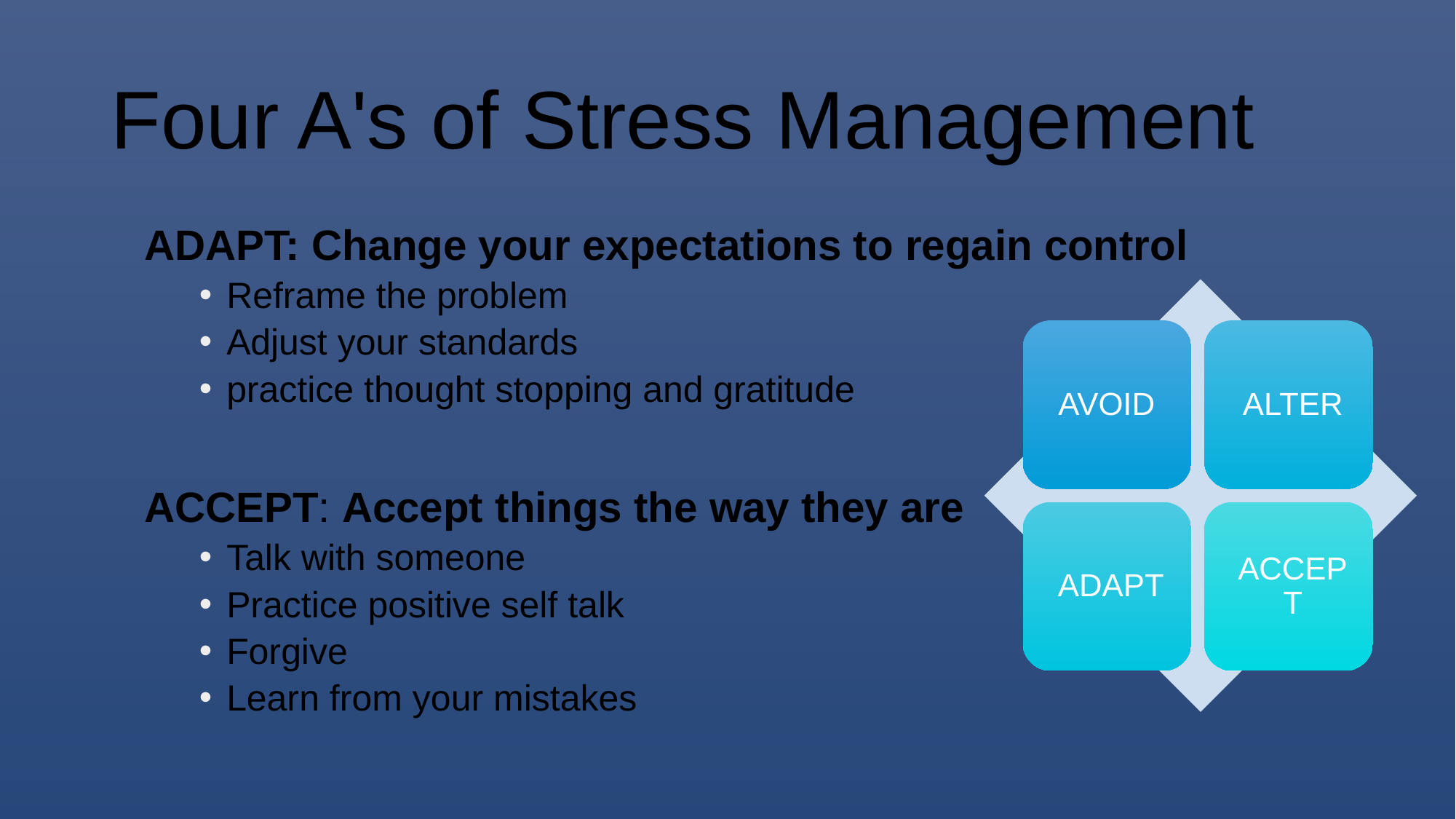

# Four A's of Stress Management
ADAPT: Change your expectations to regain control
Reframe the problem
Adjust your standards
practice thought stopping and gratitude
ACCEPT: Accept things the way they are
Talk with someone
Practice positive self talk
Forgive
Learn from your mistakes

## Slide 21
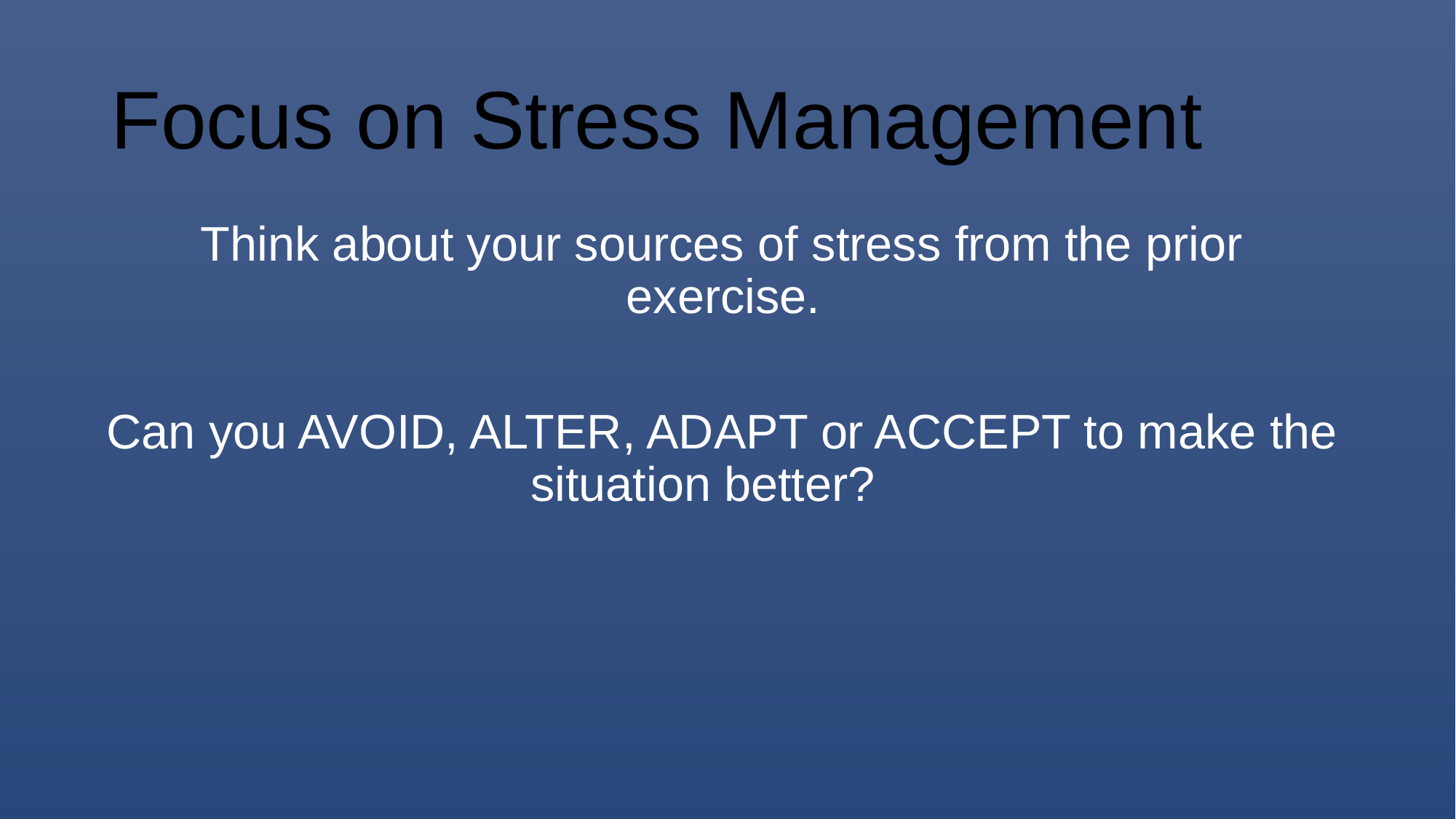

# Focus on Stress Management
Think about your sources of stress from the prior exercise.
Can you AVOID, ALTER, ADAPT or ACCEPT to make the situation better?

## Slide 22
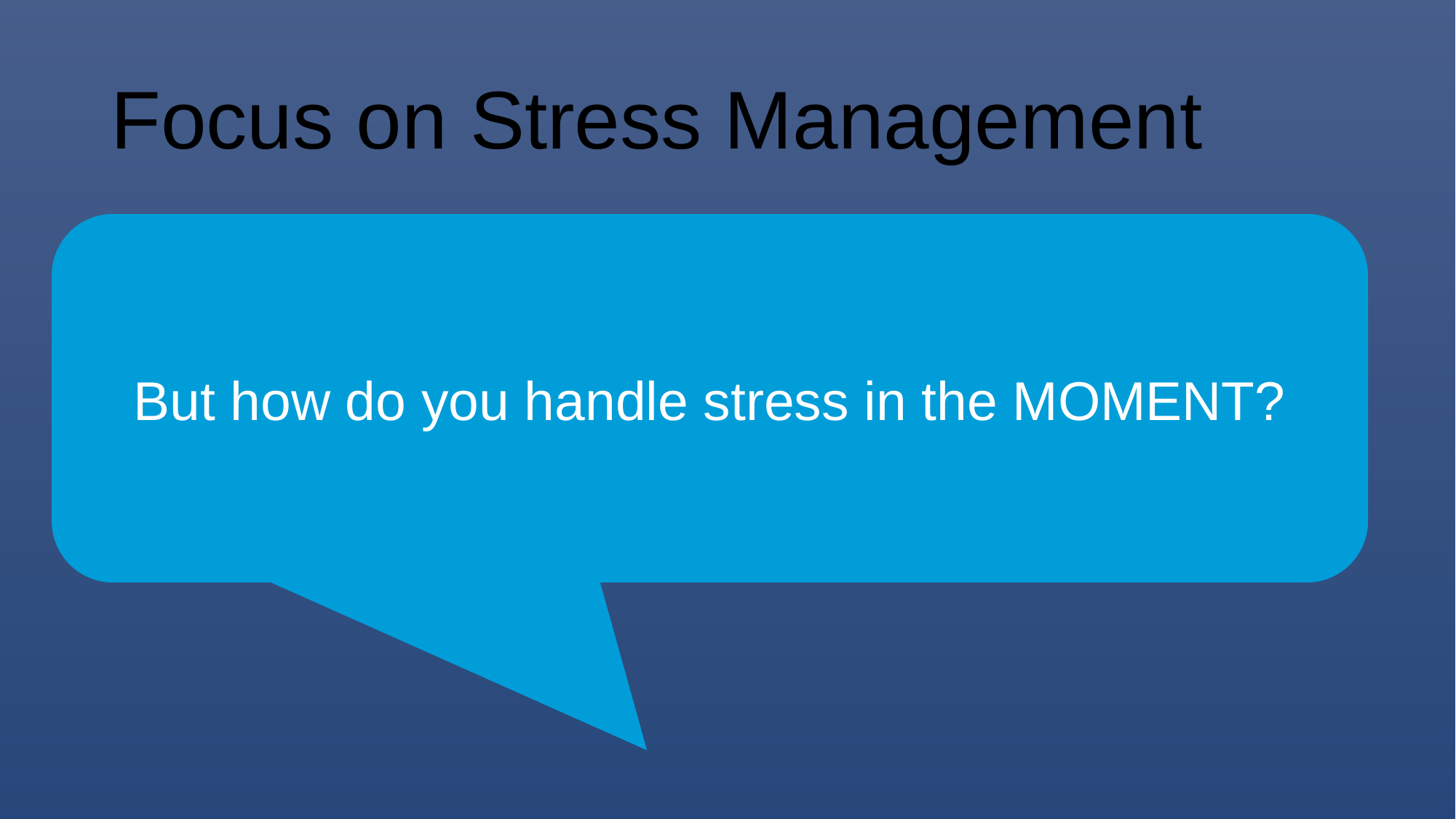

# Focus on Stress Management
Think about your sources of stress from the prior exercise.
Can you AVOID, ALTER, ADAPT or ACCEPT to make the situation better?
But how do you handle stress in the MOMENT?

## Slide 23
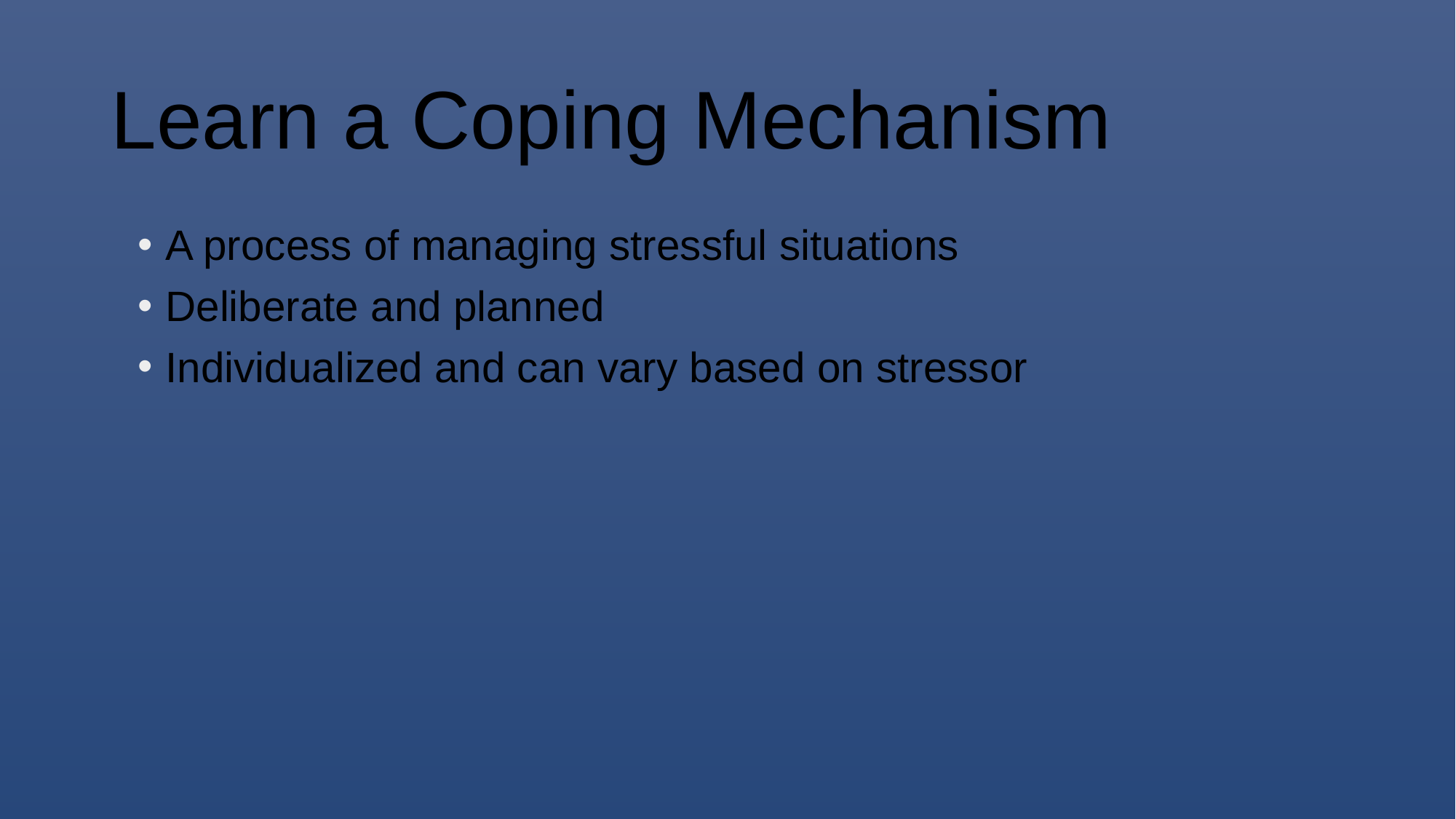

# Learn a Coping Mechanism
A process of managing stressful situations
Deliberate and planned
Individualized and can vary based on stressor

## Slide 24
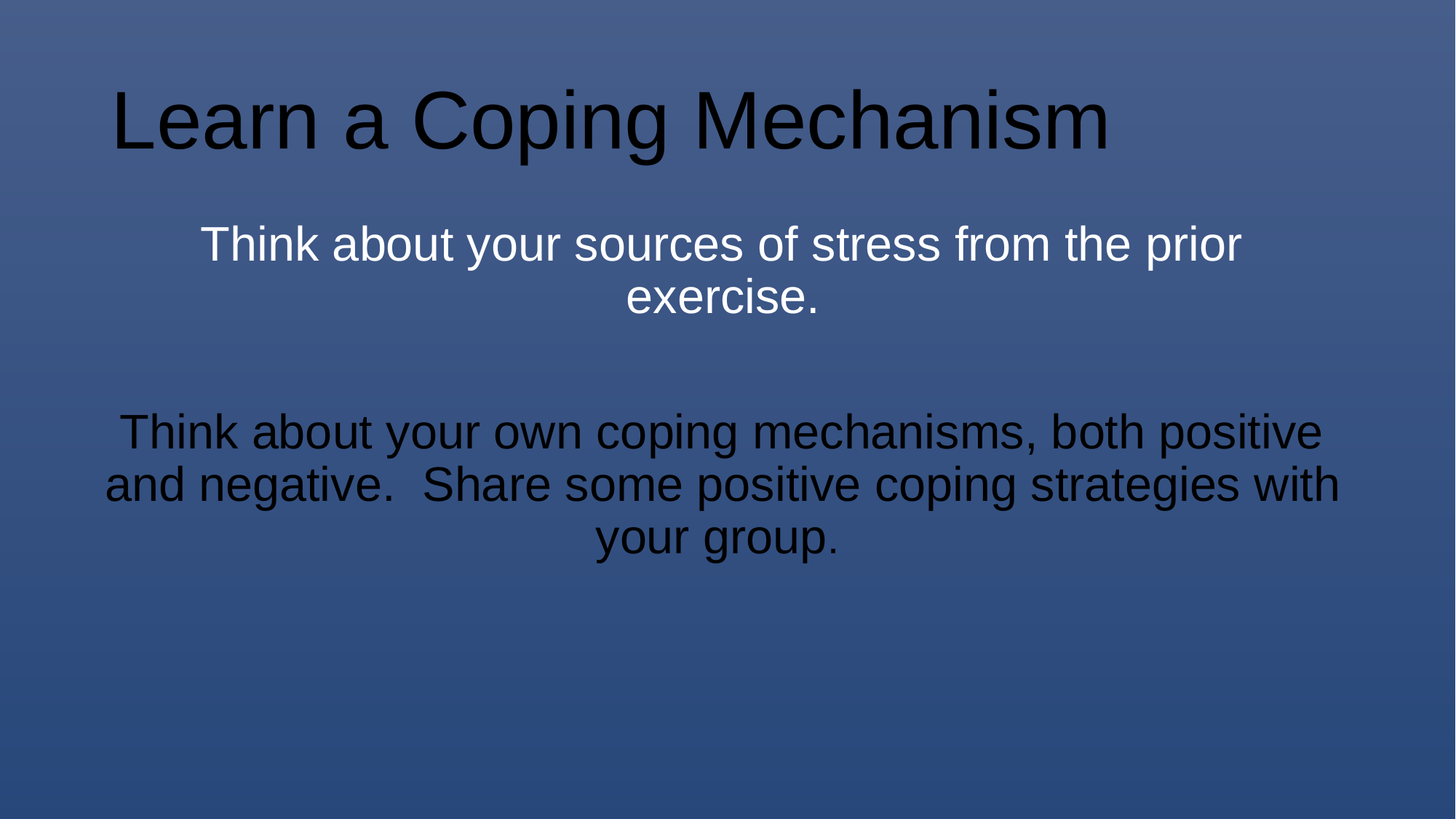

# Learn a Coping Mechanism
Think about your sources of stress from the prior exercise.
Think about your own coping mechanisms, both positive and negative.  Share some positive coping strategies with your group.

## Slide 25
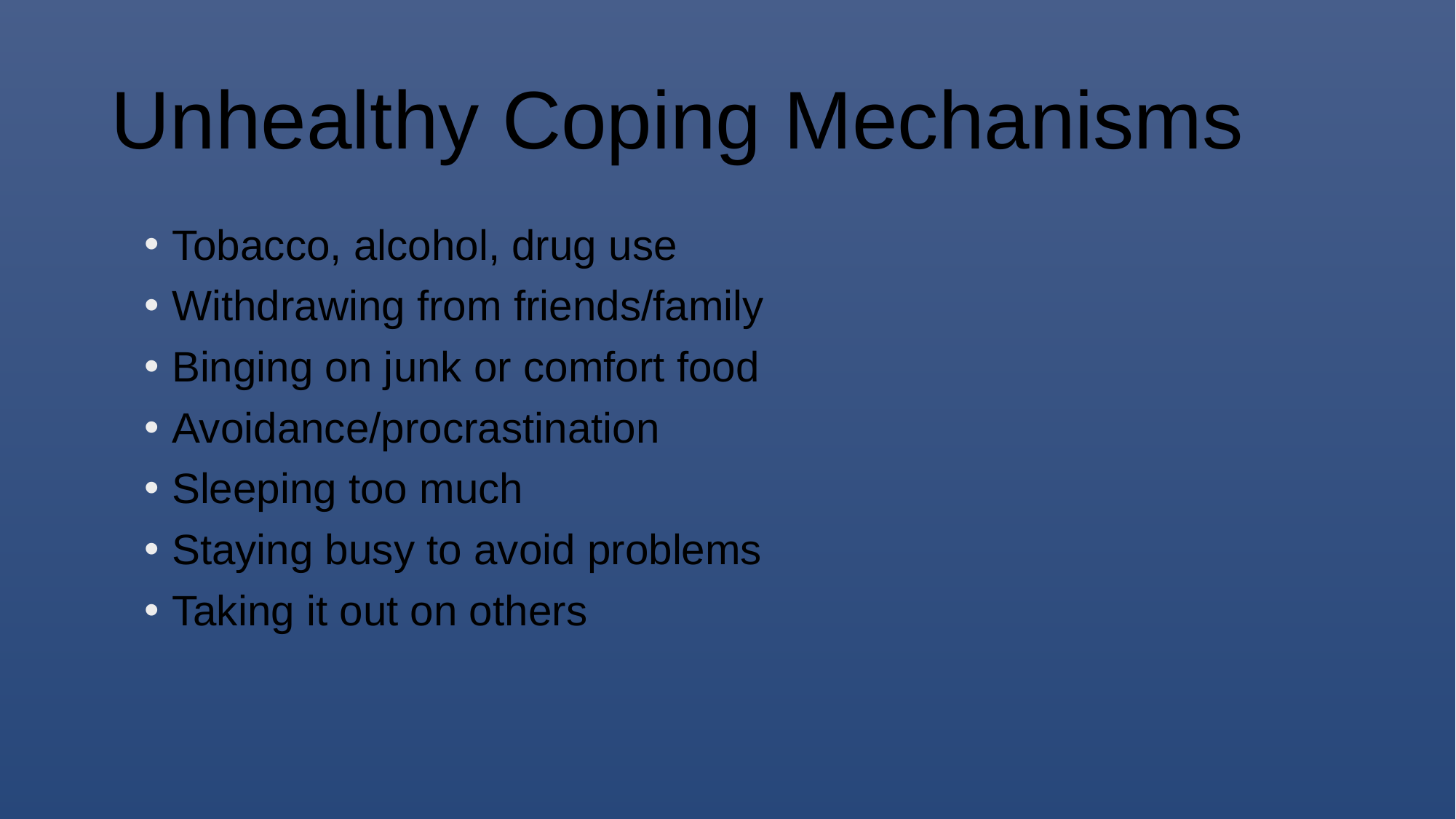

# Unhealthy Coping Mechanisms
Tobacco, alcohol, drug use
Withdrawing from friends/family
Binging on junk or comfort food
Avoidance/procrastination
Sleeping too much
Staying busy to avoid problems
Taking it out on others

## Slide 26
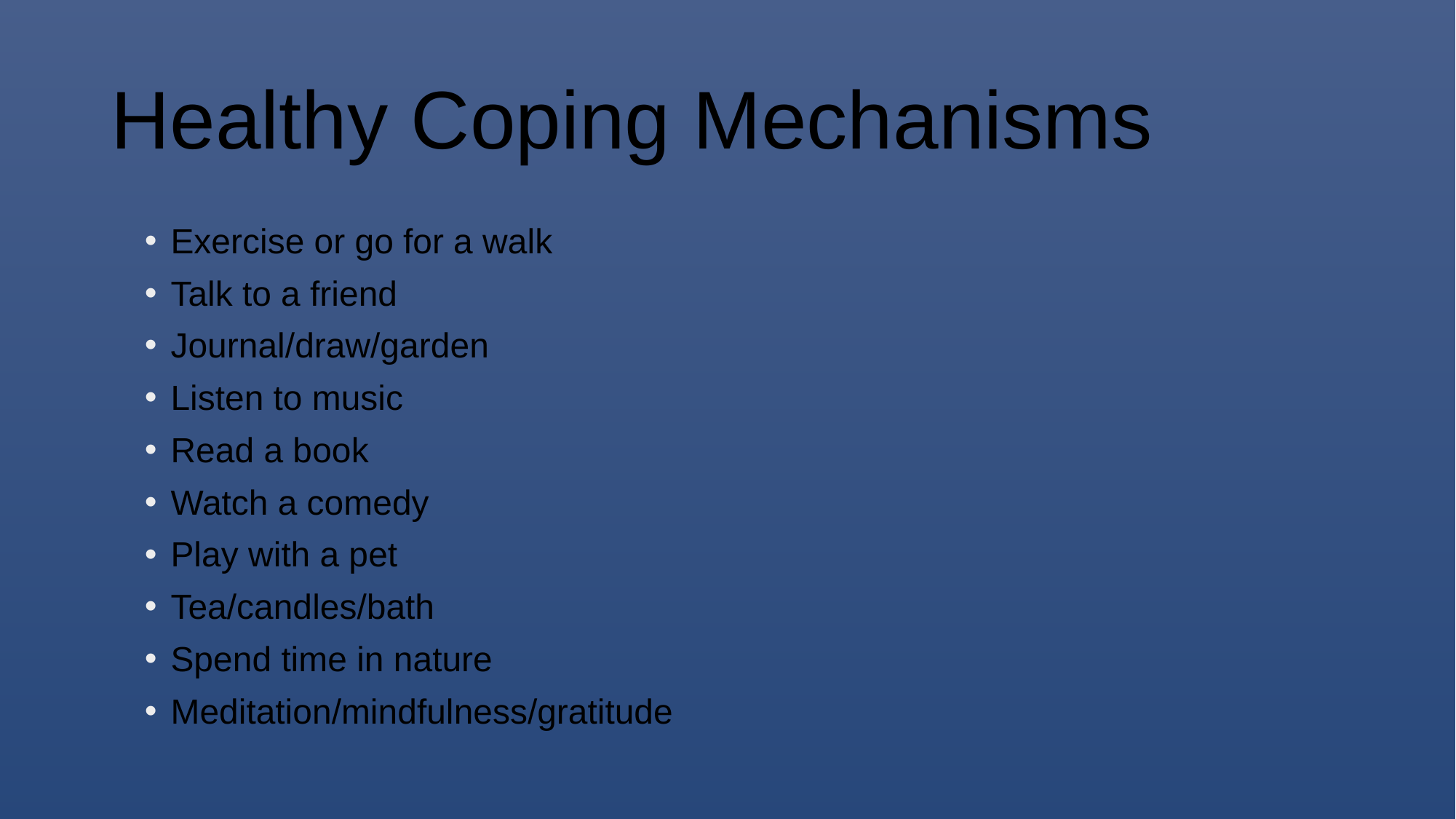

# Healthy Coping Mechanisms
Exercise or go for a walk
Talk to a friend
Journal/draw/garden
Listen to music
Read a book
Watch a comedy
Play with a pet
Tea/candles/bath
Spend time in nature
Meditation/mindfulness/gratitude

## Slide 27
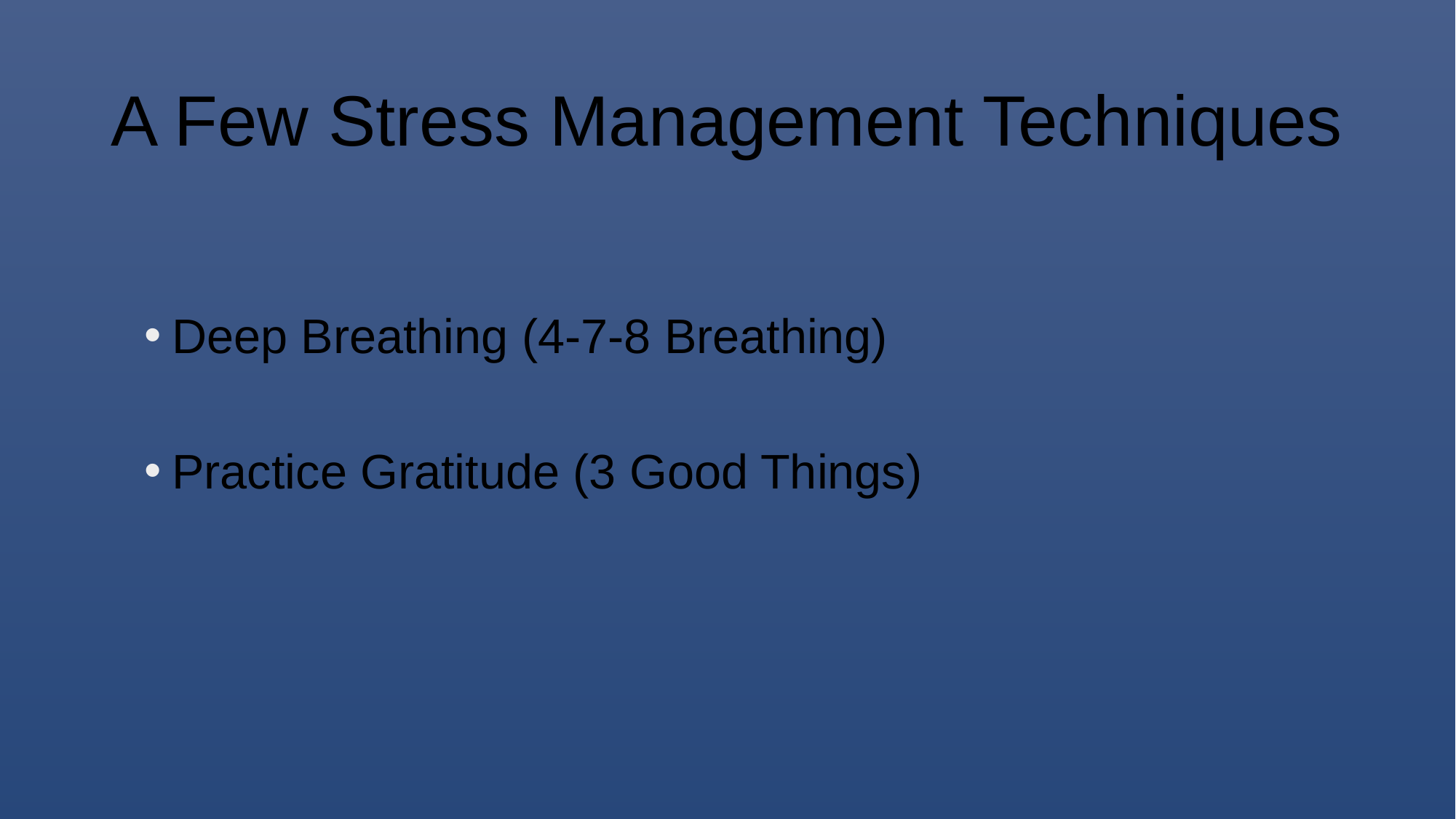

# A Few Stress Management Techniques
Deep Breathing (4-7-8 Breathing)
Practice Gratitude (3 Good Things)

## Slide 28
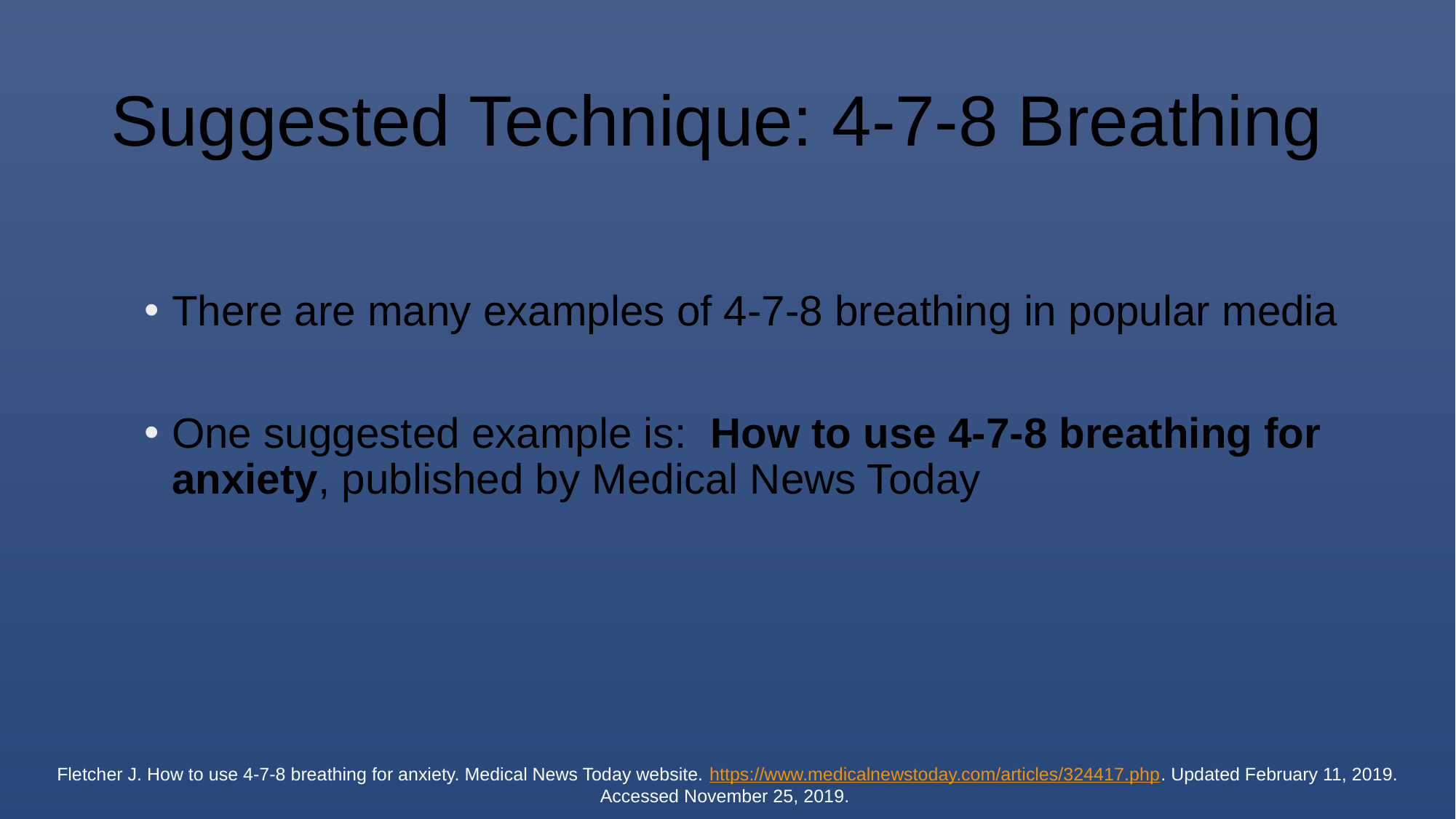

# Suggested Technique: 4-7-8 Breathing
There are many examples of 4-7-8 breathing in popular media
One suggested example is: How to use 4-7-8 breathing for anxiety, published by Medical News Today
Fletcher J. How to use 4-7-8 breathing for anxiety. Medical News Today website. https://www.medicalnewstoday.com/articles/324417.php. Updated February 11, 2019. Accessed November 25, 2019.

## Slide 29
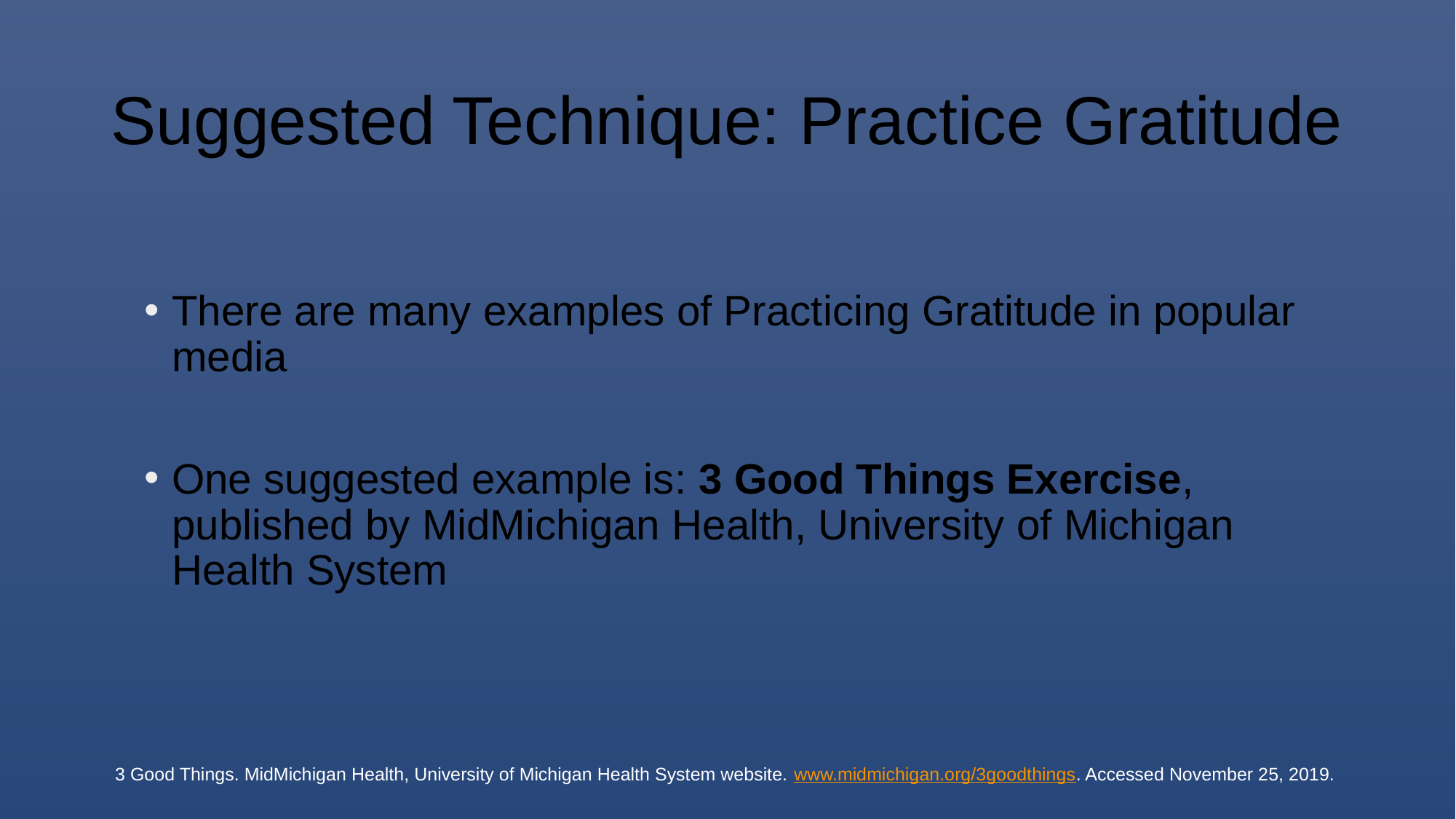

# Suggested Technique: Practice Gratitude
There are many examples of Practicing Gratitude in popular media
One suggested example is: 3 Good Things Exercise, published by MidMichigan Health, University of Michigan Health System
3 Good Things. MidMichigan Health, University of Michigan Health System website. www.midmichigan.org/3goodthings. Accessed November 25, 2019.

## Slide 30
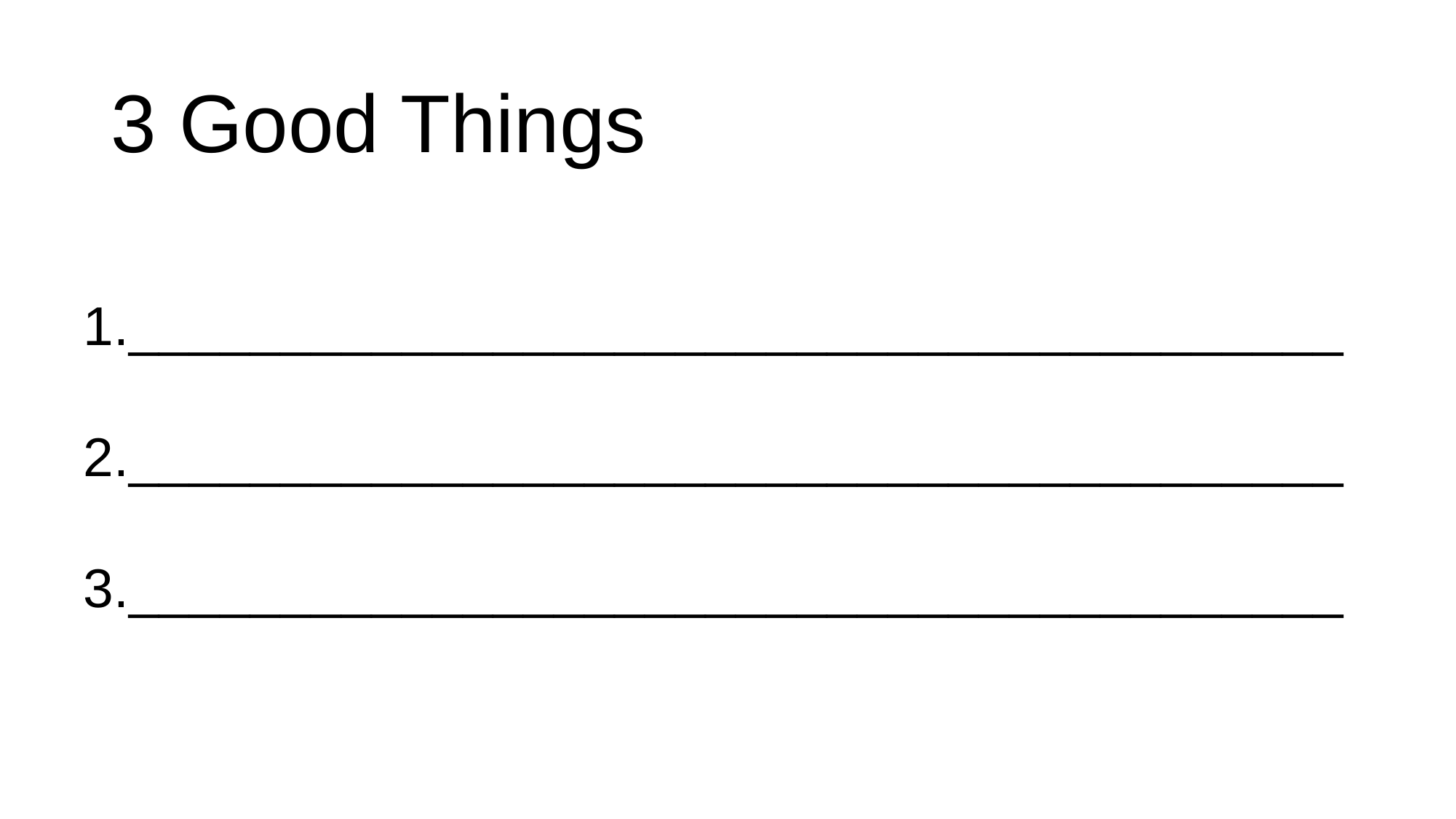

# 3 Good Things
________________________________________
________________________________________
________________________________________
Register for a study cohort: http://www.dukepatientsafetycenter.com/

## Slide 31
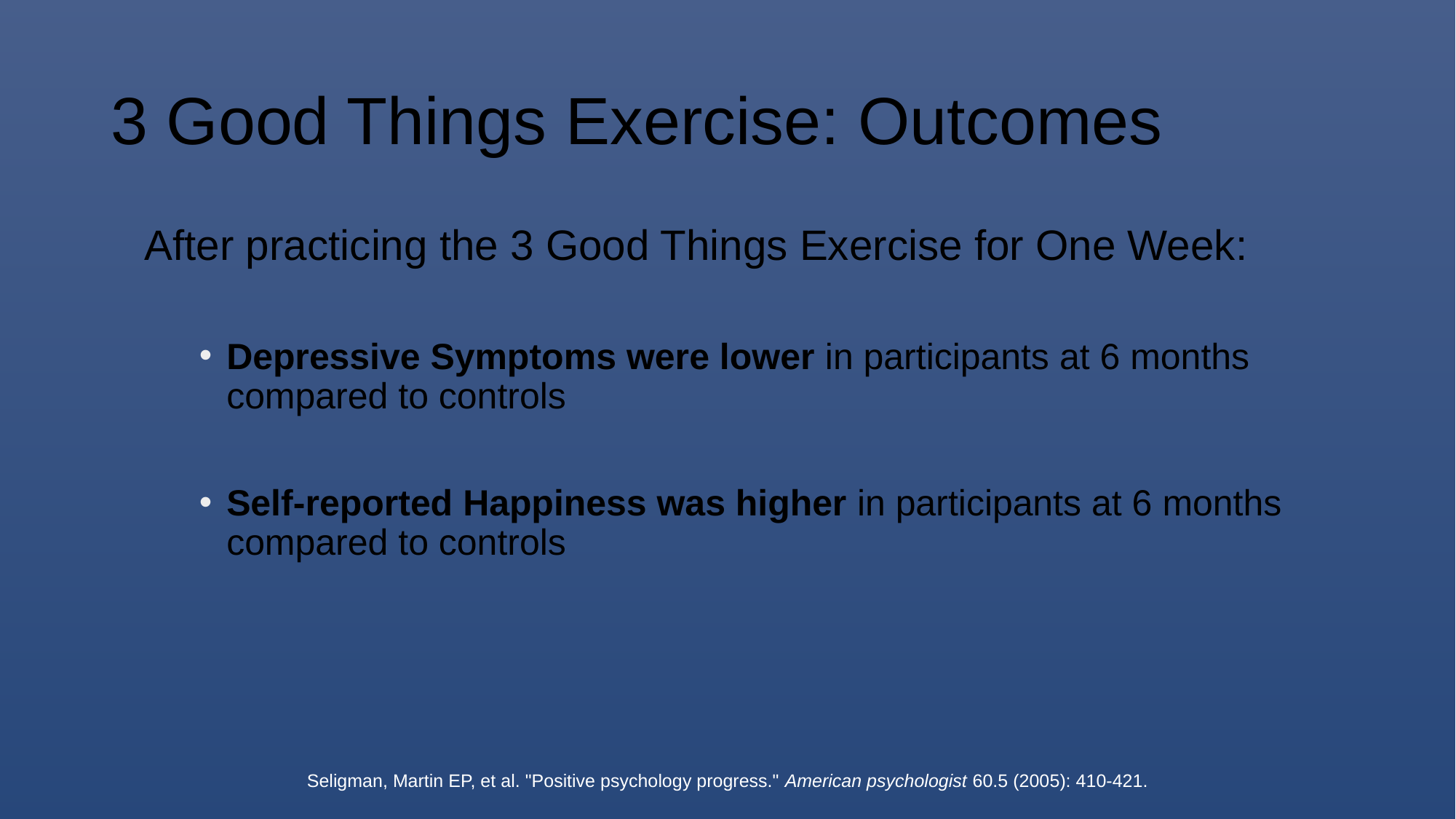

# 3 Good Things Exercise: Outcomes
After practicing the 3 Good Things Exercise for One Week:
Depressive Symptoms were lower in participants at 6 months compared to controls
Self-reported Happiness was higher in participants at 6 months compared to controls
Seligman, Martin EP, et al. "Positive psychology progress." American psychologist 60.5 (2005): 410-421.

## Slide 32
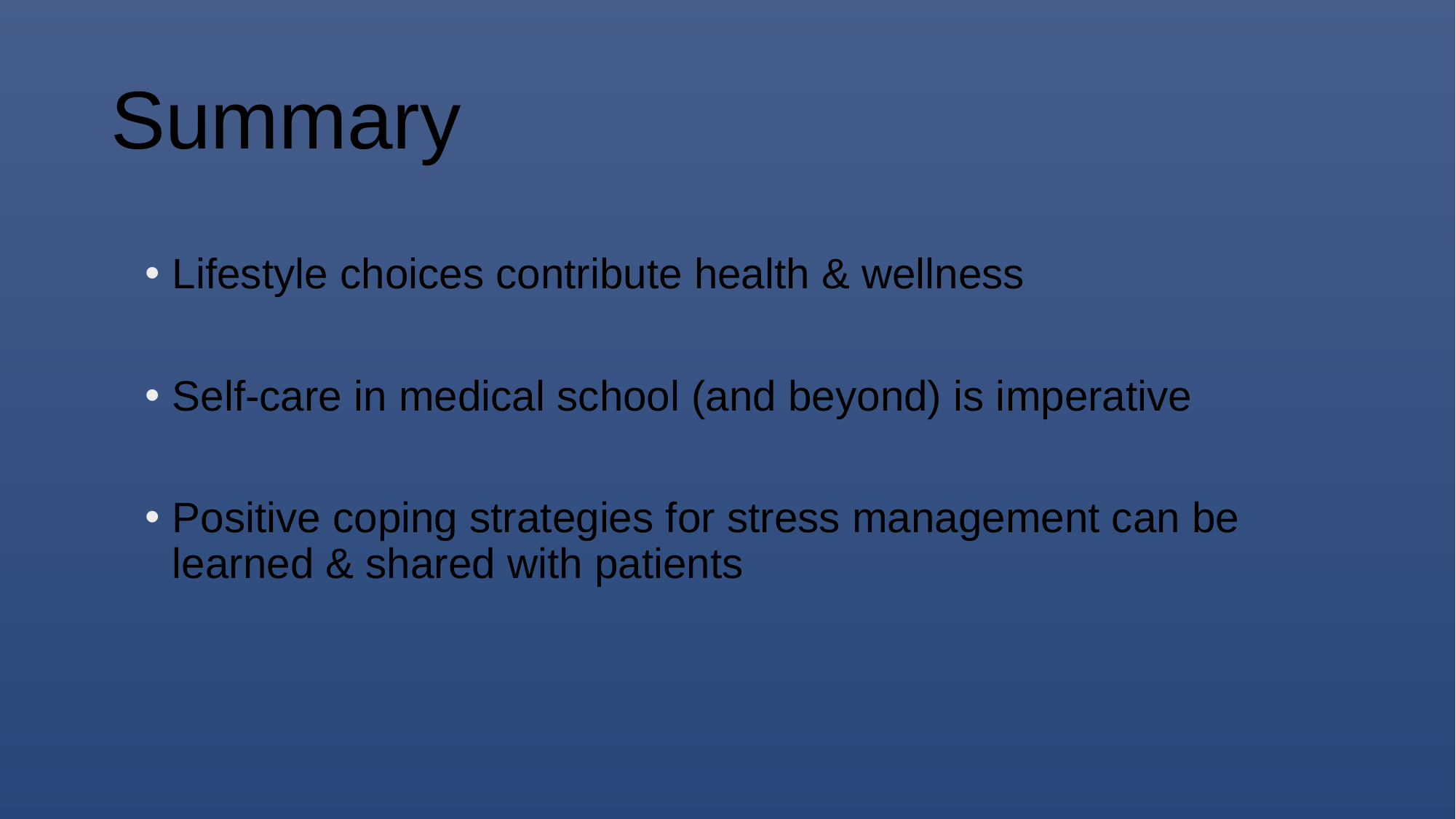

# Summary
Lifestyle choices contribute health & wellness
Self-care in medical school (and beyond) is imperative
Positive coping strategies for stress management can be learned & shared with patients
