# Supplementary material for: An Interactive Lifestyle Medicine Curriculum for Third-Year Medical Students to Promote Student and Patient Wellness
Source: MedEdPORTAL. 2020 Sep 18;16:10972. doi: 10.15766/mep_2374-8265.10972 (PMC7499809; doi:10.15766/mep_2374-8265.10972)
Supplement: Supplementary file 1 — Introduction & Stress Management Presentation.pptxIntroduction & Stress Management Facilitator Guide.docxUnhealthy Thoughts Handout.pdfGood Things Worksheet.pdfNutrition Presentation.pptxNutrition Facilitator Guide.docxPhysical Activity Presentation.pptxPhysical Activity Facilitator Guide.docxPresession Evaluation.docxPostsession Evaluation.docxSession Evaluation.docx [file mep_2374-8265.10972-s001.zip › B. Introduction & Stress Management Facilitator Guide.docx]

**Facilitator Guide**

**Lifestyle Medicine and Student Wellness: Stress Management**

Materials needed:

- Board/Flip Chart
- Talk Back to Your Unhelpful Thoughts handout
- 3 Good Things Handout

Slides 1-10: Introduction to Lifestyle Medicine (10 minutes)

Slides 11-12: Stress Management Activity: Students should take a few minutes to write down some sources of stress for themselves and try to identify why that specific issue is causing stress. They should then spend five minutes in a table discussion about sources of stress that are unique to them as third year medical students. (10 minutes)

Slides 13-20: The Role of Control in Stress Management (10 minutes)

Slides 21-22: Stress Management Activity: Students should take a few minutes to reflect on their personal source of stress and decide which of the four As can apply. Distribute the “Talk Back to Your Unhelpful Thoughts” handout from the Center for Early Childhood Mental Health Consultation at the Georgetown University Center for Child and Human Development. They should review this handout and identify any unhealthy thought patterns that may contribute to their stress. (5 minutes)

Slide 23: Introduce Coping Strategies (2 minutes)

Slide 24: Coping Strategy Activity: Have students spend a few minutes thinking about their coping mechanisms, and then a few more minutes sharing positive coping strategies with their table. Bring it back to the larger group and have them share these strategies, while writing them down on the board/flip chart/etc.

Slides 25-27: Discuss Healthy vs. Unhealthy Coping Mechanisms (3 minutes)

Slide 28: Suggested Deep Breathing Activity: Ask the students to sit comfortably and quietly for this exercise. At this point we suggest projecting a video example of 4-7-8 breathing, and guiding the students through the exercise. There are many examples available on public media sites.  (2 minutes)

Slides 29-30: Suggested Practice Gratitude Activity: At this point we suggest using the worksheet in Appendix K to guide the students through this exercise. (5 minutes)

Slide 31: “3 Good Things” Exercise Outcomes. (2 minutes)

Slide 32: Summary (5 minutes to allow time for questions)
