# Supplementary material for: An Interactive Lifestyle Medicine Curriculum for Third-Year Medical Students to Promote Student and Patient Wellness
Source: MedEdPORTAL. 2020 Sep 18;16:10972. doi: 10.15766/mep_2374-8265.10972 (PMC7499809; doi:10.15766/mep_2374-8265.10972)
Supplement: Supplementary file 1 — Introduction & Stress Management Presentation.pptxIntroduction & Stress Management Facilitator Guide.docxUnhealthy Thoughts Handout.pdfGood Things Worksheet.pdfNutrition Presentation.pptxNutrition Facilitator Guide.docxPhysical Activity Presentation.pptxPhysical Activity Facilitator Guide.docxPresession Evaluation.docxPostsession Evaluation.docxSession Evaluation.docx [file mep_2374-8265.10972-s001.zip › C. Unhealthy Thoughts Handout.pdf]

STRESS

Talk Back to Your Unhelpful **Thoughts**

Stress comes from our perception of the situation. Technically, the actual situation is not stressful; it is our **PERCEPTION** that makes it stressful. Here are some common unhelpful patterns of thinking that we all have as well as ways you can think about challenging these thoughts.

All or Nothing Thinking:

You see things in black-and-white categories. If your actions aren't perfect then they are seen as a failure.

Challenge: Instead of thinking in an "either-or" way, try to think in shades of gray. Evaluate the situation on a scale of 0-10. Think again about partial success and reevaluate, on a scale of 0-10.

Filtering out the Positive:

You focus in on one thing that went wrong and filter out the positive events that occurred.

Challenge: Try to be as kind to yourself as you would be with a friend. Review the day's events and focus on all of the positive things that went right. For every negative thing you think of, try to also focus on one positive thing.

Overgeneralization:

You see a single negative situation as never-ending and a "forever" pattern.

Challenge: Remind yourself that a single negative event (or even multiple occasions) doesn't mean it will truly last forever. Think of a specific time when a single negative experience did not have a long lasting outcome.

— BURNS, D.D. 1989

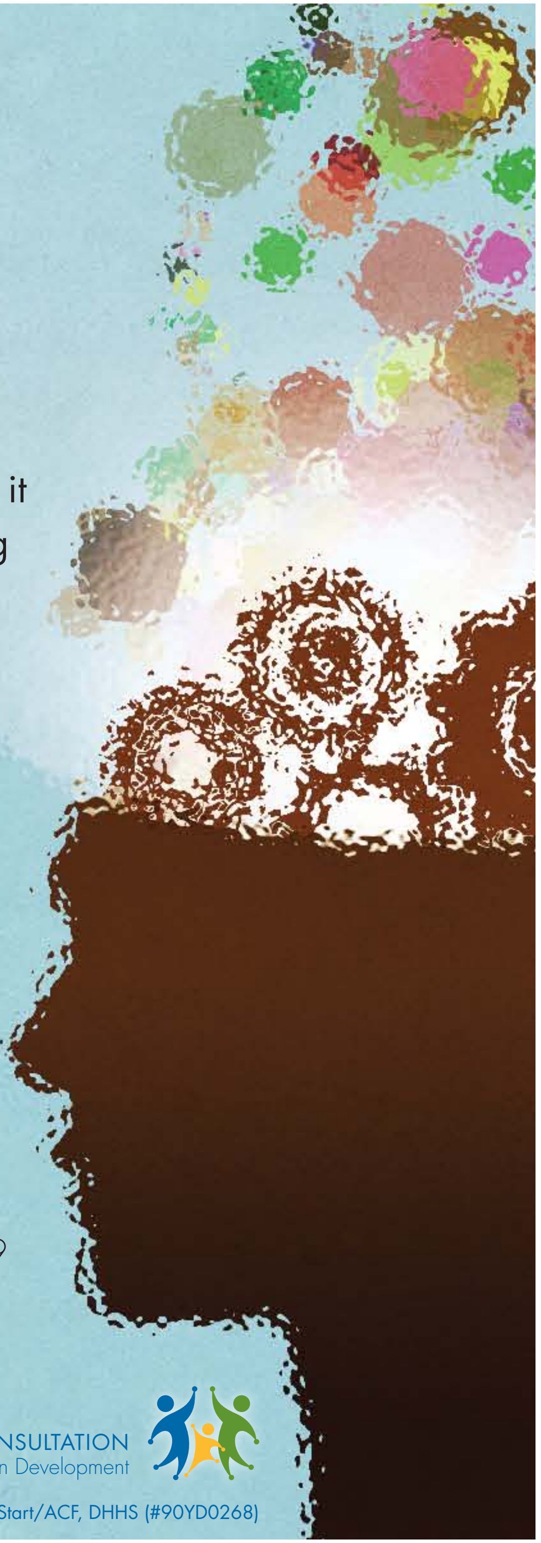

CENTER FOR EARLY CHILDHOOD MENTAL HEALTH CONSULTATION
Georgetown University Center for Child and Human Development

Funded by the Office of Head Start/ACF, DHHS (#90YD0268)

Used with permission from Georgetown University.

STRESS

Talk Back to Your Unhelpful Thoughts II

Stress comes from our perception of the situation. Technically, the actual situation is not stressful; it is our **PERCEPTION** that makes it stressful. Here are some common unhelpful patterns of thinking that we all have as well as ways you can think about challenging these thoughts.

Jumping to Conclusions: You make a negative interpretation even though you don't have all of the facts.

Challenge: Ask yourself, "Do I really know this to be true?" If no, focus on the things that you do know are true and which pieces of information you still need to make a realistic assessment.

Catastrophizing: You negatively exaggerate the importance of things.

Challenge: Try to take the event for what it is and do not let your mind go astray.

Emotional Reasoning: You assume that your negative emotions necessarily reflect the way things really are. "I feel it, therefore it MUST be true."

Challenge: Seek out the opinions of trusted friends or family to evaluate whether your thoughts are accurate.

Should Statements: You try to motivate yourself by saying, "I should or shouldn't" do something.

Challenge: Think about the advantages and disadvantages of your thoughts, feelings and or behaviors. Are you gaining anything from your thoughts or feelings? Discuss with a friend or family member to determine the accuracy of your thoughts.

Personalization: You see yourself as the cause of a negative event for which you were not entirely responsible.

Challenge: Carefully evaluate the situation to figure out if you really have any responsibility for the results. Identify other, outside factors that may be impacting the outcome.

— BURNS, D.D. 1989

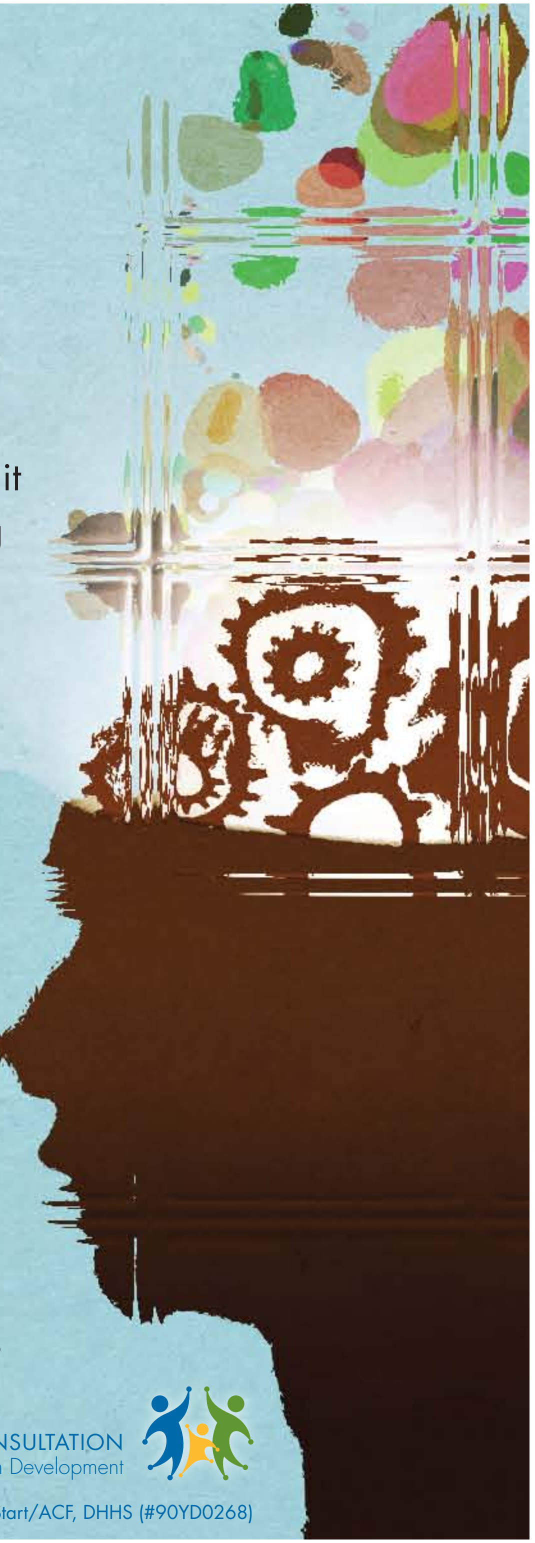

CENTER FOR EARLY CHILDHOOD MENTAL HEALTH CONSULTATION
Georgetown University Center for Child and Human Development

Funded by the Office of Head Start/ACF, DHHS (#90YD0268)

Used with permission from Georgetown University.
