# Supplementary material for: An Interactive Lifestyle Medicine Curriculum for Third-Year Medical Students to Promote Student and Patient Wellness
Source: MedEdPORTAL. 2020 Sep 18;16:10972. doi: 10.15766/mep_2374-8265.10972 (PMC7499809; doi:10.15766/mep_2374-8265.10972)
Supplement: Supplementary file 1 — Introduction & Stress Management Presentation.pptxIntroduction & Stress Management Facilitator Guide.docxUnhealthy Thoughts Handout.pdfGood Things Worksheet.pdfNutrition Presentation.pptxNutrition Facilitator Guide.docxPhysical Activity Presentation.pptxPhysical Activity Facilitator Guide.docxPresession Evaluation.docxPostsession Evaluation.docxSession Evaluation.docx [file mep_2374-8265.10972-s001.zip › E. Nutrition Presentation.pptx]

## Slide 1
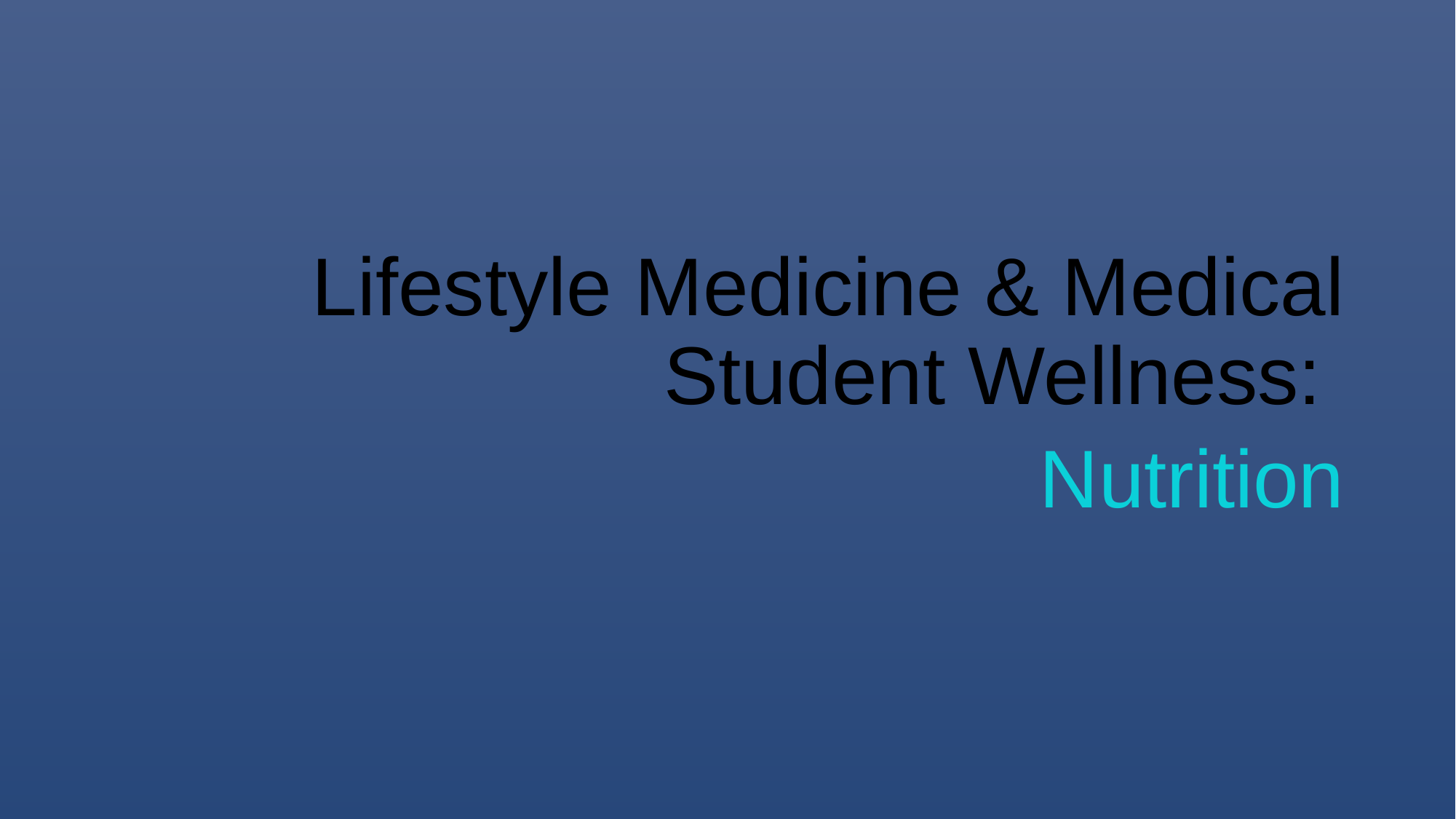

Lifestyle Medicine & Medical Student Wellness:
Nutrition

## Slide 2
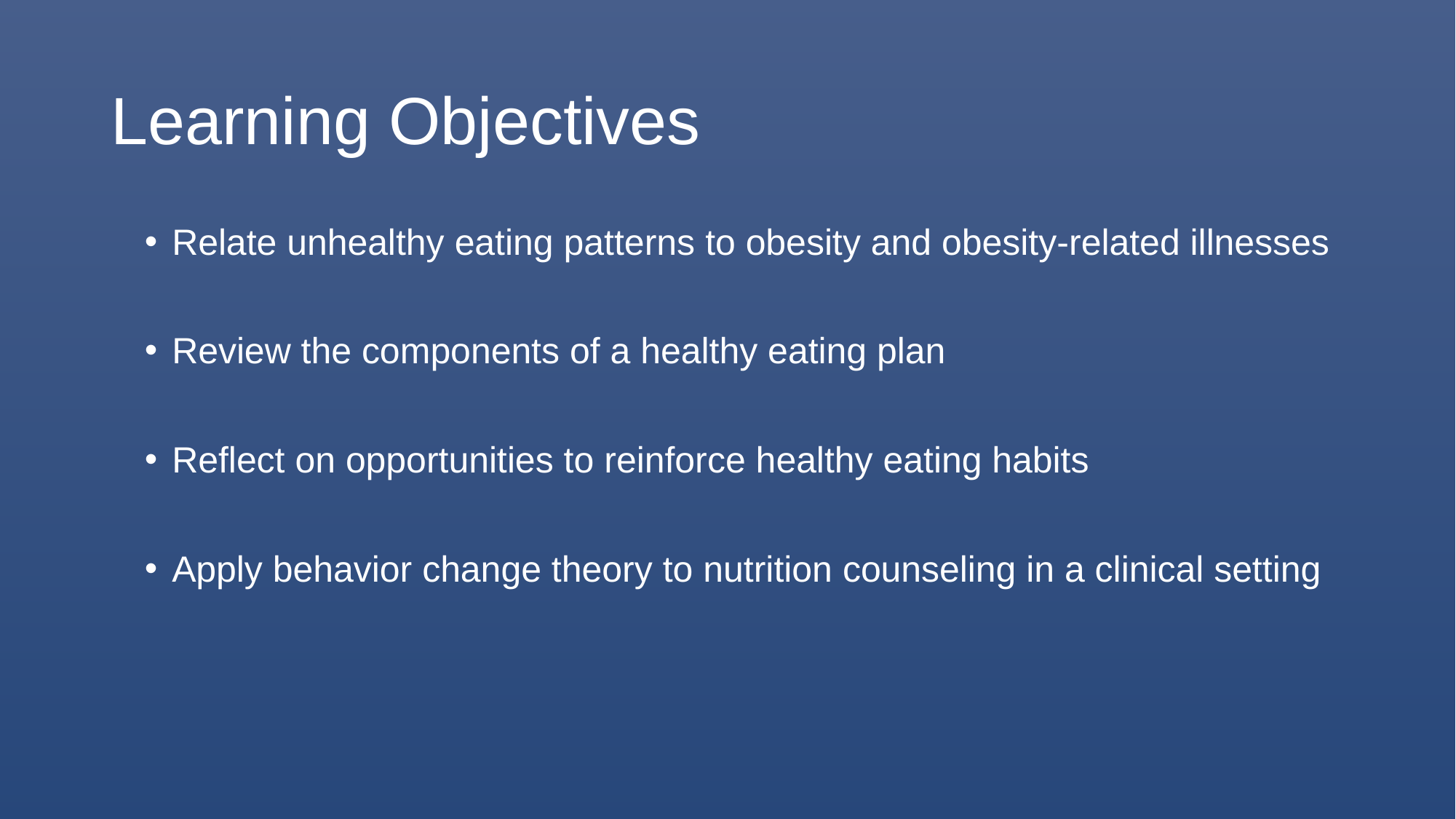

# Learning Objectives
Relate unhealthy eating patterns to obesity and obesity-related illnesses
Review the components of a healthy eating plan
Reflect on opportunities to reinforce healthy eating habits
Apply behavior change theory to nutrition counseling in a clinical setting

## Slide 3
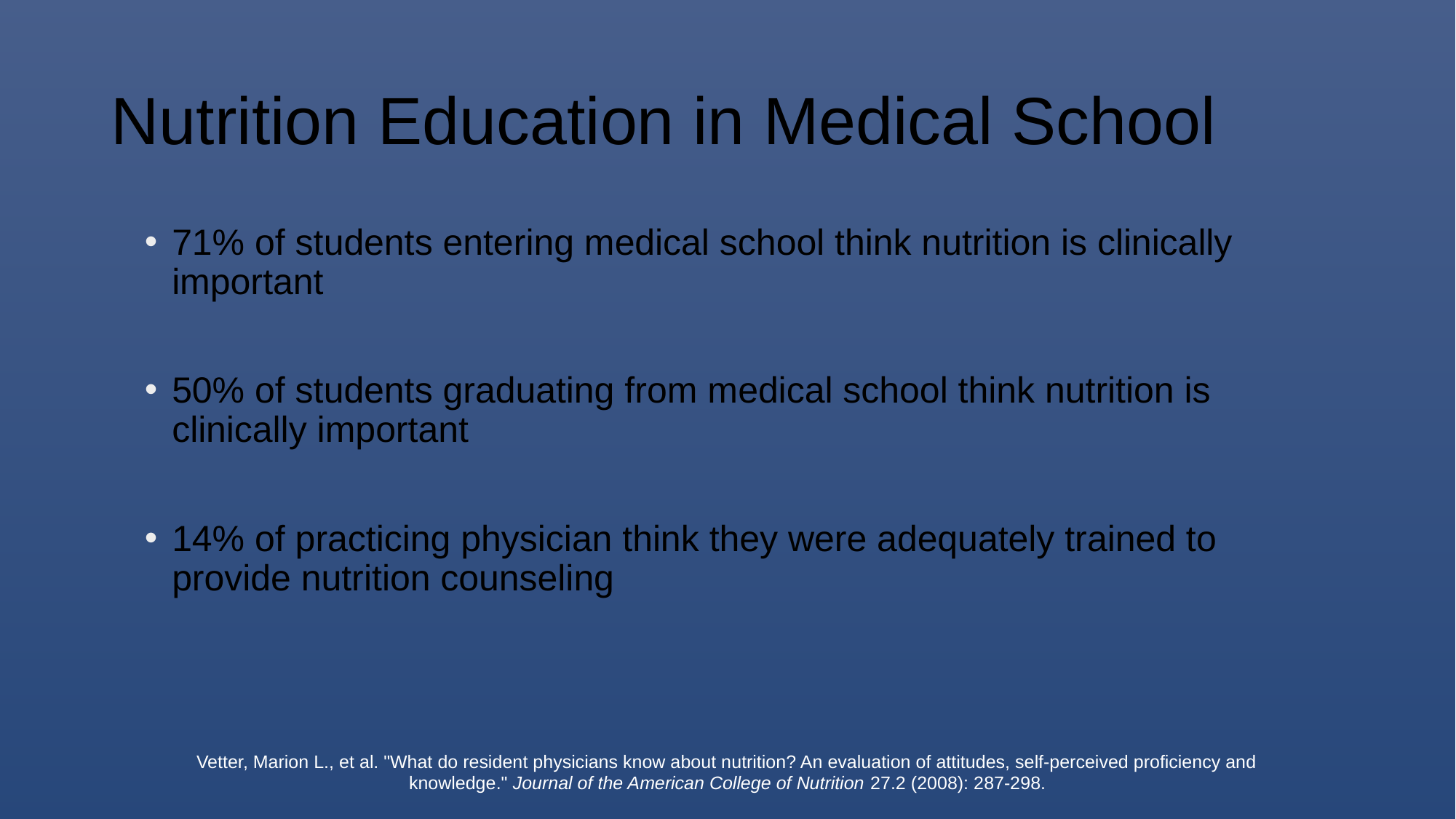

# Nutrition Education in Medical School
71% of students entering medical school think nutrition is clinically important
50% of students graduating from medical school think nutrition is clinically important
14% of practicing physician think they were adequately trained to provide nutrition counseling
Vetter, Marion L., et al. "What do resident physicians know about nutrition? An evaluation of attitudes, self-perceived proficiency and knowledge." Journal of the American College of Nutrition 27.2 (2008): 287-298.

## Slide 4
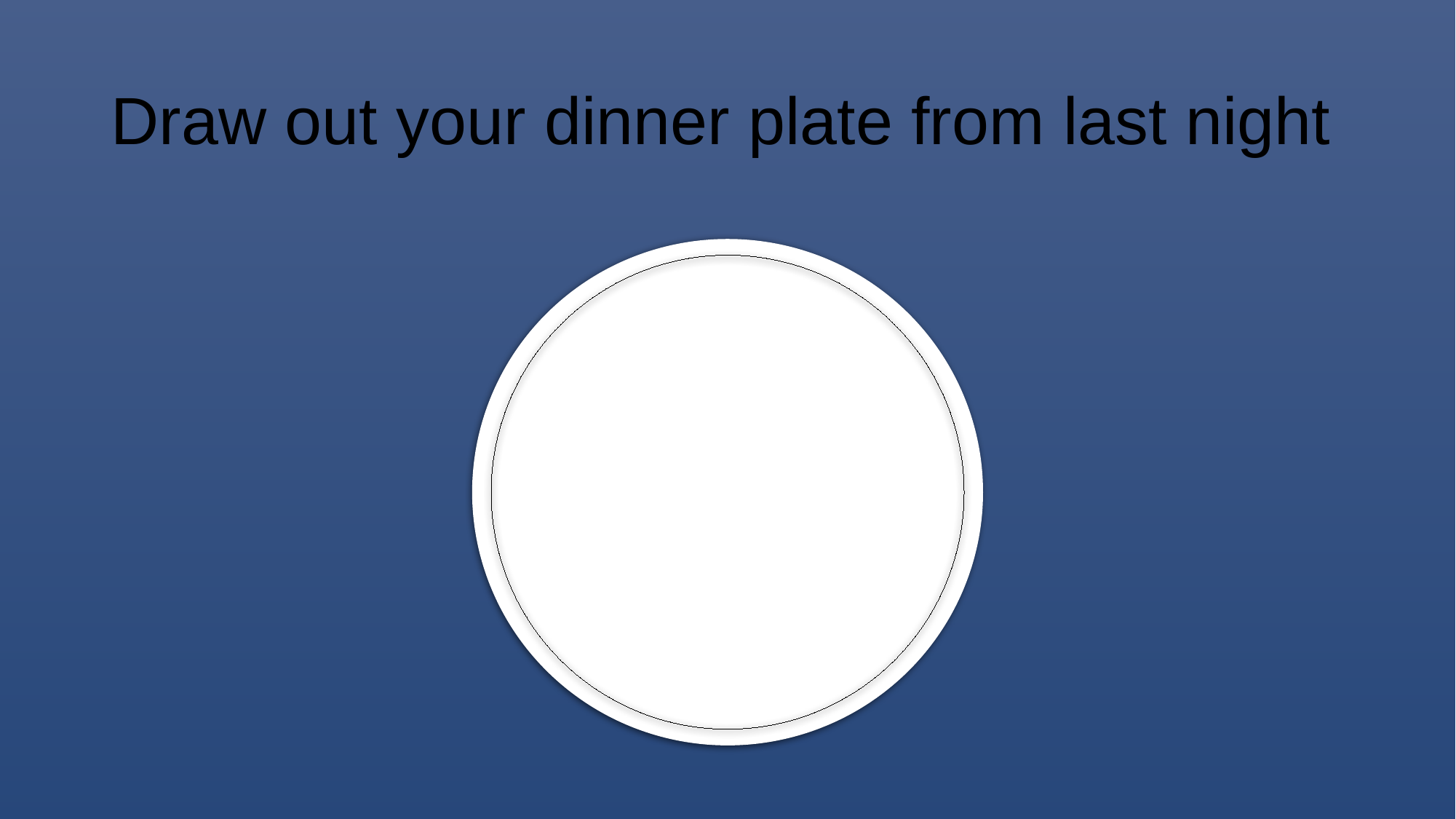

# Draw out your dinner plate from last night

## Slide 5
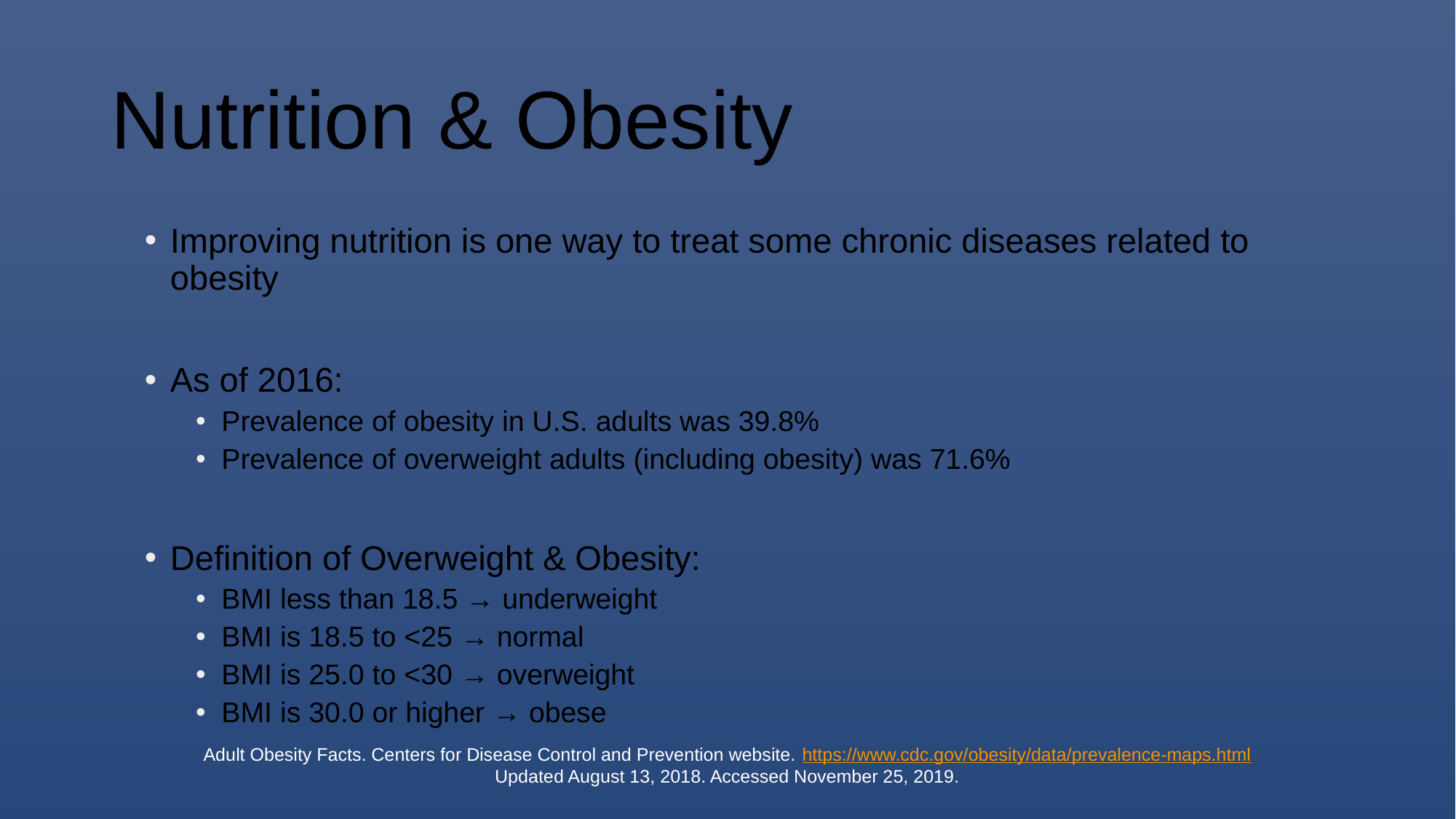

# Nutrition & Obesity
Improving nutrition is one way to treat some chronic diseases related to obesity
As of 2016:
Prevalence of obesity in U.S. adults was 39.8%
Prevalence of overweight adults (including obesity) was 71.6%
Definition of Overweight & Obesity:
BMI less than 18.5 → underweight
BMI is 18.5 to <25 → normal
BMI is 25.0 to <30 → overweight
BMI is 30.0 or higher → obese
Adult Obesity Facts. Centers for Disease Control and Prevention website. https://www.cdc.gov/obesity/data/prevalence-maps.html
Updated August 13, 2018. Accessed November 25, 2019.

## Slide 6
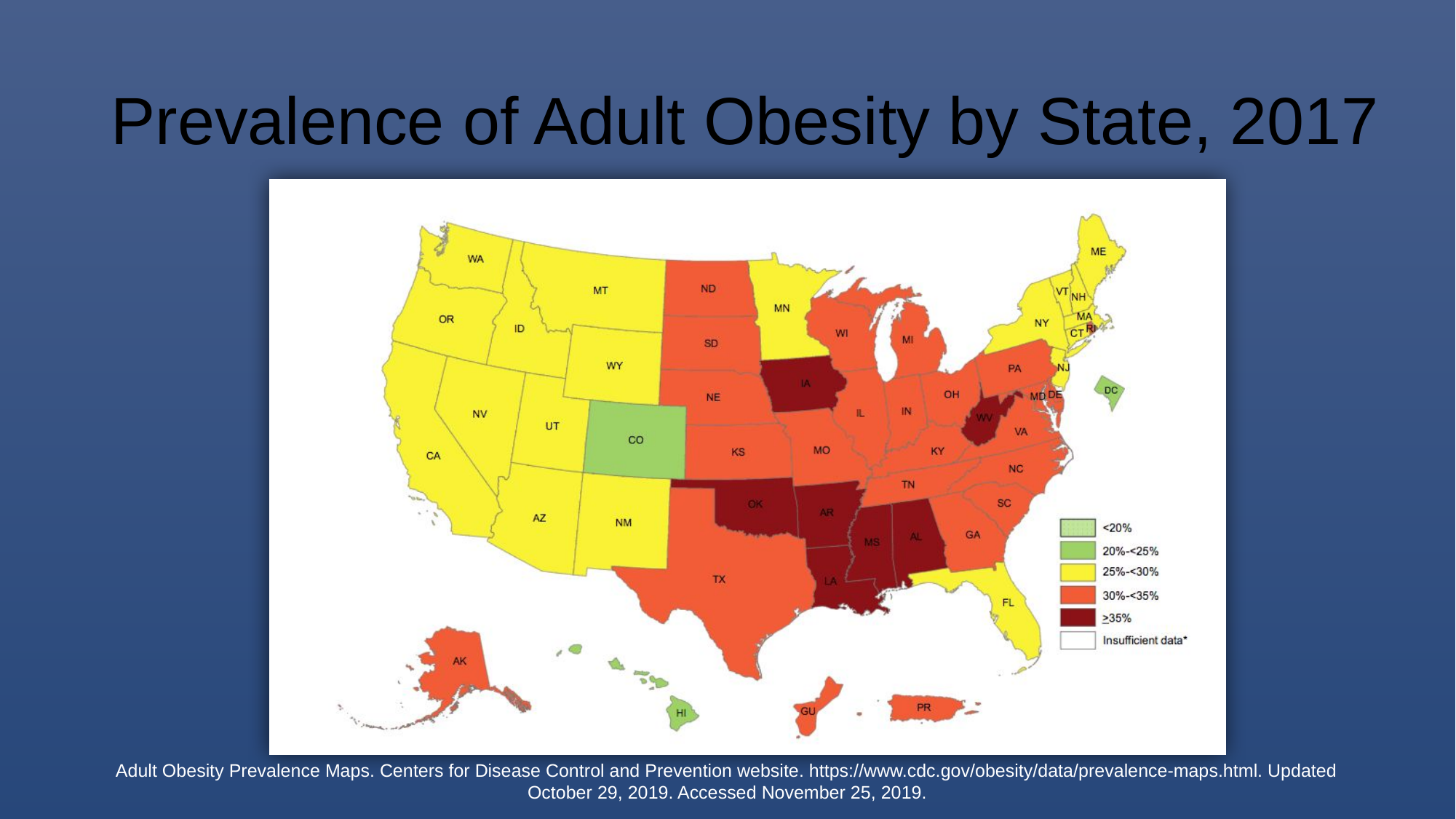

# Prevalence of Adult Obesity by State, 2017
Adult Obesity Prevalence Maps. Centers for Disease Control and Prevention website. https://www.cdc.gov/obesity/data/prevalence-maps.html. Updated October 29, 2019. Accessed November 25, 2019.

## Slide 7
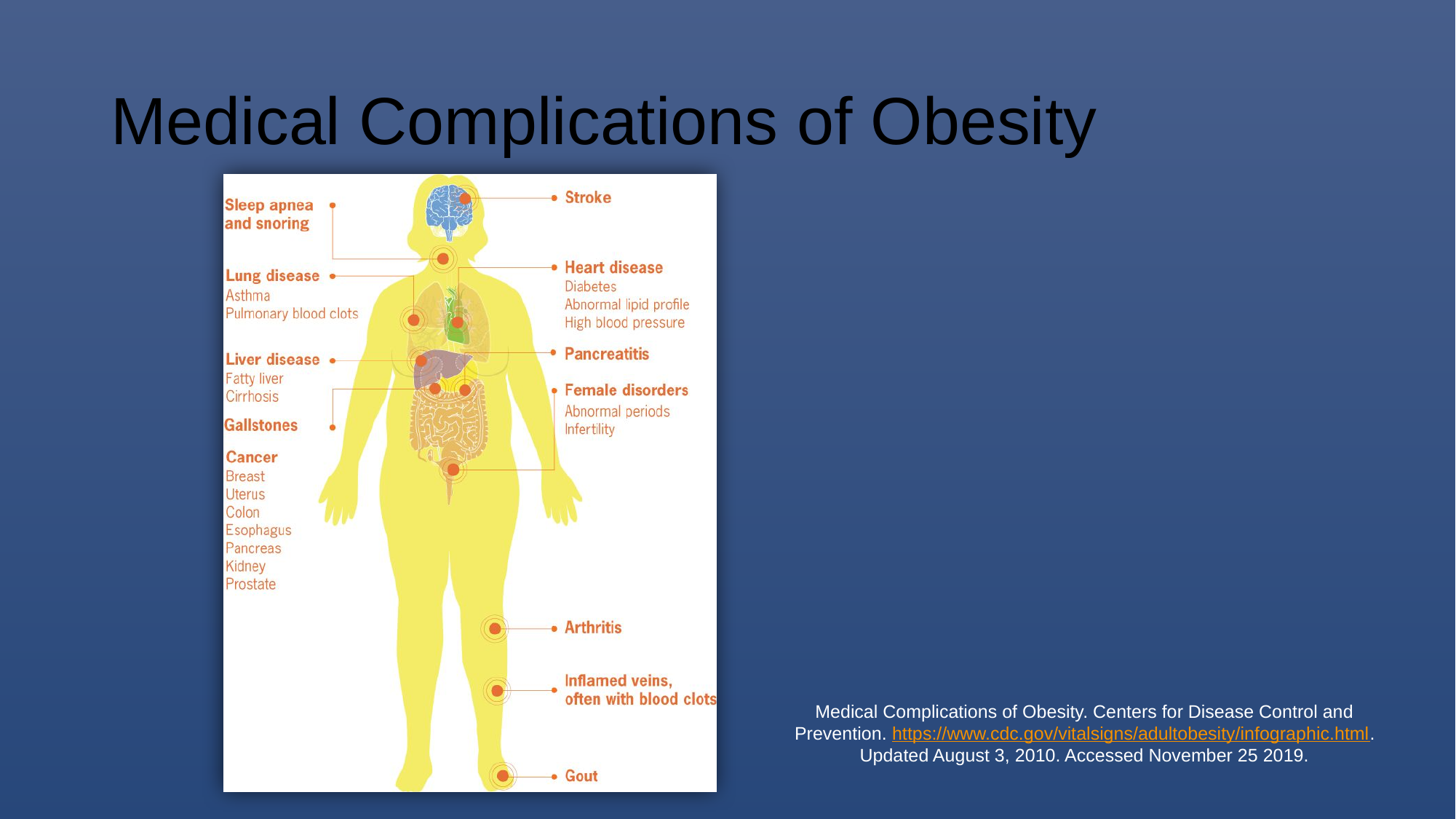

# Medical Complications of Obesity
Medical Complications of Obesity. Centers for Disease Control and Prevention. https://www.cdc.gov/vitalsigns/adultobesity/infographic.html. Updated August 3, 2010. Accessed November 25 2019.

## Slide 8
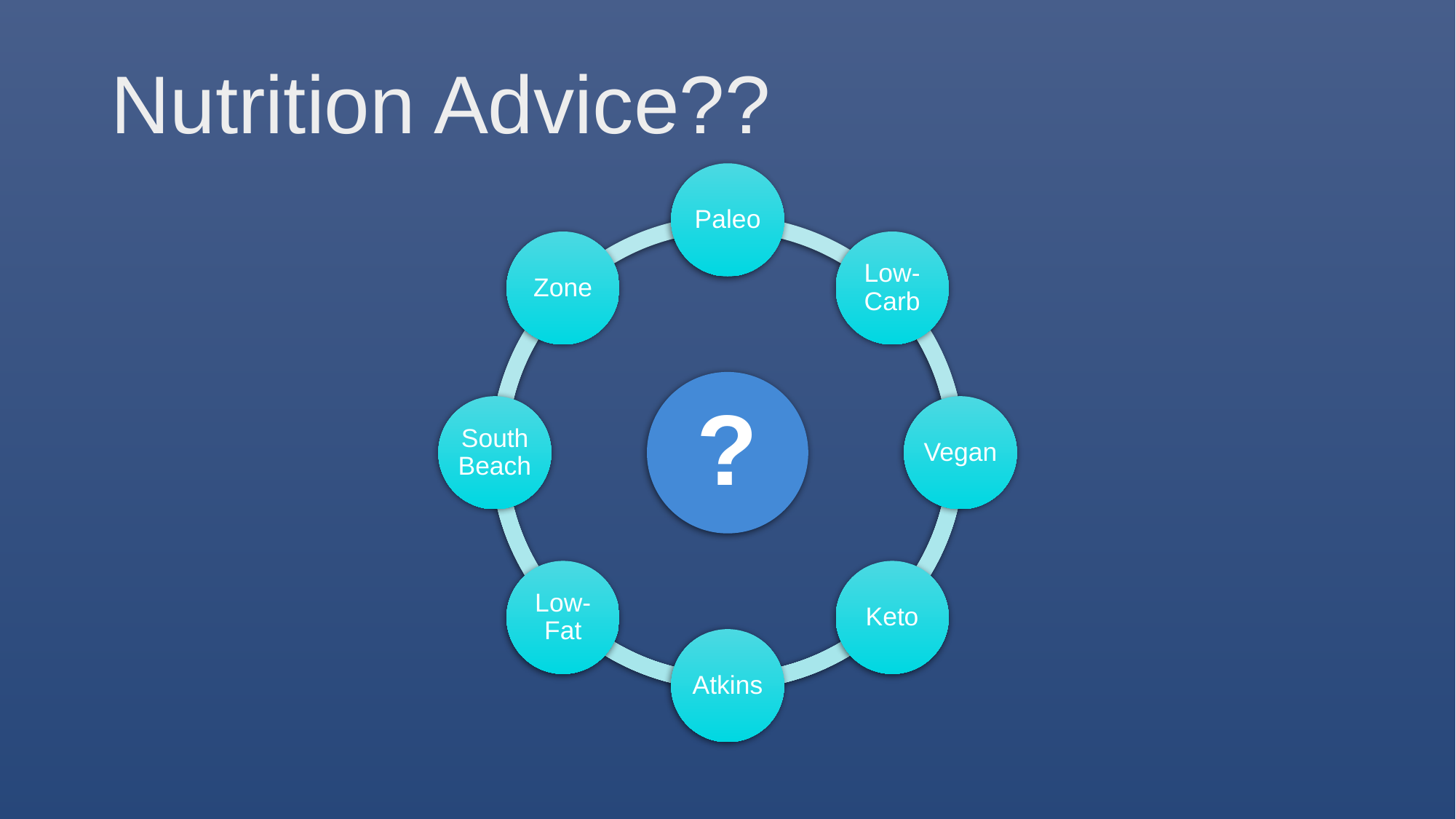

Nutrition Advice??

## Slide 9
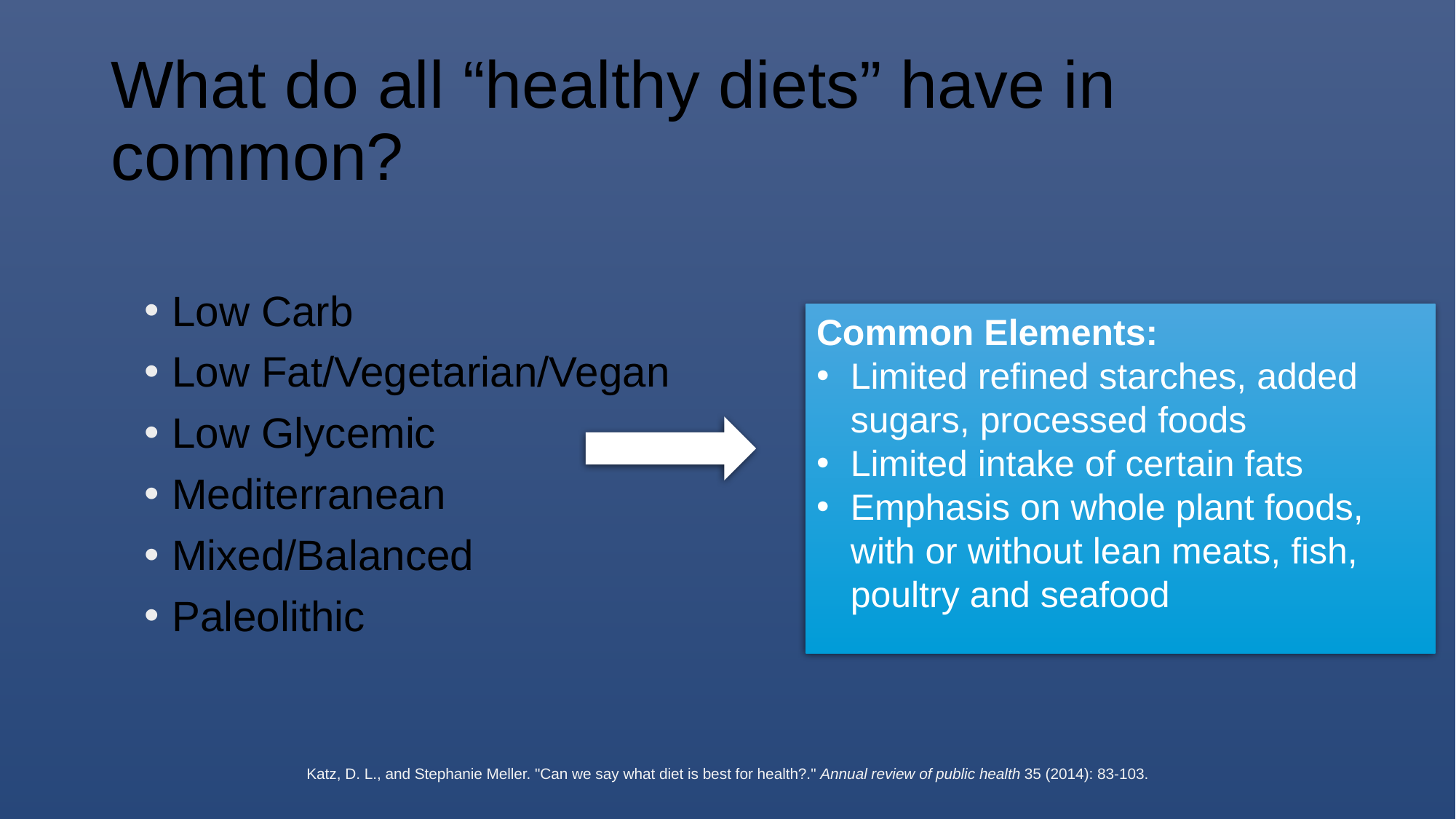

# What do all “healthy diets” have in common?
Low Carb
Low Fat/Vegetarian/Vegan
Low Glycemic
Mediterranean
Mixed/Balanced
Paleolithic
Common Elements:
Limited refined starches, added sugars, processed foods
Limited intake of certain fats
Emphasis on whole plant foods, with or without lean meats, fish, poultry and seafood
Katz, D. L., and Stephanie Meller. "Can we say what diet is best for health?." Annual review of public health 35 (2014): 83-103.

## Slide 10
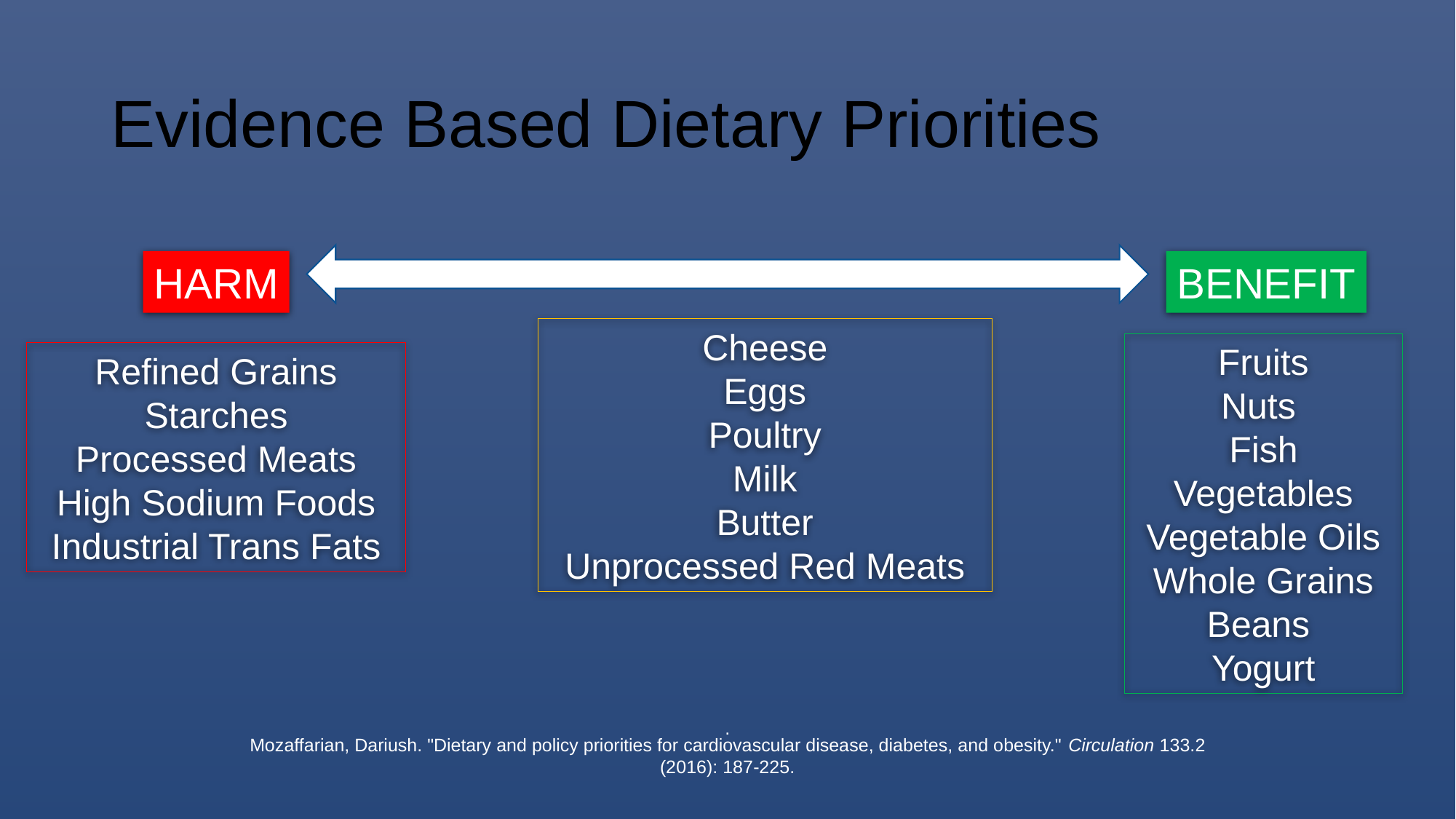

# Evidence Based Dietary Priorities
HARM
BENEFIT
Cheese
Eggs
Poultry
Milk
Butter
Unprocessed Red Meats
Fruits
Nuts
Fish
Vegetables
Vegetable Oils
Whole Grains
Beans
Yogurt
Refined Grains
Starches
Processed Meats
High Sodium Foods
Industrial Trans Fats
.
Mozaffarian, Dariush. "Dietary and policy priorities for cardiovascular disease, diabetes, and obesity." Circulation 133.2 (2016): 187-225.

## Slide 11
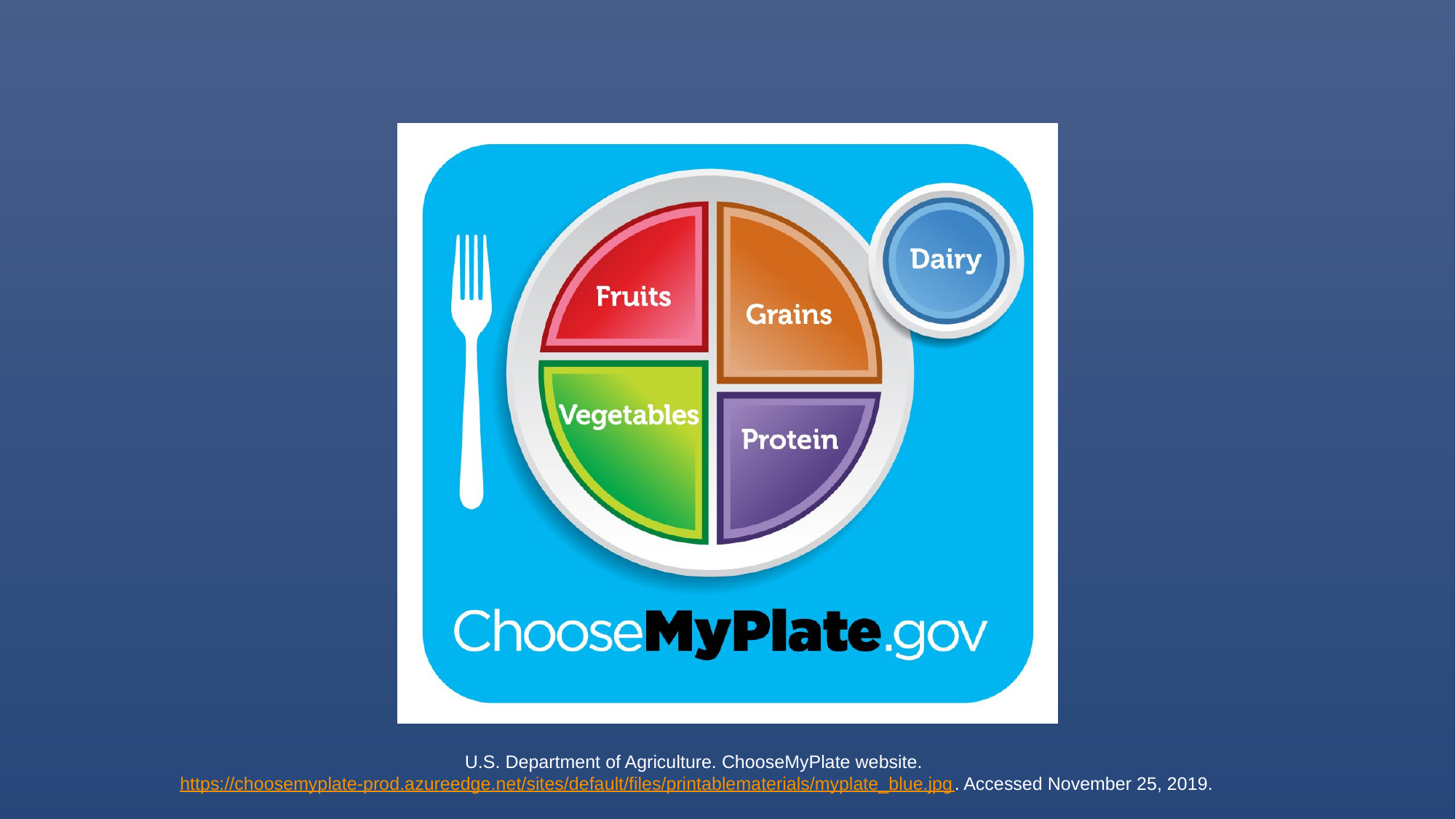

U.S. Department of Agriculture. ChooseMyPlate website. https://choosemyplate-prod.azureedge.net/sites/default/files/printablematerials/myplate_blue.jpg. Accessed November 25, 2019.

## Slide 12
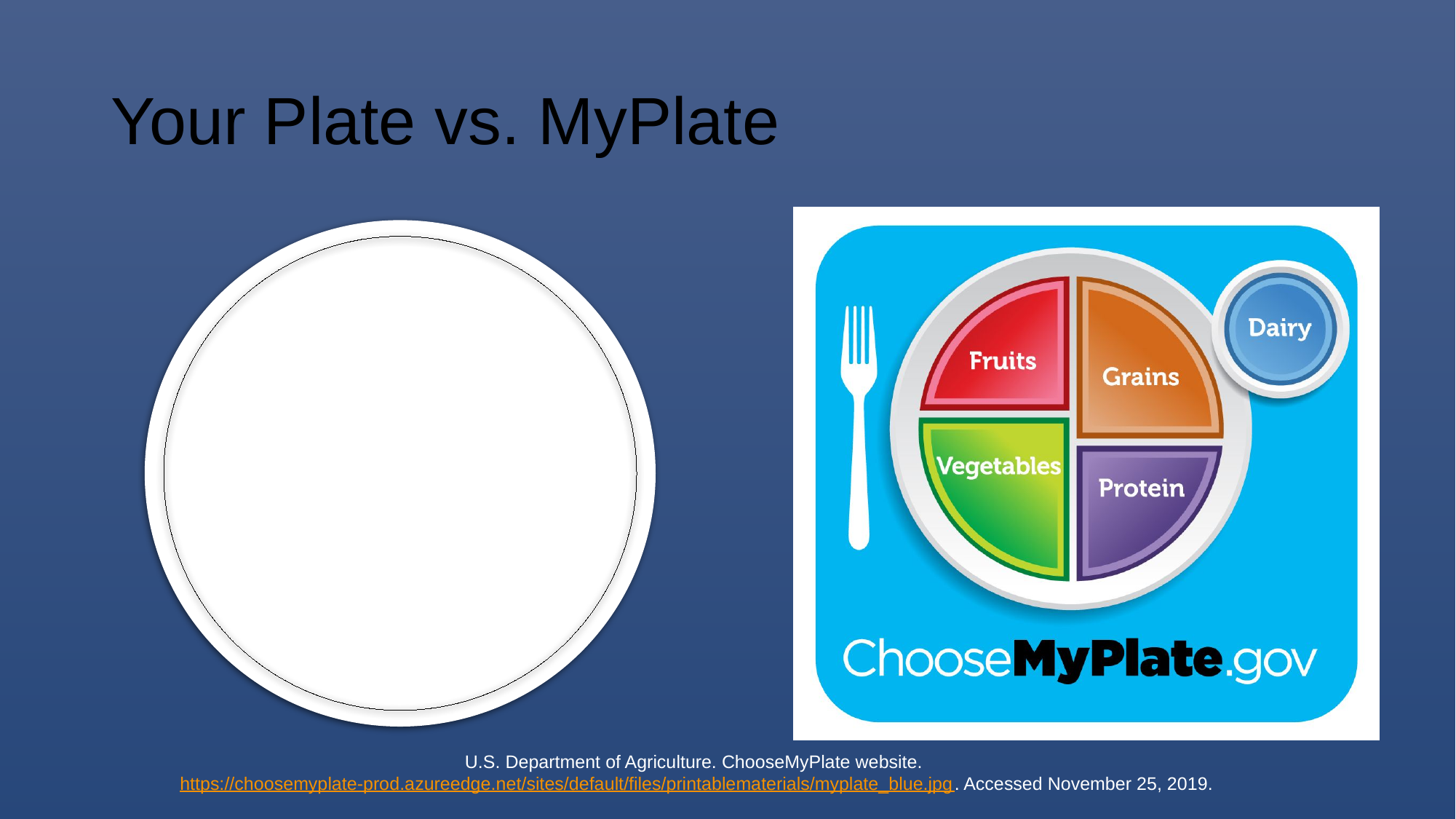

# Your Plate vs. MyPlate
U.S. Department of Agriculture. ChooseMyPlate website. https://choosemyplate-prod.azureedge.net/sites/default/files/printablematerials/myplate_blue.jpg. Accessed November 25, 2019.

## Slide 13
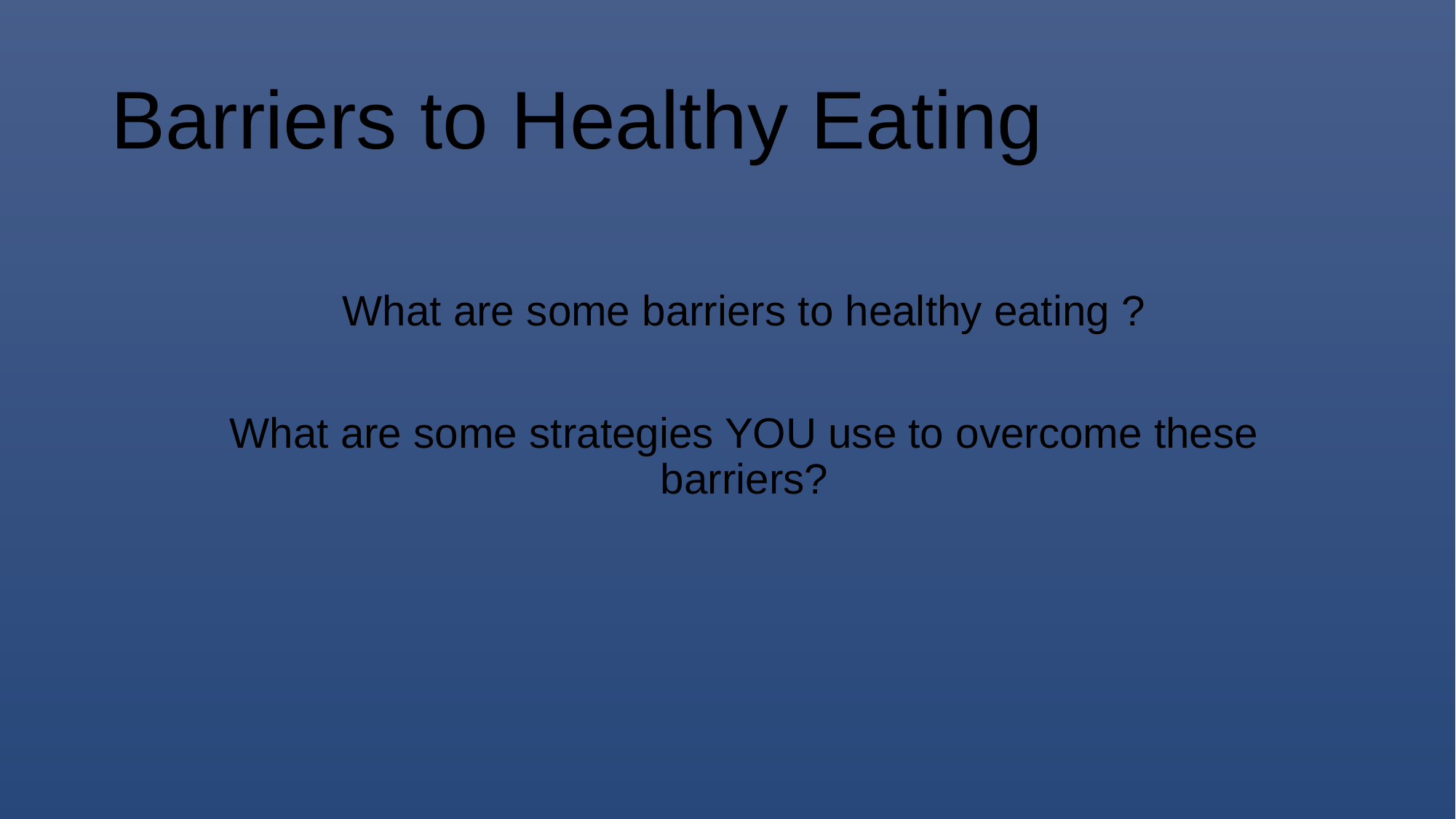

# Barriers to Healthy Eating
What are some barriers to healthy eating ?
What are some strategies YOU use to overcome these barriers?

## Slide 14
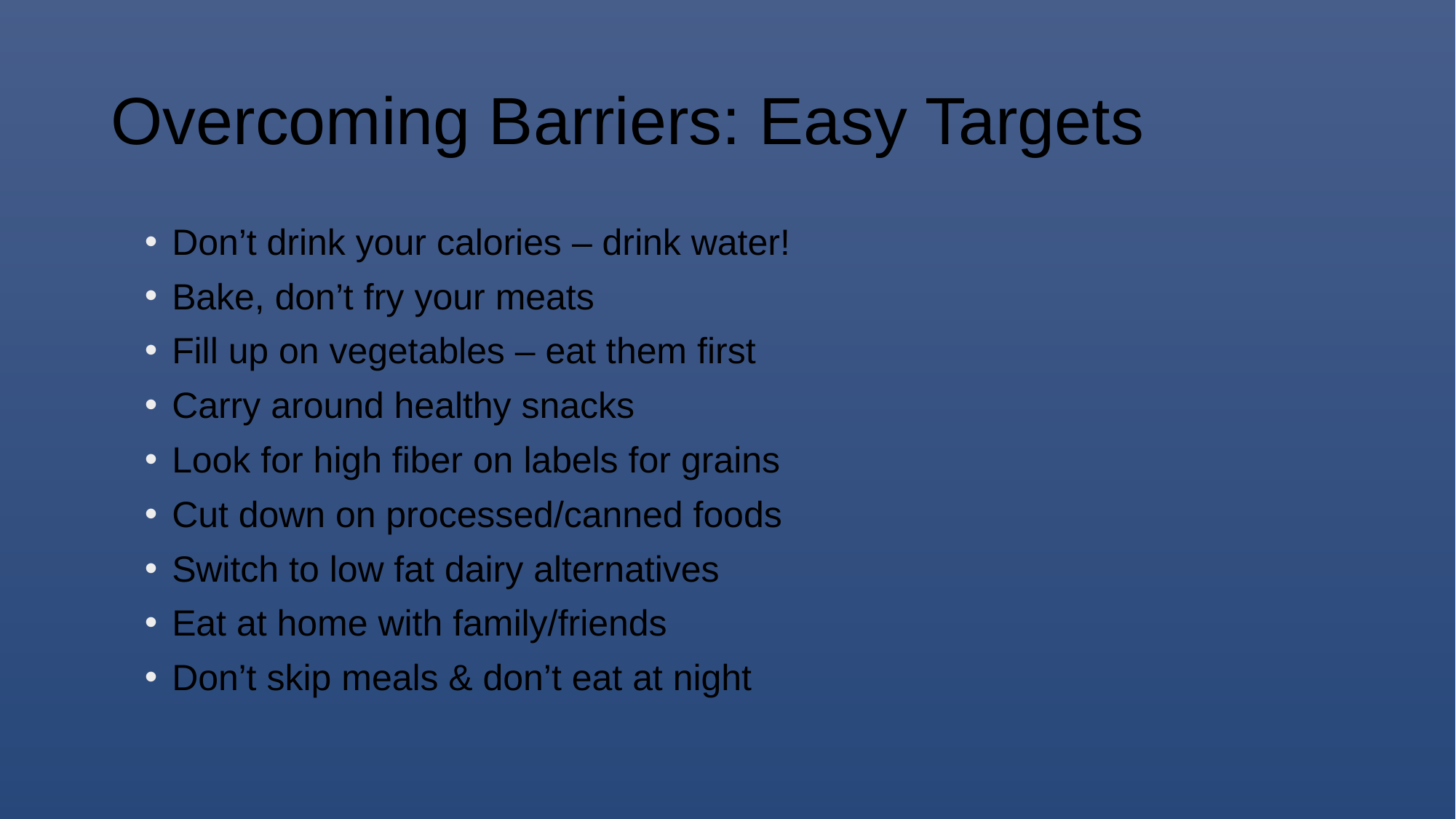

# Overcoming Barriers: Easy Targets
Don’t drink your calories – drink water!
Bake, don’t fry your meats
Fill up on vegetables – eat them first
Carry around healthy snacks
Look for high fiber on labels for grains
Cut down on processed/canned foods
Switch to low fat dairy alternatives
Eat at home with family/friends
Don’t skip meals & don’t eat at night

## Slide 15
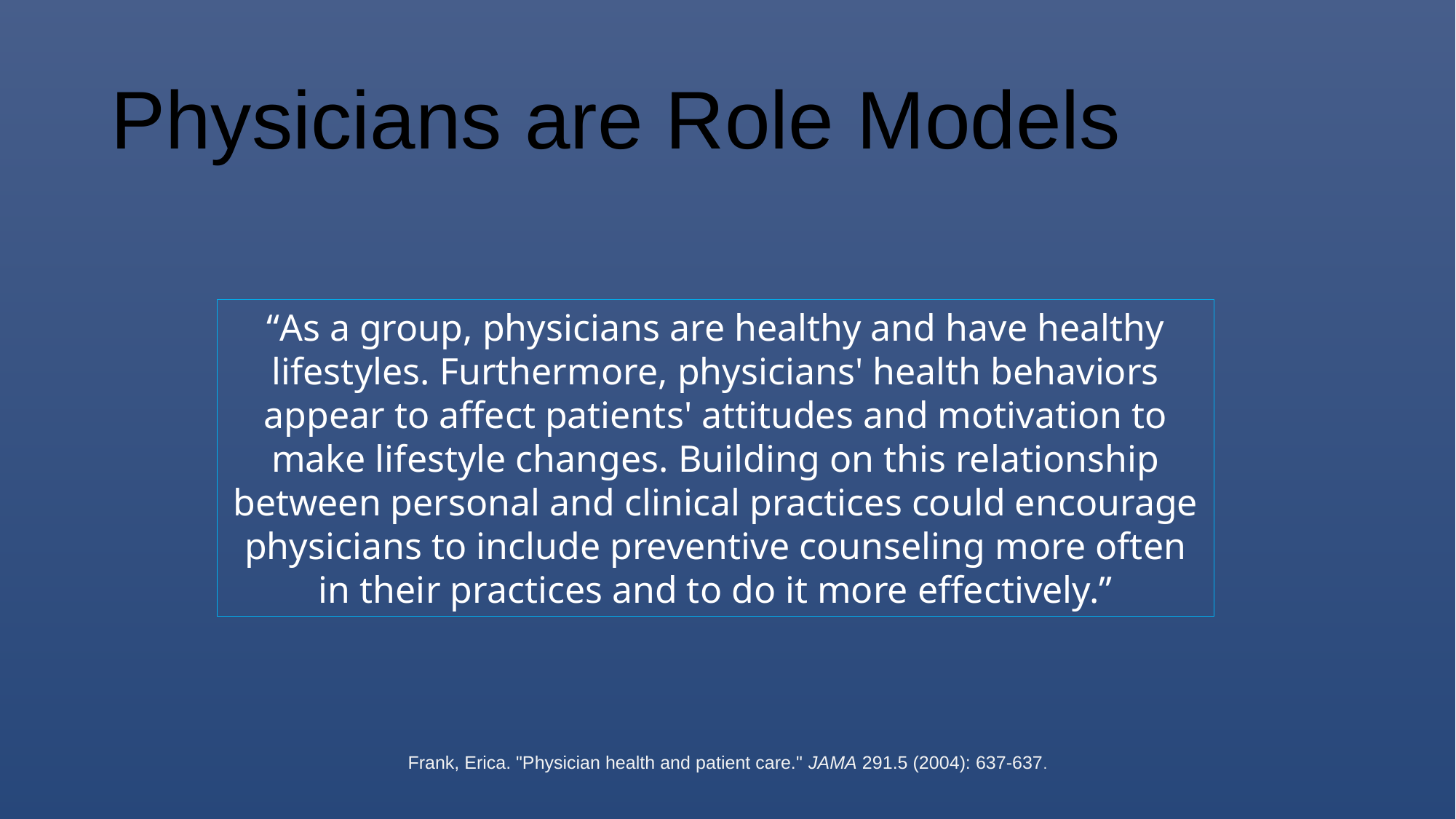

# Physicians are Role Models
“As a group, physicians are healthy and have healthy lifestyles. Furthermore, physicians' health behaviors appear to affect patients' attitudes and motivation to make lifestyle changes. Building on this relationship between personal and clinical practices could encourage physicians to include preventive counseling more often in their practices and to do it more effectively.”
Frank, Erica. "Physician health and patient care." JAMA 291.5 (2004): 637-637.

## Slide 16
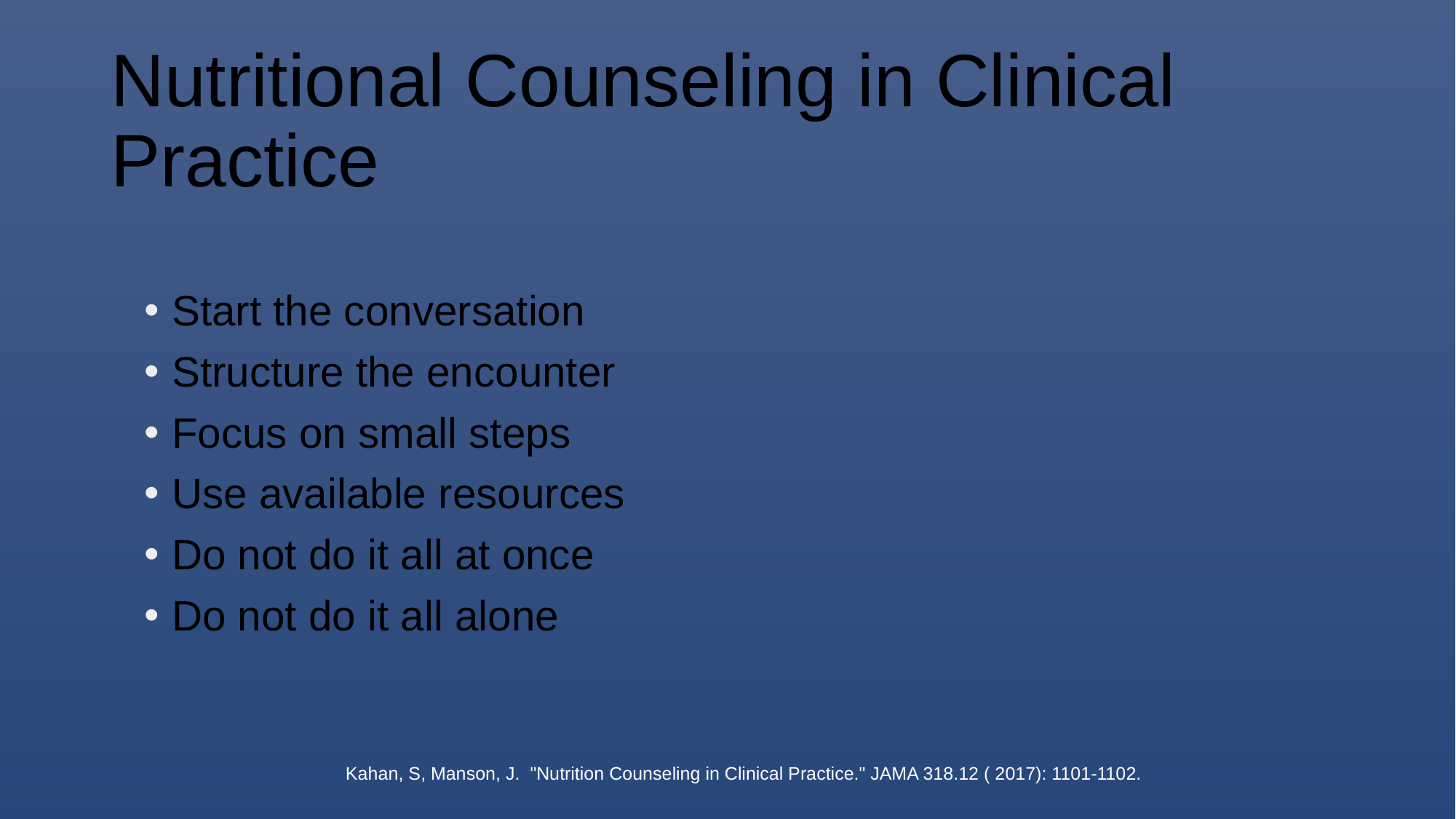

# Nutritional Counseling in Clinical Practice
Start the conversation
Structure the encounter
Focus on small steps
Use available resources
Do not do it all at once
Do not do it all alone
Kahan, S, Manson, J.  "Nutrition Counseling in Clinical Practice." JAMA 318.12 ( 2017): 1101-1102.

## Slide 17
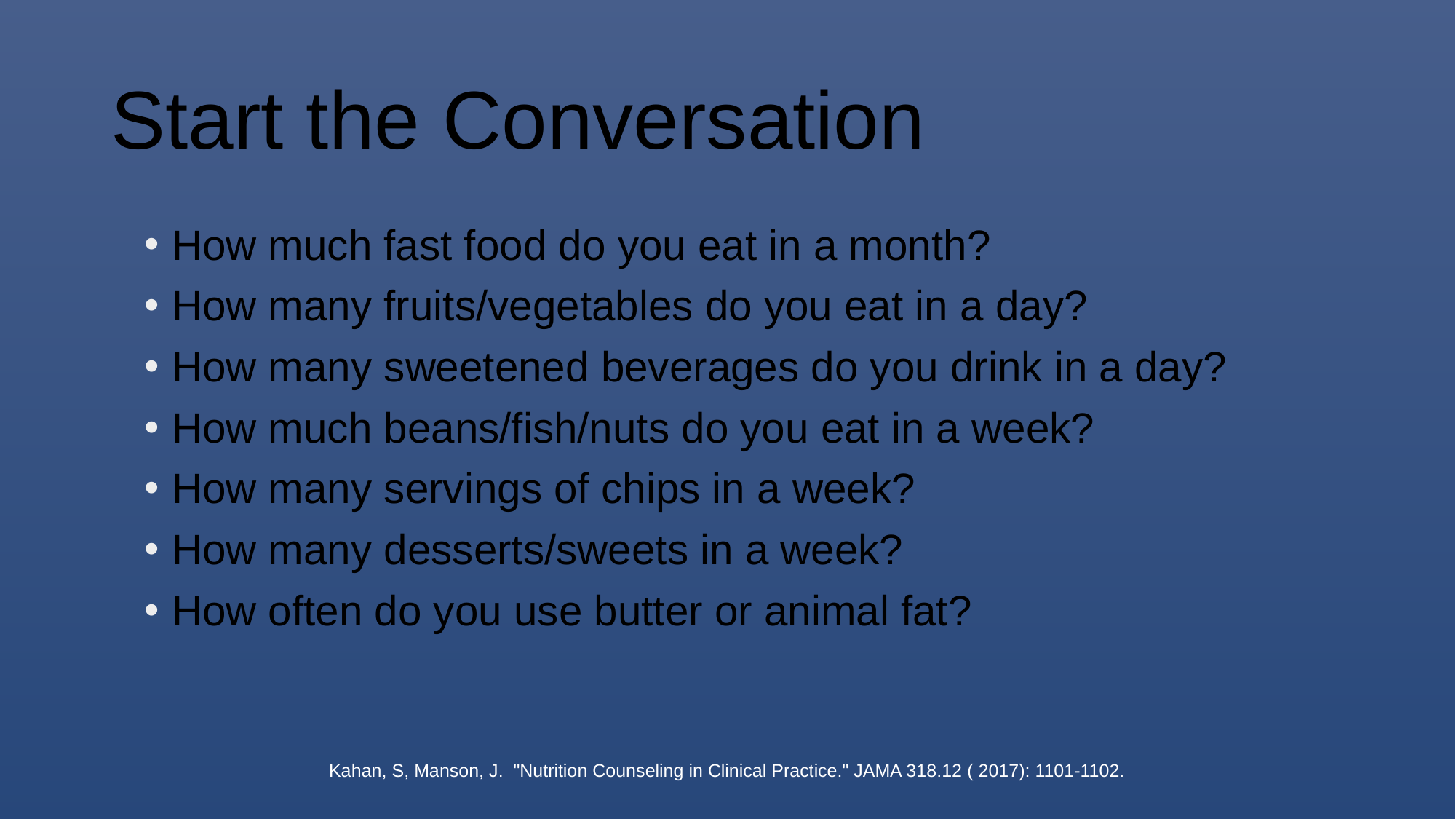

# Start the Conversation
How much fast food do you eat in a month?
How many fruits/vegetables do you eat in a day?
How many sweetened beverages do you drink in a day?
How much beans/fish/nuts do you eat in a week?
How many servings of chips in a week?
How many desserts/sweets in a week?
How often do you use butter or animal fat?
Kahan, S, Manson, J.  "Nutrition Counseling in Clinical Practice." JAMA 318.12 ( 2017): 1101-1102.

## Slide 18
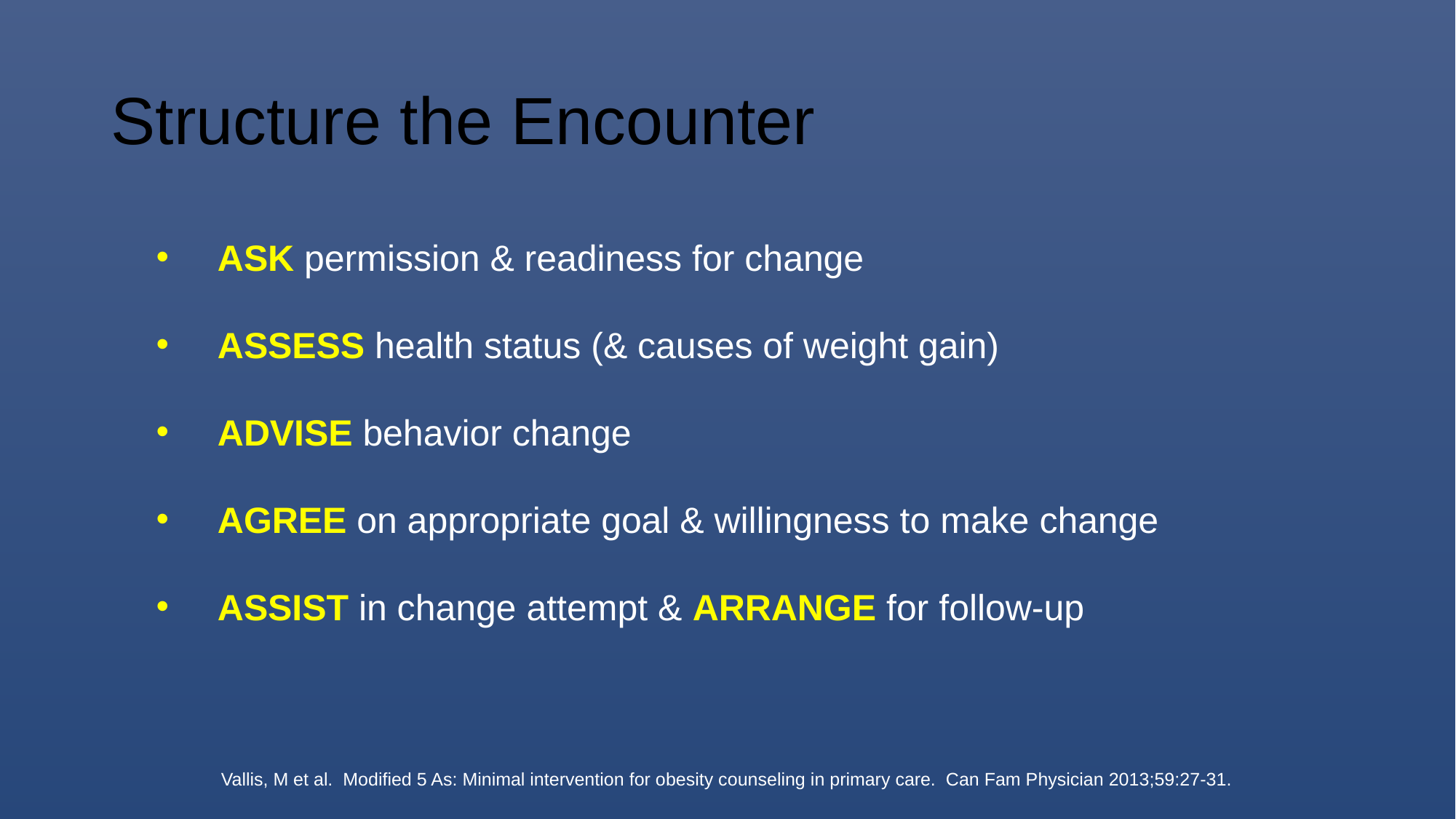

# Structure the Encounter
ASK permission & readiness for change
ASSESS health status (& causes of weight gain)
ADVISE behavior change
AGREE on appropriate goal & willingness to make change
ASSIST in change attempt & ARRANGE for follow-up
Vallis, M et al.  Modified 5 As: Minimal intervention for obesity counseling in primary care.  Can Fam Physician 2013;59:27-31.

## Slide 19
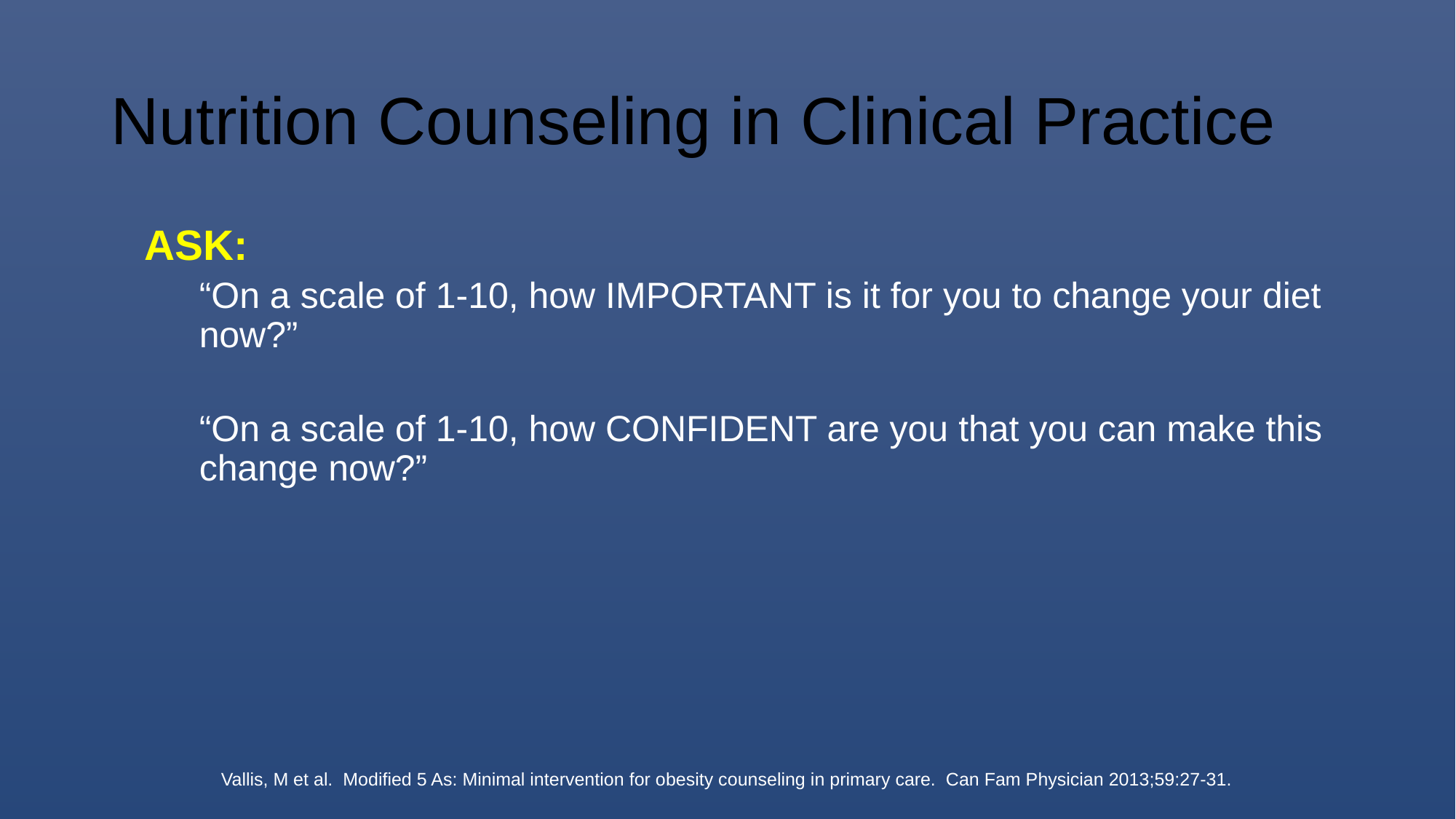

# Nutrition Counseling in Clinical Practice
ASK:
“On a scale of 1-10, how IMPORTANT is it for you to change your diet now?”
“On a scale of 1-10, how CONFIDENT are you that you can make this change now?”
Vallis, M et al.  Modified 5 As: Minimal intervention for obesity counseling in primary care.  Can Fam Physician 2013;59:27-31.

## Slide 20
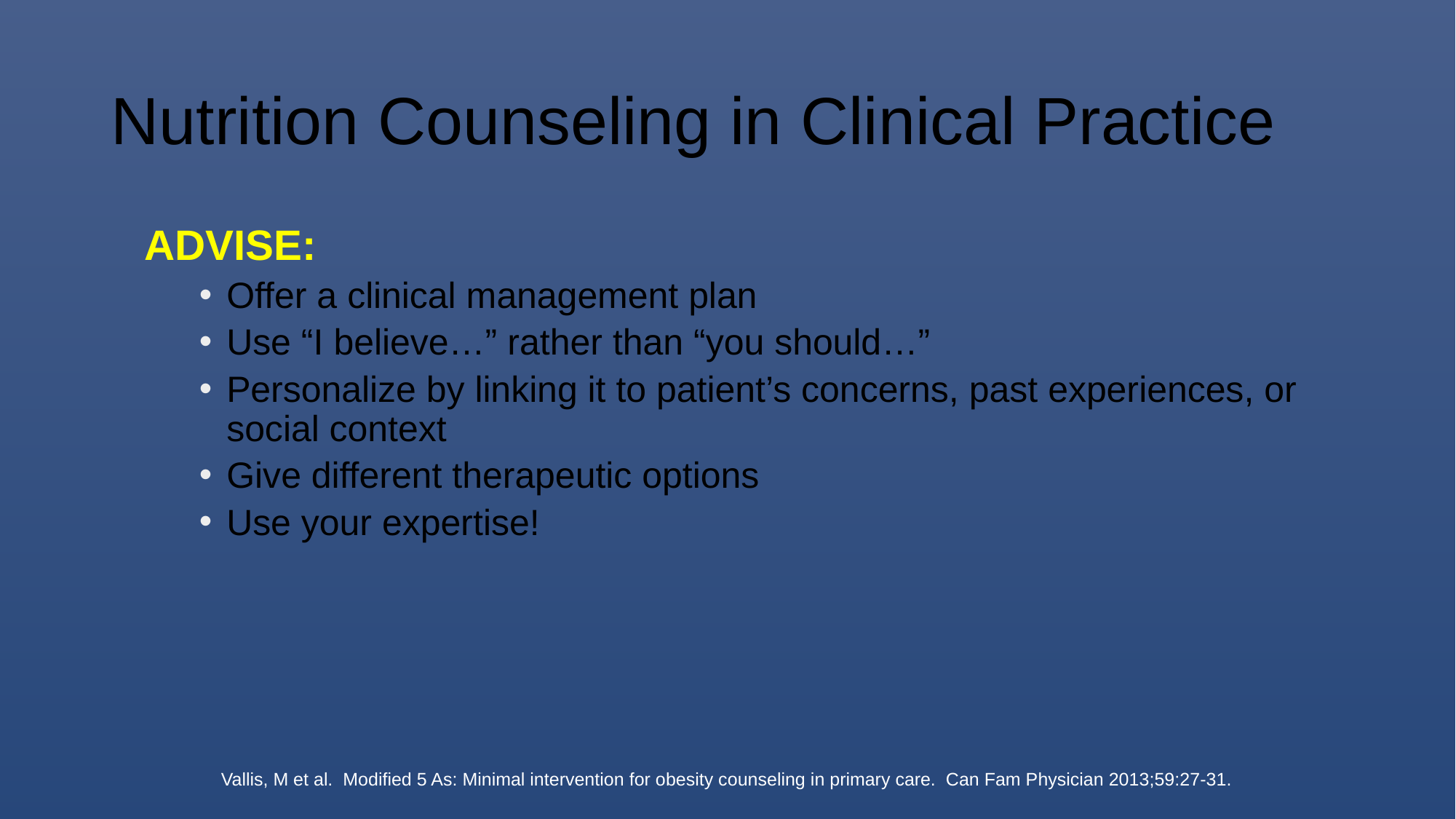

# Nutrition Counseling in Clinical Practice
ADVISE:
Offer a clinical management plan
Use “I believe…” rather than “you should…”
Personalize by linking it to patient’s concerns, past experiences, or social context
Give different therapeutic options
Use your expertise!
Vallis, M et al.  Modified 5 As: Minimal intervention for obesity counseling in primary care.  Can Fam Physician 2013;59:27-31.

## Slide 21
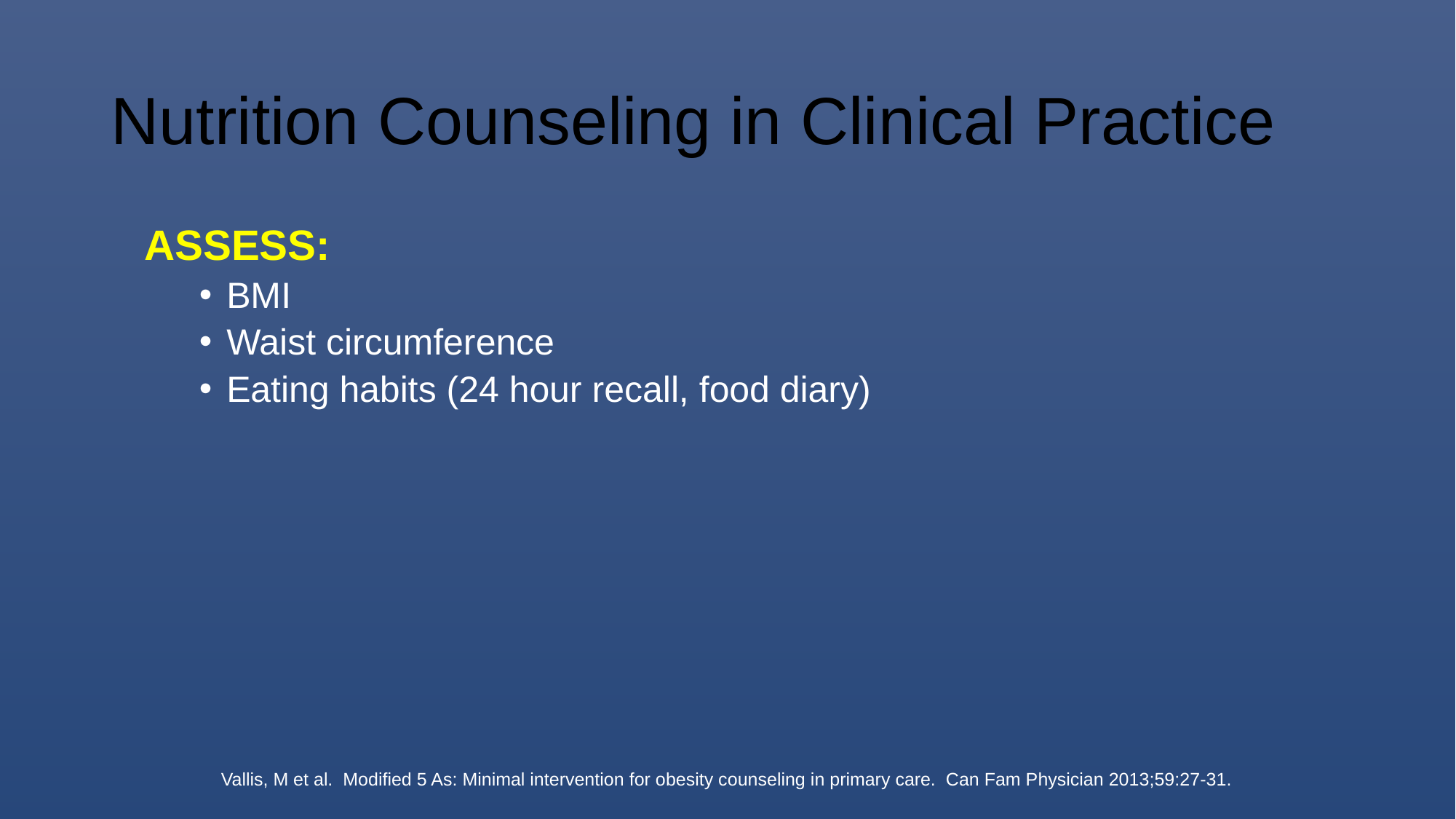

# Nutrition Counseling in Clinical Practice
ASSESS:
BMI
Waist circumference
Eating habits (24 hour recall, food diary)
Vallis, M et al.  Modified 5 As: Minimal intervention for obesity counseling in primary care.  Can Fam Physician 2013;59:27-31.

## Slide 22
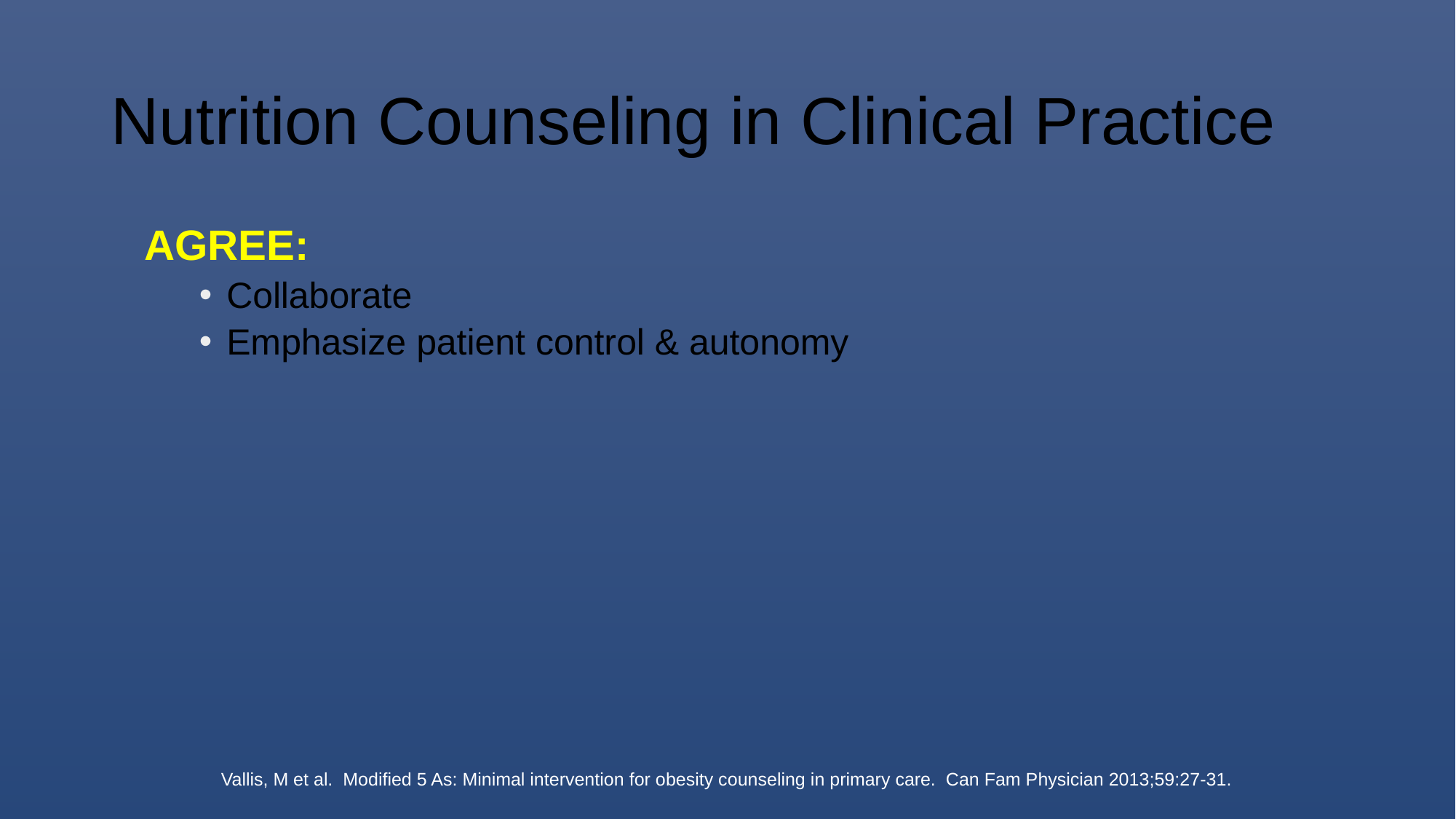

# Nutrition Counseling in Clinical Practice
AGREE:
Collaborate
Emphasize patient control & autonomy
Vallis, M et al.  Modified 5 As: Minimal intervention for obesity counseling in primary care.  Can Fam Physician 2013;59:27-31.

## Slide 23
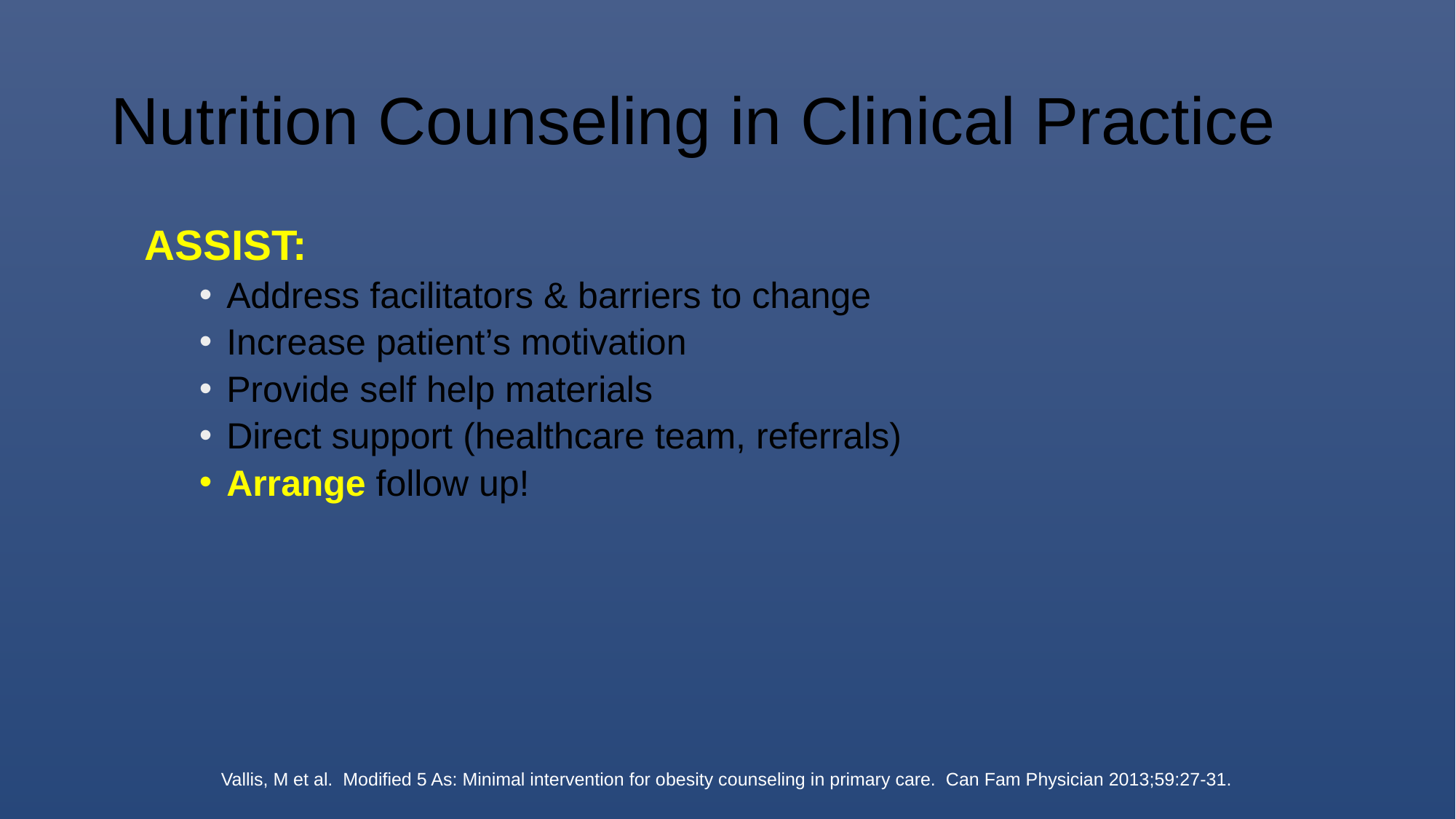

# Nutrition Counseling in Clinical Practice
ASSIST:
Address facilitators & barriers to change
Increase patient’s motivation
Provide self help materials
Direct support (healthcare team, referrals)
Arrange follow up!
Vallis, M et al.  Modified 5 As: Minimal intervention for obesity counseling in primary care.  Can Fam Physician 2013;59:27-31.

## Slide 24
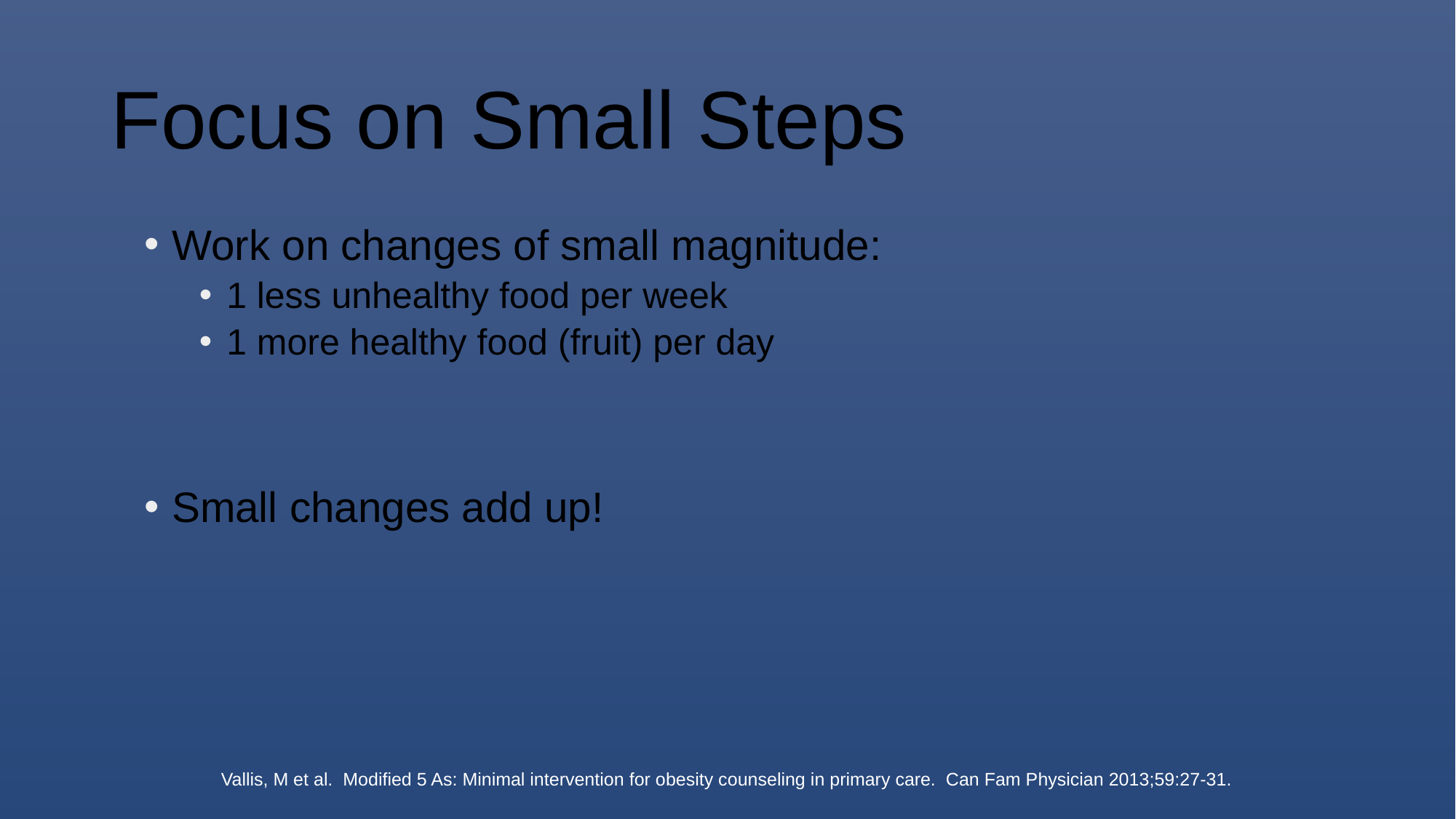

# Focus on Small Steps
Work on changes of small magnitude:
1 less unhealthy food per week
1 more healthy food (fruit) per day
Small changes add up!
Vallis, M et al.  Modified 5 As: Minimal intervention for obesity counseling in primary care.  Can Fam Physician 2013;59:27-31.

## Slide 25
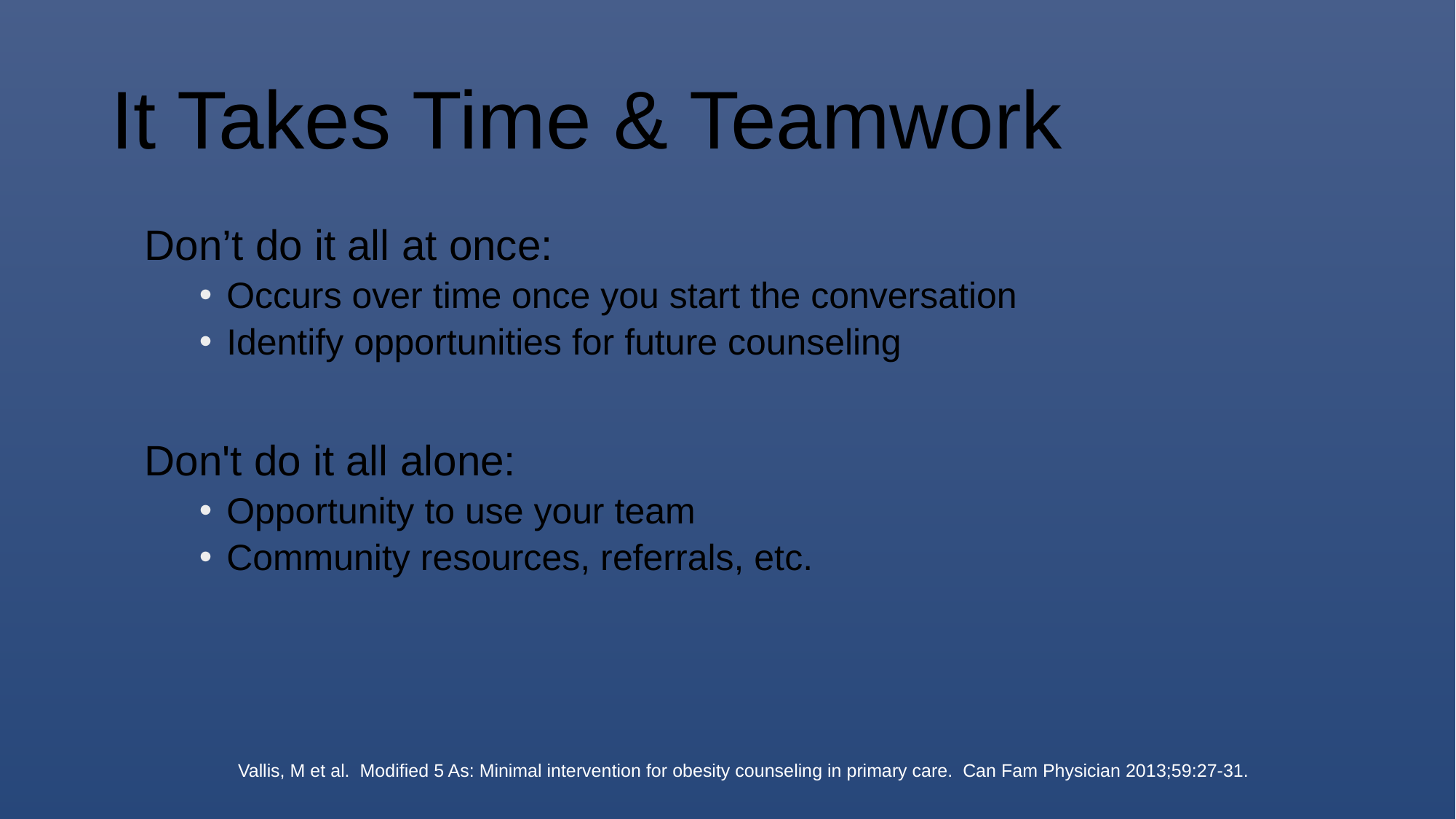

# It Takes Time & Teamwork
Don’t do it all at once:
Occurs over time once you start the conversation
Identify opportunities for future counseling
Don't do it all alone:
Opportunity to use your team
Community resources, referrals, etc.
Vallis, M et al.  Modified 5 As: Minimal intervention for obesity counseling in primary care.  Can Fam Physician 2013;59:27-31.

## Slide 26
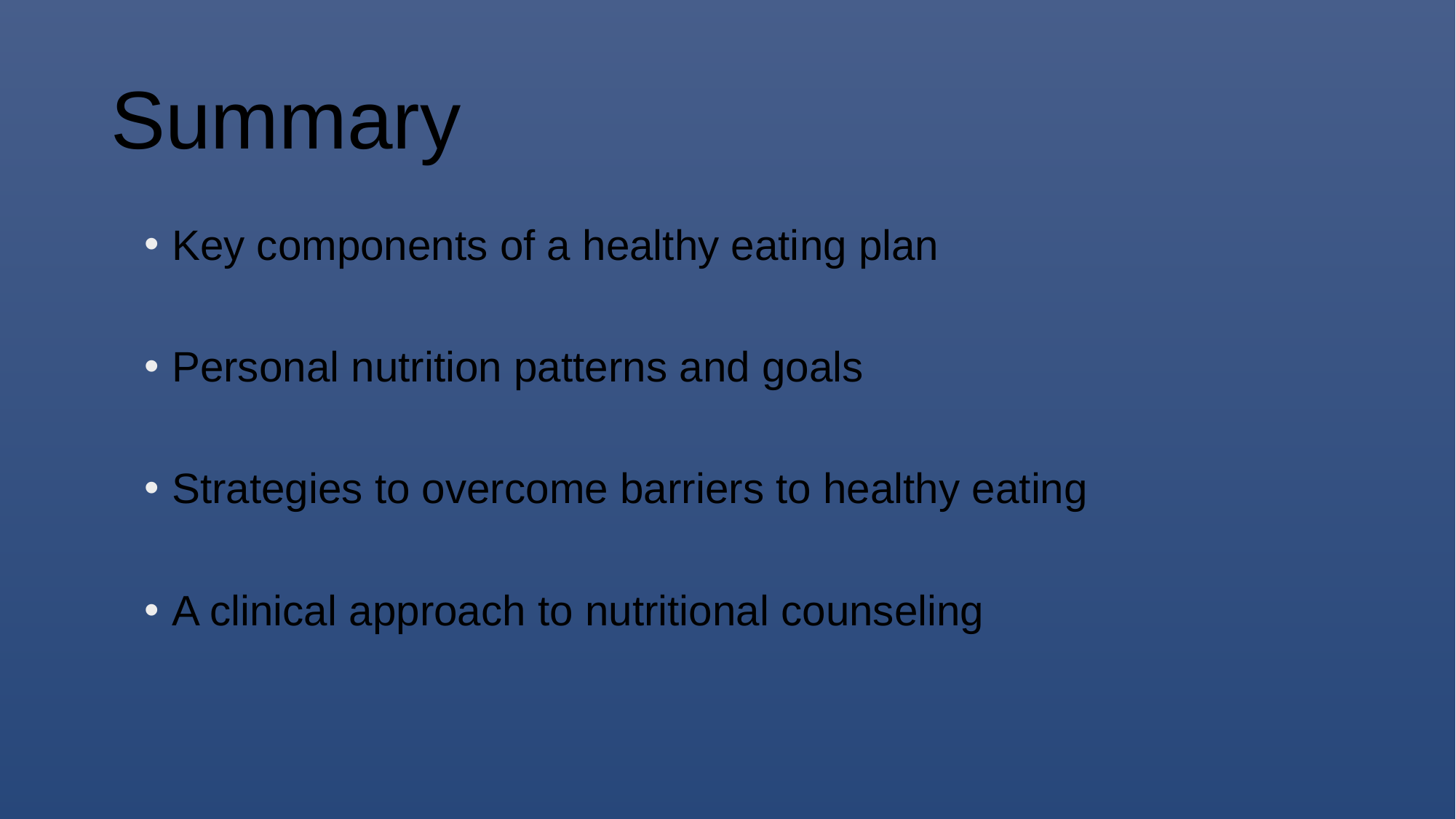

# Summary
Key components of a healthy eating plan
Personal nutrition patterns and goals
Strategies to overcome barriers to healthy eating
A clinical approach to nutritional counseling
