# Supplementary material for: An Interactive Lifestyle Medicine Curriculum for Third-Year Medical Students to Promote Student and Patient Wellness
Source: MedEdPORTAL. 2020 Sep 18;16:10972. doi: 10.15766/mep_2374-8265.10972 (PMC7499809; doi:10.15766/mep_2374-8265.10972)
Supplement: Supplementary file 1 — Introduction & Stress Management Presentation.pptxIntroduction & Stress Management Facilitator Guide.docxUnhealthy Thoughts Handout.pdfGood Things Worksheet.pdfNutrition Presentation.pptxNutrition Facilitator Guide.docxPhysical Activity Presentation.pptxPhysical Activity Facilitator Guide.docxPresession Evaluation.docxPostsession Evaluation.docxSession Evaluation.docx [file mep_2374-8265.10972-s001.zip › F. Nutrition Facilitator Guide.docx]

**Facilitator Guide**

**Lifestyle Medicine & Student Wellness: Nutrition**

Materials needed:

- AV Equipment for powerpoint presentation
- Paper plates (or blank pieces of paper)
- Multiple Flip charts or white boards

Slides 1-3: Introduction/Objectives (5 minutes)

Slide 4: Healthy Plate Activity: Ask students to draw out their dinner from last night on a paper plate, allocating the appropriate proportions to each food they ate. Ask for student volunteers who are willing to share their plate with the group. (5 minutes)

Slide 5-7: Review Obesity and Health Implications (2 minutes)

Slides 8-11: Discuss Nutritional Advice including MyPlate guidelines. (3 minutes)

Slide 12-13: Addressing Barriers to Healthy Eating Activity:

Ask students to compare their food plate to MyPlate. Within their groups, ask them to identify current barriers to maintaining healthier eating behaviors. Brainstorm as a larger group about barriers, and make a board for each one. Have students go to the board that they feel they do WELL, and write pieces of advice for their classmates. (10 minutes)

Have one person from each group report out their advice to the whole class.  (15 minutes)

*Note: We also collect this information and send it out to the students after the session.*

Slide 14: Easy Targets for Healthy Eating (1 minute)

Slides 15-25: Counseling in Clinical Practice (10-15 minutes)

*Slides 18-23: Optional: If you have not already discussed the 5 A’s Framework for Behavior Change in your curriculum, you can use these slides to briefly go through the steps. In the past we utilized a role play, but we eliminated it due to time constraints and based on student feedback.*

Slide 26: Summary and Questions (5 minutes with time for questions)
