# Supplementary material for: An Interactive Lifestyle Medicine Curriculum for Third-Year Medical Students to Promote Student and Patient Wellness
Source: MedEdPORTAL. 2020 Sep 18;16:10972. doi: 10.15766/mep_2374-8265.10972 (PMC7499809; doi:10.15766/mep_2374-8265.10972)
Supplement: Supplementary file 1 — Introduction & Stress Management Presentation.pptxIntroduction & Stress Management Facilitator Guide.docxUnhealthy Thoughts Handout.pdfGood Things Worksheet.pdfNutrition Presentation.pptxNutrition Facilitator Guide.docxPhysical Activity Presentation.pptxPhysical Activity Facilitator Guide.docxPresession Evaluation.docxPostsession Evaluation.docxSession Evaluation.docx [file mep_2374-8265.10972-s001.zip › G. Physical Activity Presentation.pptx]

## Slide 1
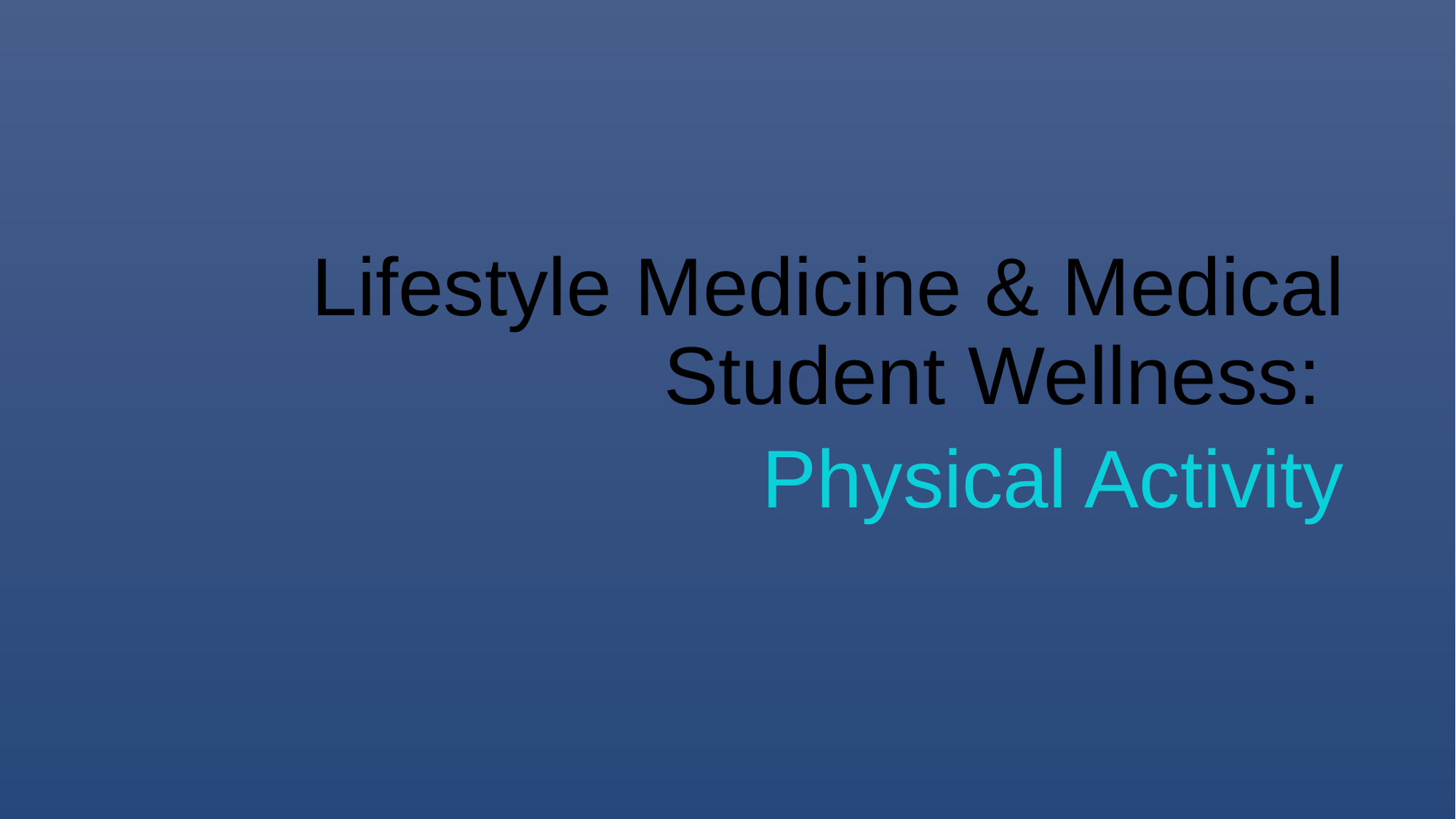

Lifestyle Medicine & Medical Student Wellness:
Physical Activity

## Slide 2
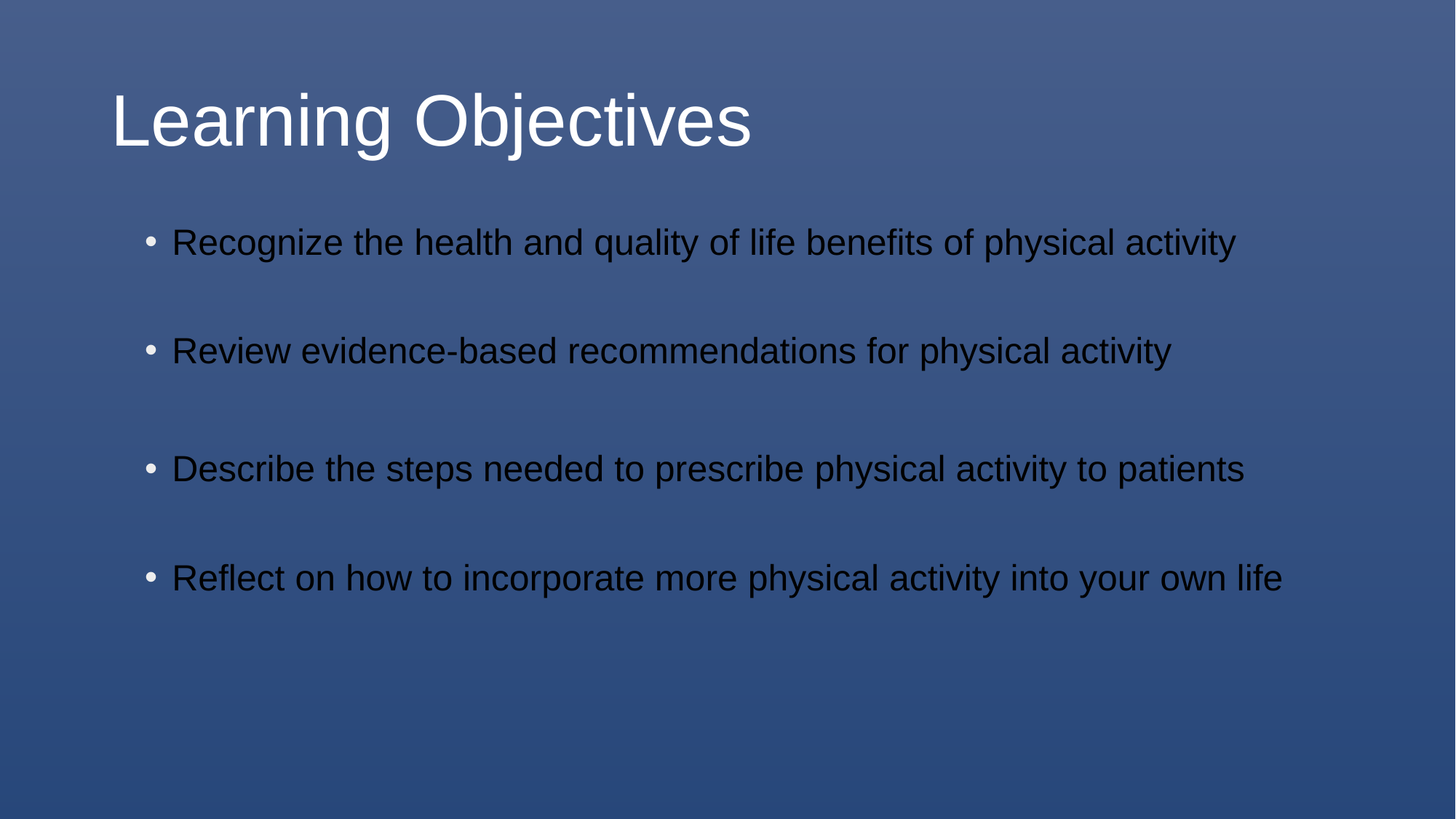

# Learning Objectives
Recognize the health and quality of life benefits of physical activity
Review evidence-based recommendations for physical activity
Describe the steps needed to prescribe physical activity to patients
Reflect on how to incorporate more physical activity into your own life

## Slide 3
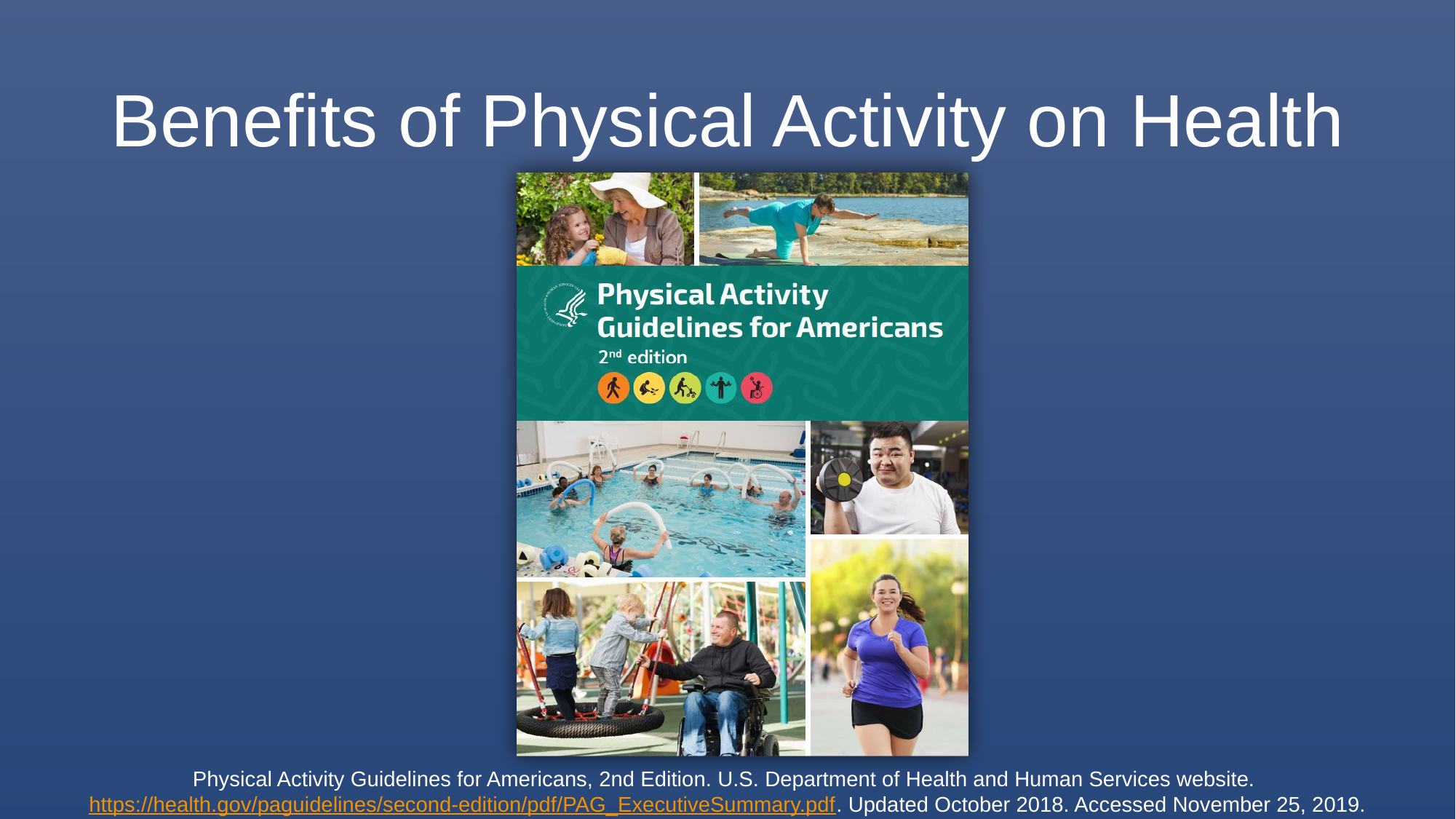

# Benefits of Physical Activity on Health
Physical Activity Guidelines for Americans, 2nd Edition. U.S. Department of Health and Human Services website. https://health.gov/paguidelines/second-edition/pdf/PAG_ExecutiveSummary.pdf. Updated October 2018. Accessed November 25, 2019.

## Slide 4
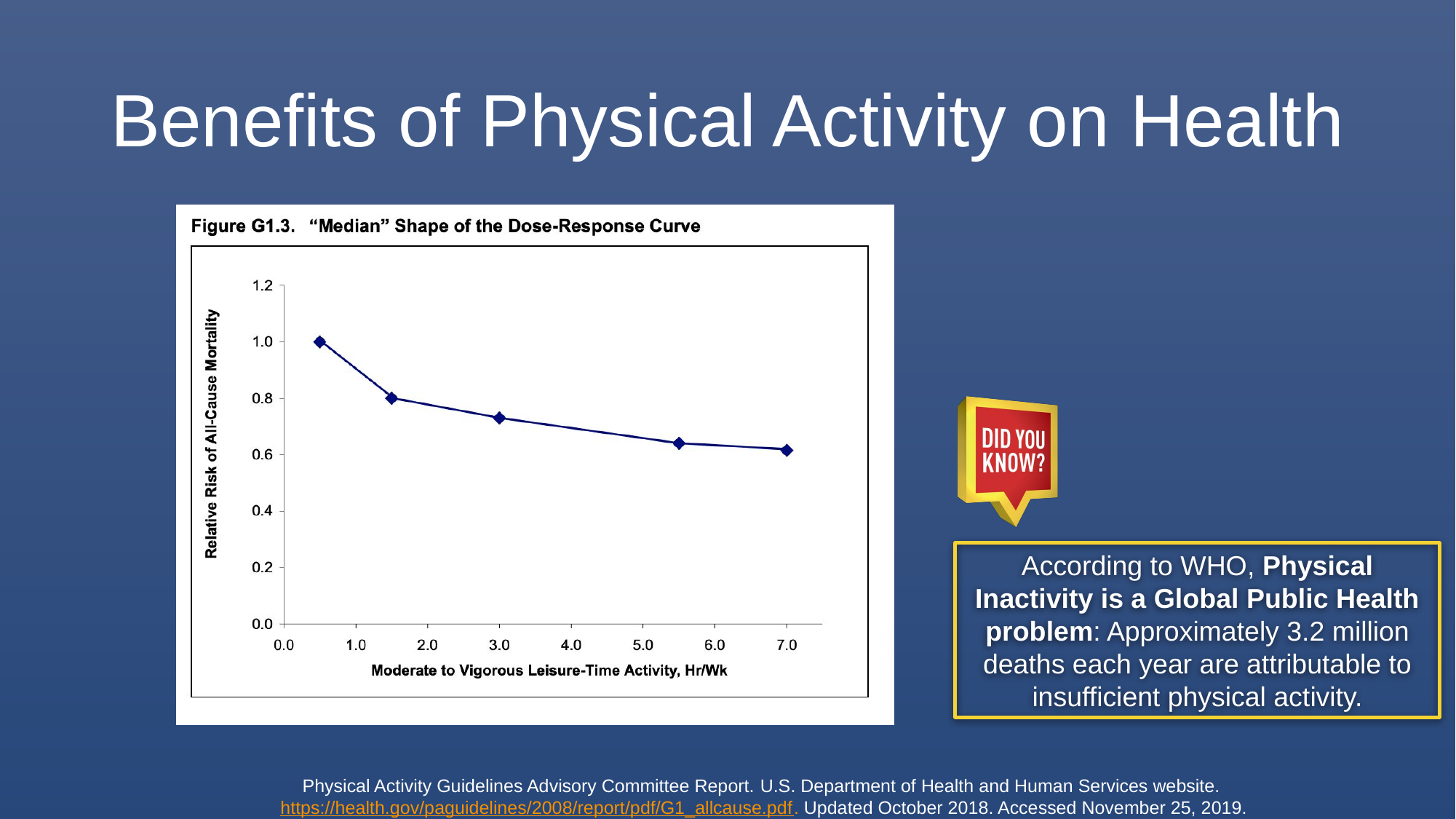

# Benefits of Physical Activity on Health
Click to add text
According to WHO, Physical Inactivity is a Global Public Health problem: Approximately 3.2 million deaths each year are attributable to insufficient physical activity.
Physical Activity Guidelines Advisory Committee Report. U.S. Department of Health and Human Services website. https://health.gov/paguidelines/2008/report/pdf/G1_allcause.pdf. Updated October 2018. Accessed November 25, 2019.

## Slide 5
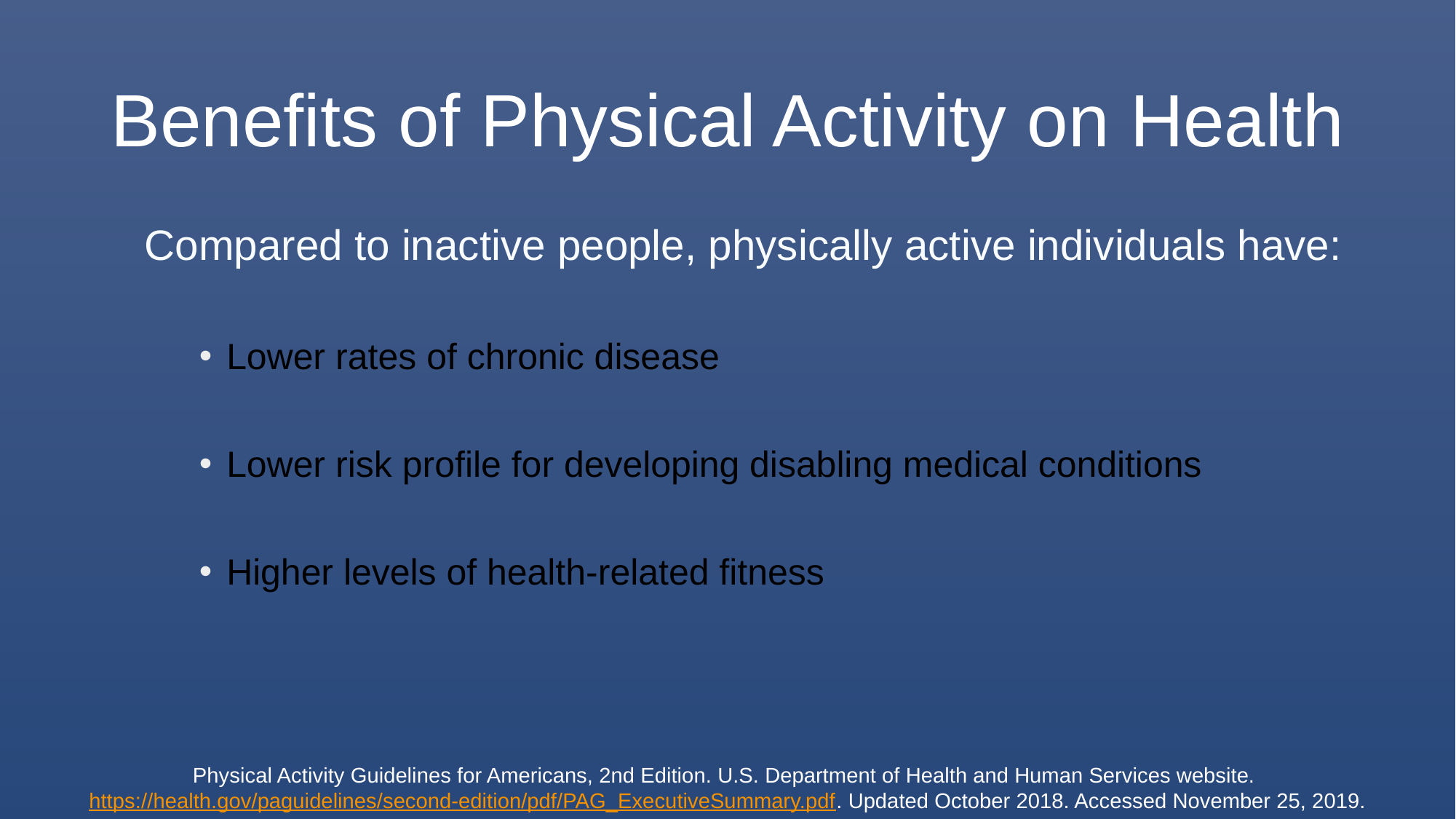

# Benefits of Physical Activity on Health
Compared to inactive people, physically active individuals have:
Lower rates of chronic disease
Lower risk profile for developing disabling medical conditions
Higher levels of health-related fitness
Physical Activity Guidelines for Americans, 2nd Edition. U.S. Department of Health and Human Services website. https://health.gov/paguidelines/second-edition/pdf/PAG_ExecutiveSummary.pdf. Updated October 2018. Accessed November 25, 2019.

## Slide 6
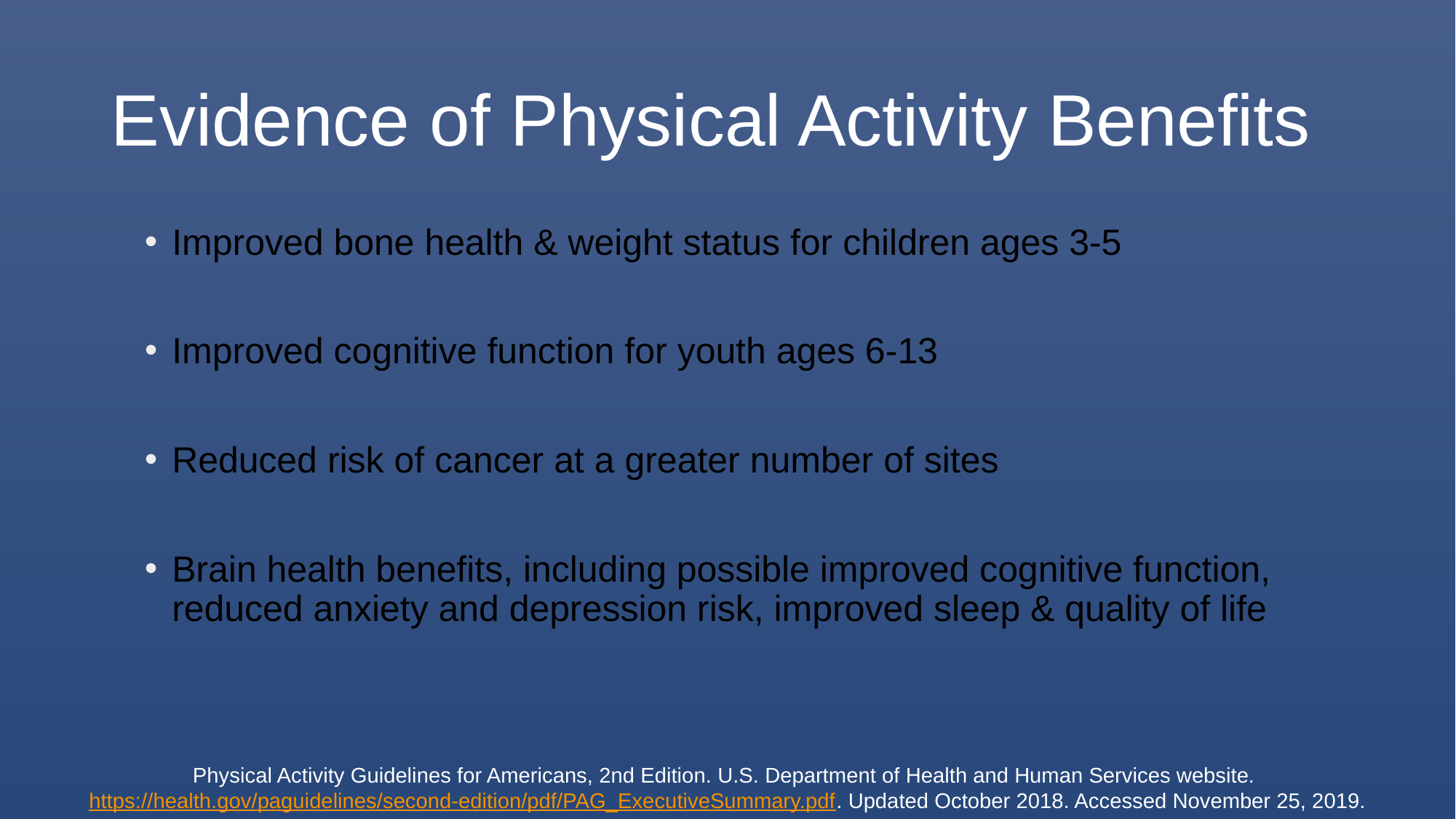

Evidence of Physical Activity Benefits
Improved bone health & weight status for children ages 3-5
Improved cognitive function for youth ages 6-13
Reduced risk of cancer at a greater number of sites
Brain health benefits, including possible improved cognitive function, reduced anxiety and depression risk, improved sleep & quality of life
Physical Activity Guidelines for Americans, 2nd Edition. U.S. Department of Health and Human Services website. https://health.gov/paguidelines/second-edition/pdf/PAG_ExecutiveSummary.pdf. Updated October 2018. Accessed November 25, 2019.

## Slide 7
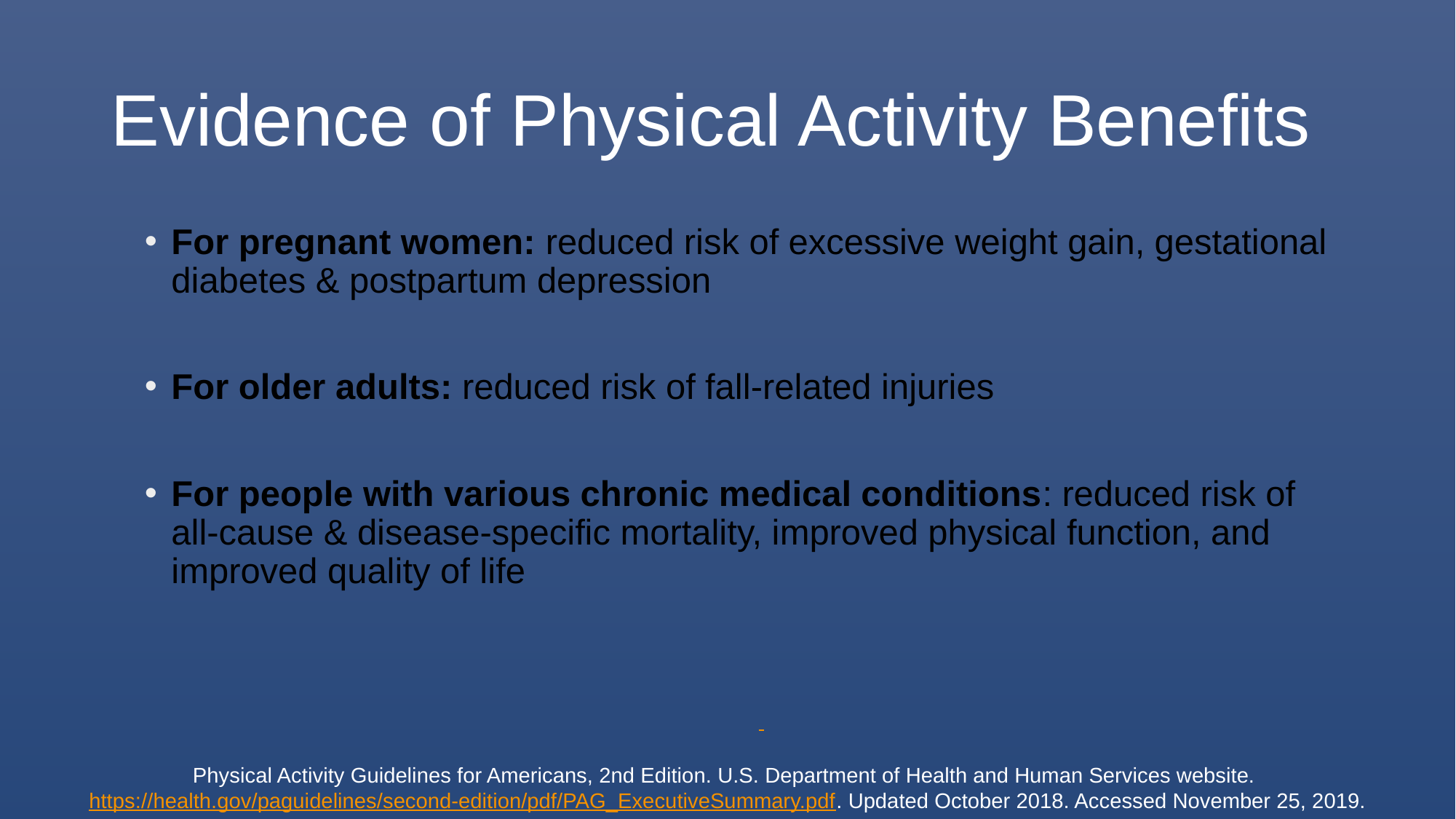

Evidence of Physical Activity Benefits
For pregnant women: reduced risk of excessive weight gain, gestational diabetes & postpartum depression
For older adults: reduced risk of fall-related injuries
For people with various chronic medical conditions: reduced risk of all-cause & disease-specific mortality, improved physical function, and improved quality of life
Physical Activity Guidelines for Americans, 2nd Edition. U.S. Department of Health and Human Services website. https://health.gov/paguidelines/second-edition/pdf/PAG_ExecutiveSummary.pdf. Updated October 2018. Accessed November 25, 2019.

## Slide 8
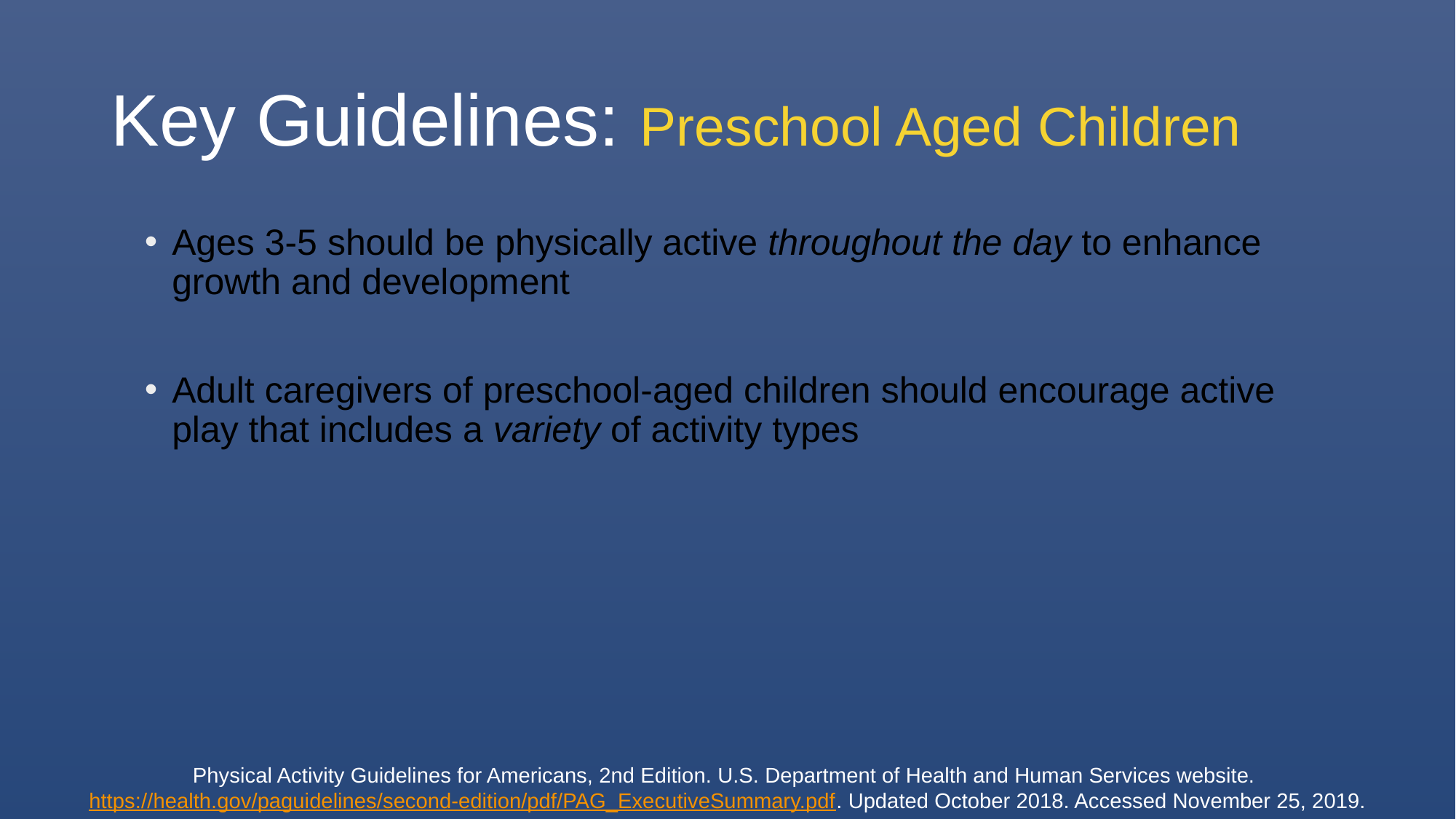

# Key Guidelines: Preschool Aged Children
Ages 3-5 should be physically active throughout the day to enhance growth and development
Adult caregivers of preschool-aged children should encourage active play that includes a variety of activity types
Physical Activity Guidelines for Americans, 2nd Edition. U.S. Department of Health and Human Services website. https://health.gov/paguidelines/second-edition/pdf/PAG_ExecutiveSummary.pdf. Updated October 2018. Accessed November 25, 2019.

## Slide 9
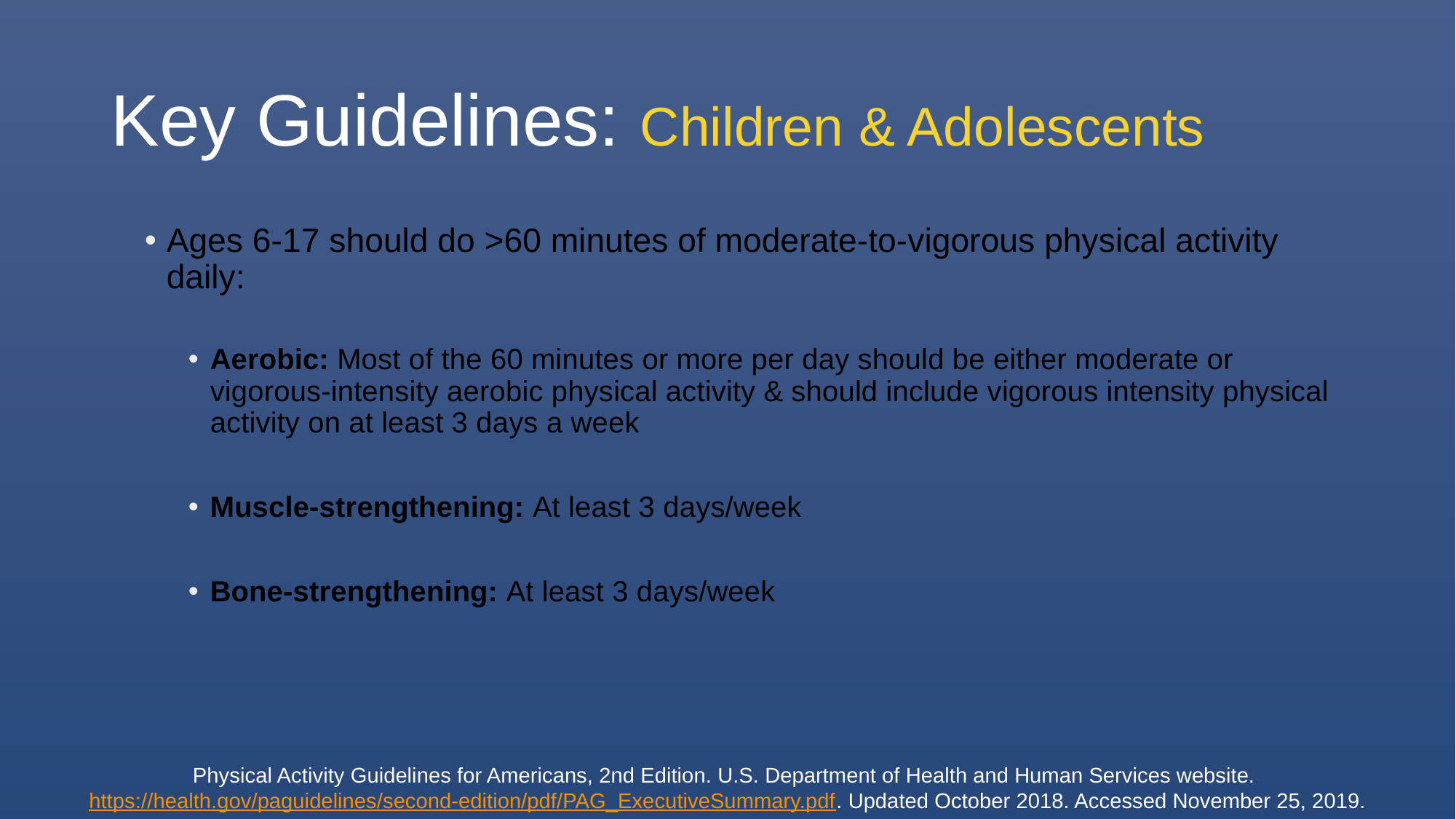

# Key Guidelines: Children & Adolescents
Ages 6-17 should do >60 minutes of moderate-to-vigorous physical activity daily:
Aerobic: Most of the 60 minutes or more per day should be either moderate or vigorous-intensity aerobic physical activity & should include vigorous intensity physical activity on at least 3 days a week
Muscle-strengthening: At least 3 days/week
Bone-strengthening: At least 3 days/week
Physical Activity Guidelines for Americans, 2nd Edition. U.S. Department of Health and Human Services website. https://health.gov/paguidelines/second-edition/pdf/PAG_ExecutiveSummary.pdf. Updated October 2018. Accessed November 25, 2019.

## Slide 10
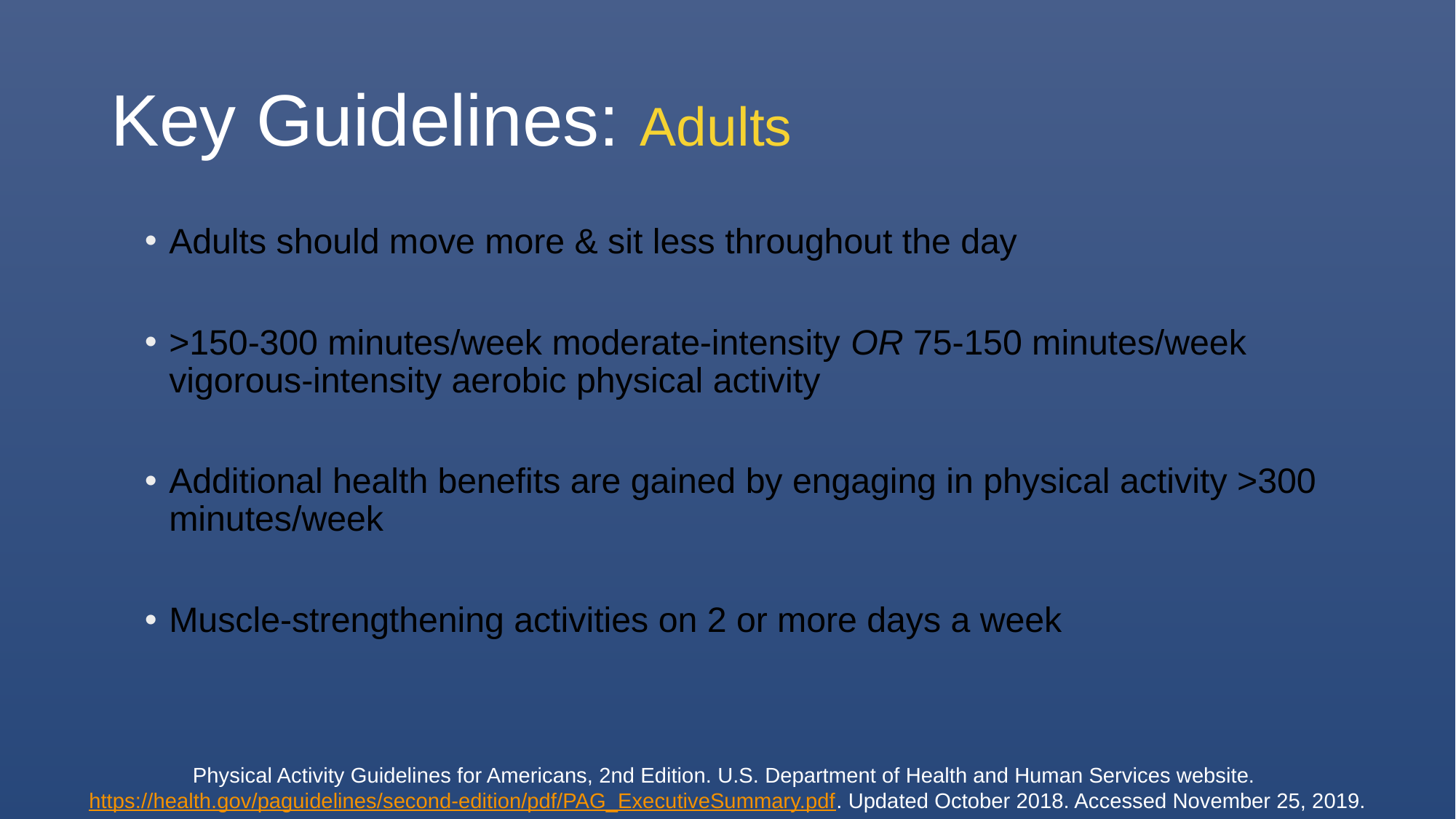

# Key Guidelines: Adults
Adults should move more & sit less throughout the day
>150-300 minutes/week moderate-intensity OR 75-150 minutes/week vigorous-intensity aerobic physical activity
Additional health benefits are gained by engaging in physical activity >300 minutes/week
Muscle-strengthening activities on 2 or more days a week
Physical Activity Guidelines for Americans, 2nd Edition. U.S. Department of Health and Human Services website. https://health.gov/paguidelines/second-edition/pdf/PAG_ExecutiveSummary.pdf. Updated October 2018. Accessed November 25, 2019.

## Slide 11
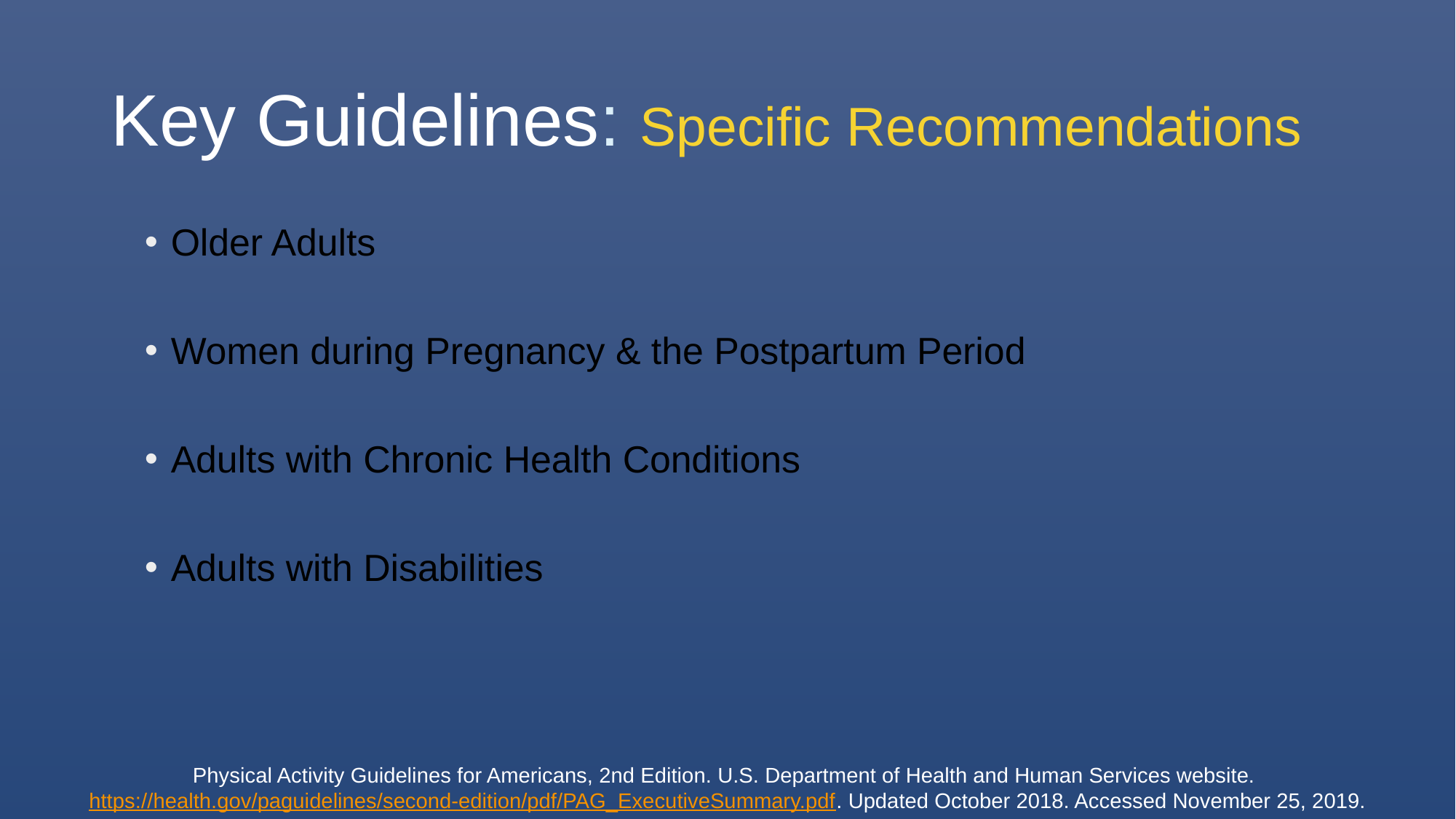

# Key Guidelines: Specific Recommendations
Older Adults
Women during Pregnancy & the Postpartum Period
Adults with Chronic Health Conditions
Adults with Disabilities
Physical Activity Guidelines for Americans, 2nd Edition. U.S. Department of Health and Human Services website. https://health.gov/paguidelines/second-edition/pdf/PAG_ExecutiveSummary.pdf. Updated October 2018. Accessed November 25, 2019.

## Slide 12
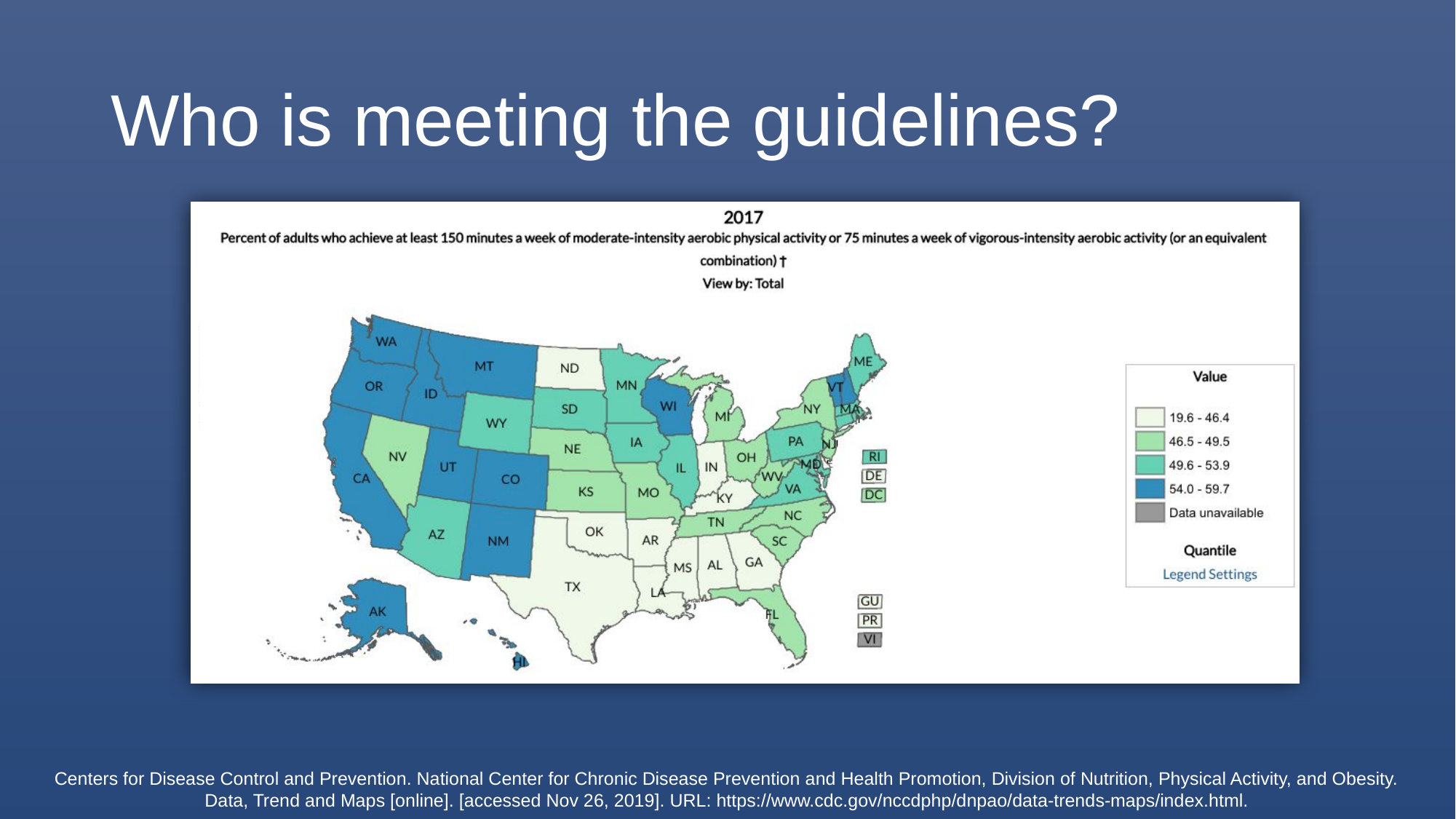

# Who is meeting the guidelines?
Centers for Disease Control and Prevention. National Center for Chronic Disease Prevention and Health Promotion, Division of Nutrition, Physical Activity, and Obesity. Data, Trend and Maps [online]. [accessed Nov 26, 2019]. URL: https://www.cdc.gov/nccdphp/dnpao/data-trends-maps/index.html.

## Slide 13
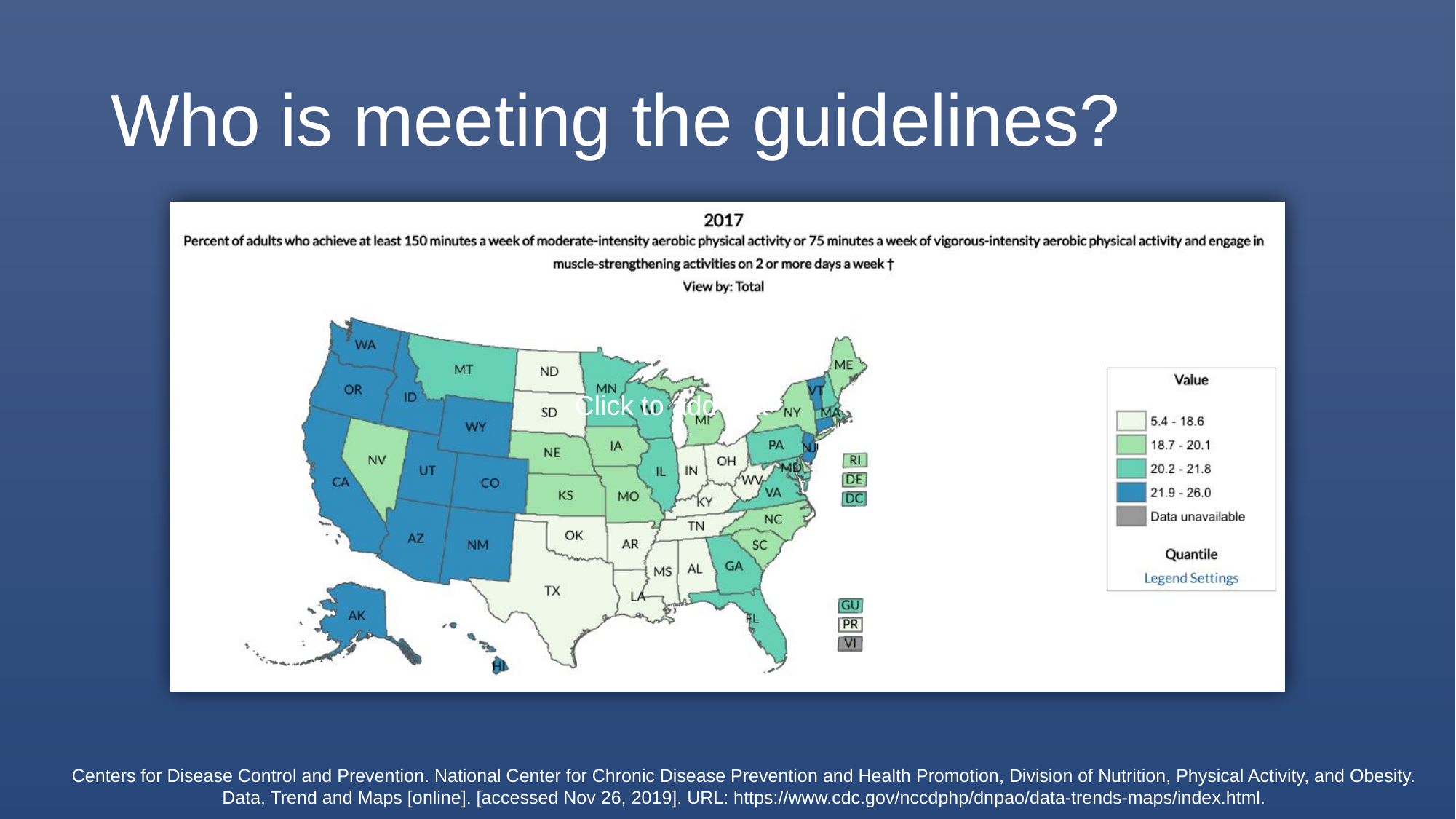

# Who is meeting the guidelines?
Click to add text
Centers for Disease Control and Prevention. National Center for Chronic Disease Prevention and Health Promotion, Division of Nutrition, Physical Activity, and Obesity. Data, Trend and Maps [online]. [accessed Nov 26, 2019]. URL: https://www.cdc.gov/nccdphp/dnpao/data-trends-maps/index.html.

## Slide 14
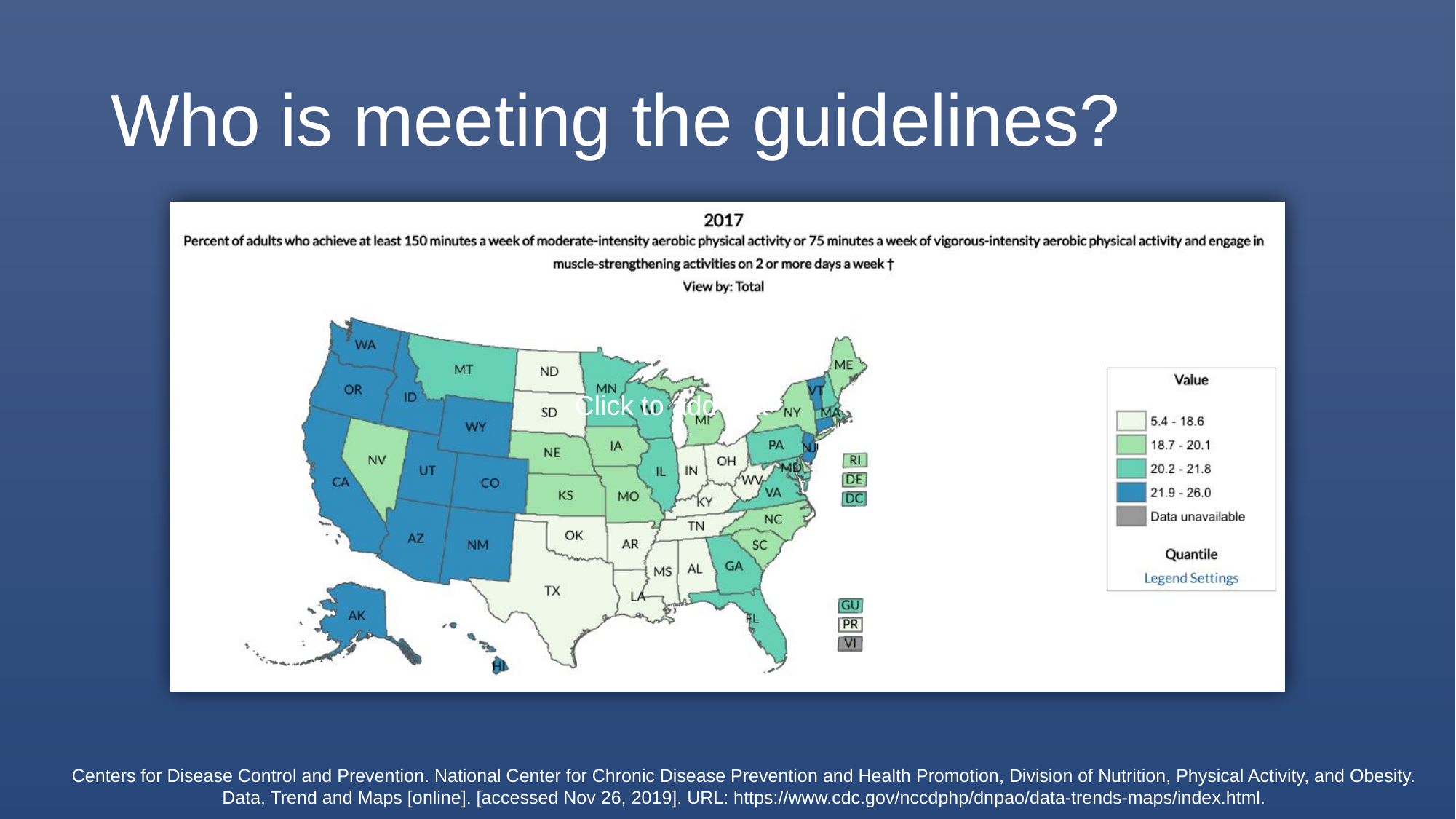

# Who is meeting the guidelines?
Click to add text
Centers for Disease Control and Prevention. National Center for Chronic Disease Prevention and Health Promotion, Division of Nutrition, Physical Activity, and Obesity. Data, Trend and Maps [online]. [accessed Nov 26, 2019]. URL: https://www.cdc.gov/nccdphp/dnpao/data-trends-maps/index.html.

## Slide 15
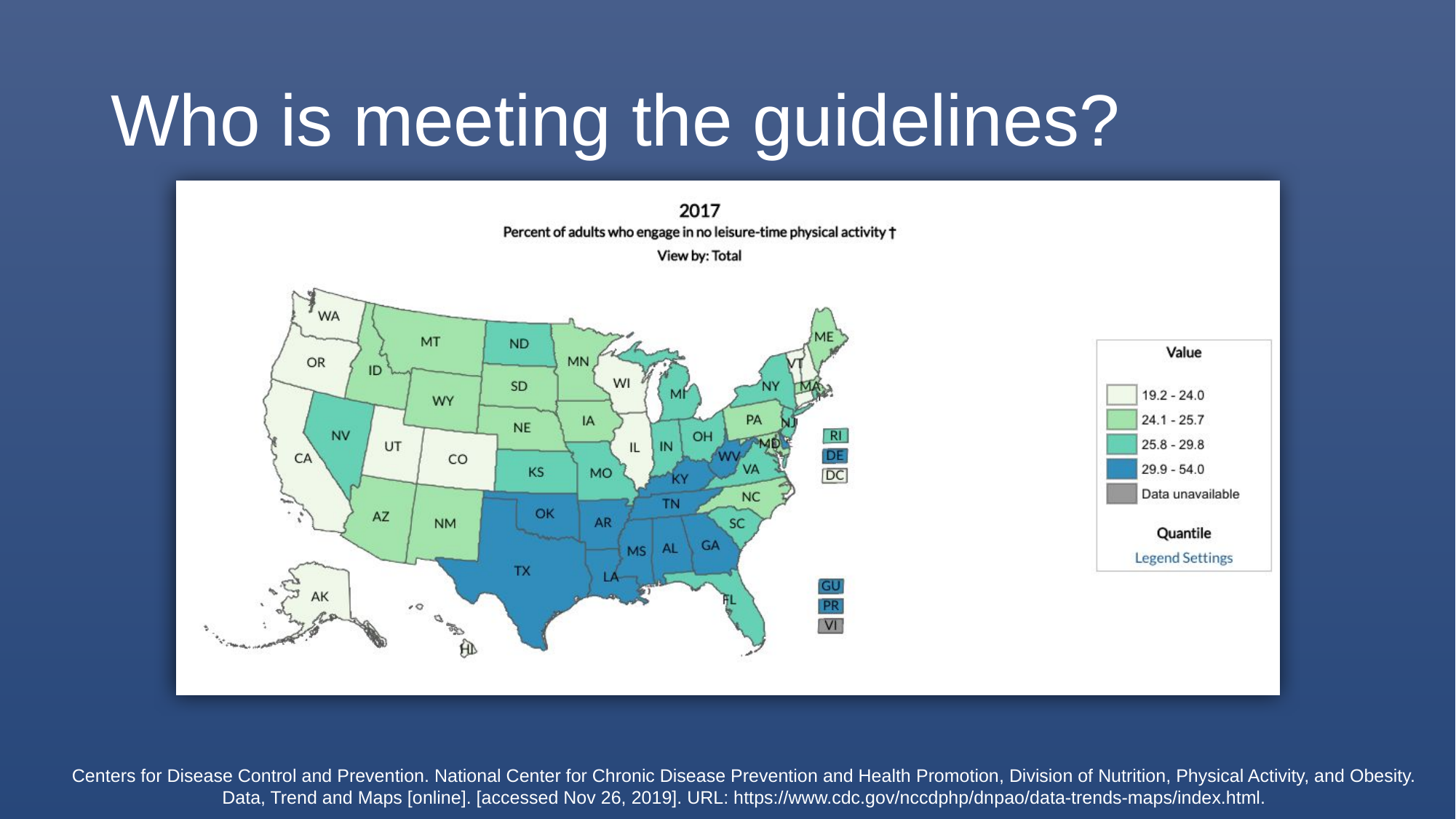

# Who is meeting the guidelines?
Centers for Disease Control and Prevention. National Center for Chronic Disease Prevention and Health Promotion, Division of Nutrition, Physical Activity, and Obesity. Data, Trend and Maps [online]. [accessed Nov 26, 2019]. URL: https://www.cdc.gov/nccdphp/dnpao/data-trends-maps/index.html.

## Slide 16
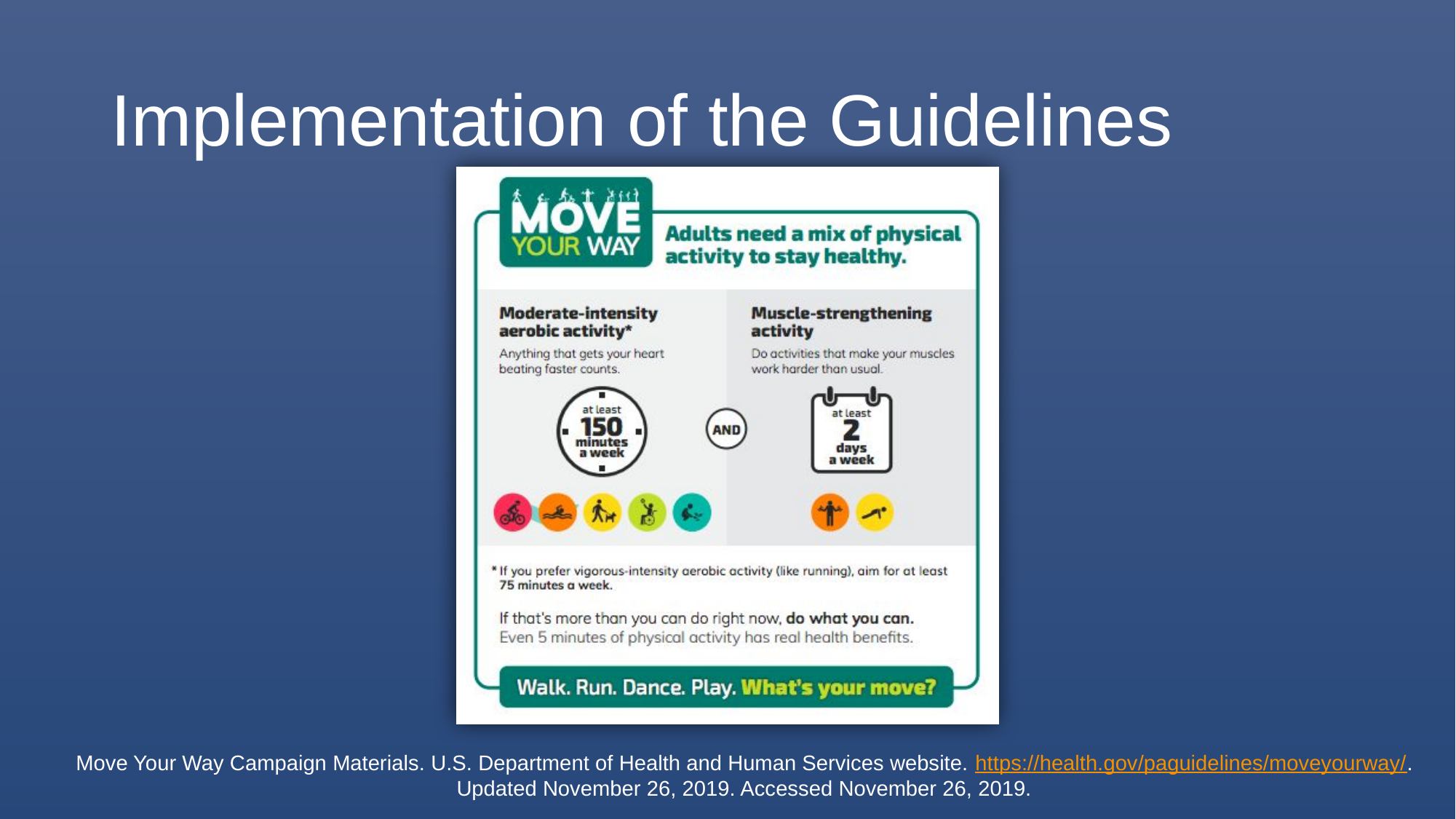

# Implementation of the Guidelines
Move Your Way Campaign Materials. U.S. Department of Health and Human Services website. https://health.gov/paguidelines/moveyourway/. Updated November 26, 2019. Accessed November 26, 2019.

## Slide 17
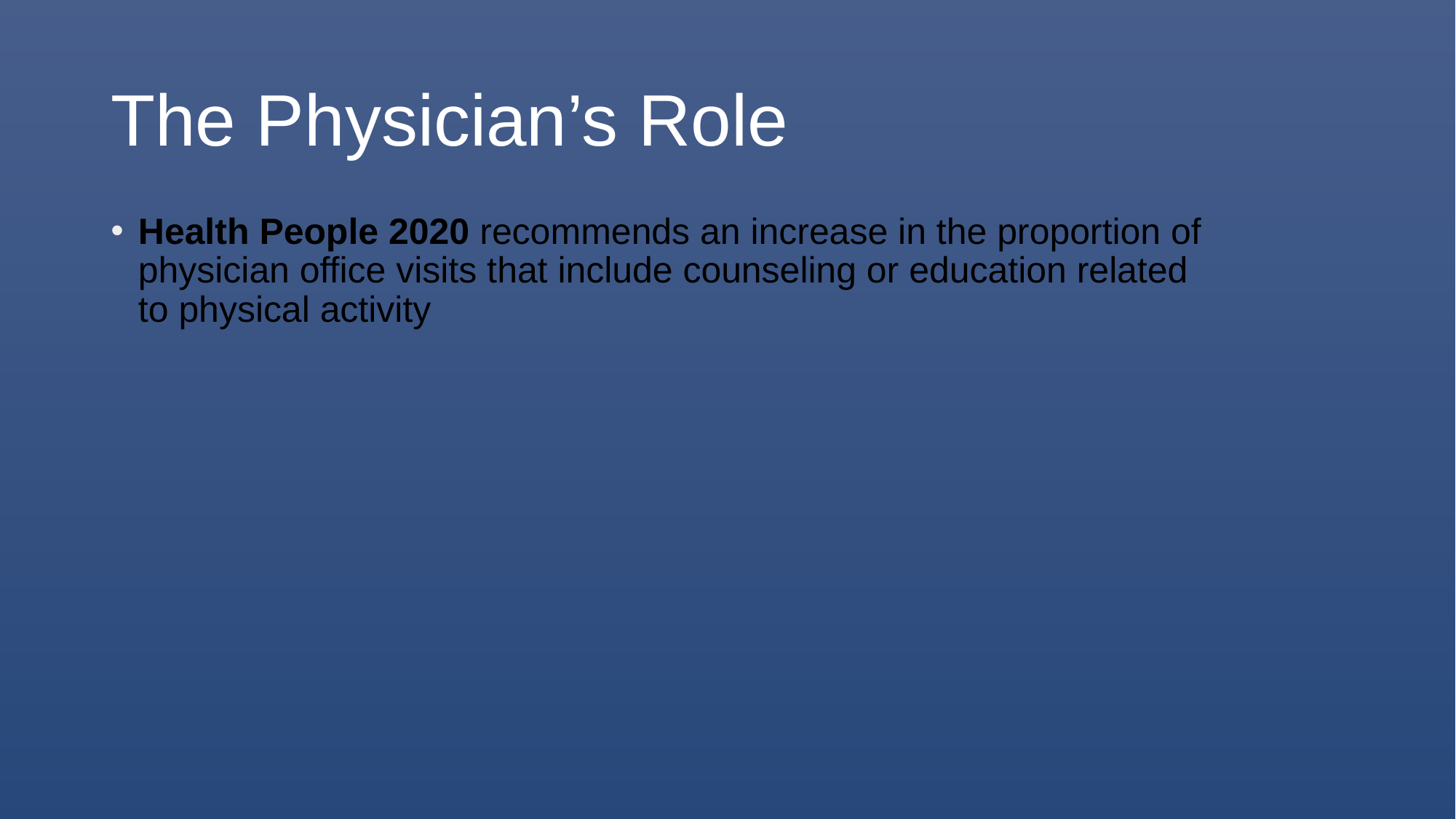

# The Physician’s Role
Health People 2020 recommends an increase in the proportion of physician office visits that include counseling or education related to physical activity

## Slide 18
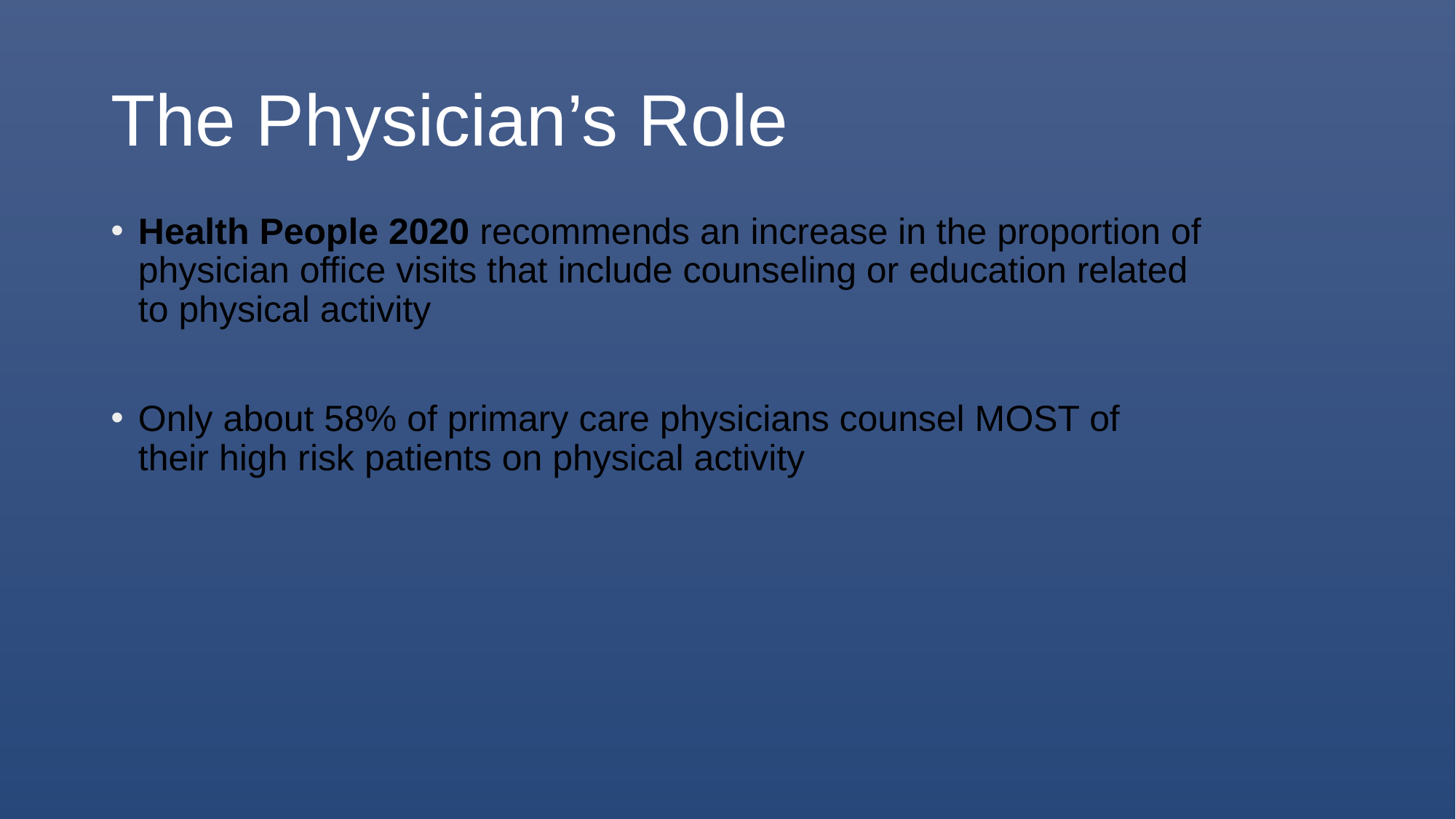

# The Physician’s Role
Health People 2020 recommends an increase in the proportion of physician office visits that include counseling or education related to physical activity
Only about 58% of primary care physicians counsel MOST of their high risk patients on physical activity

## Slide 19
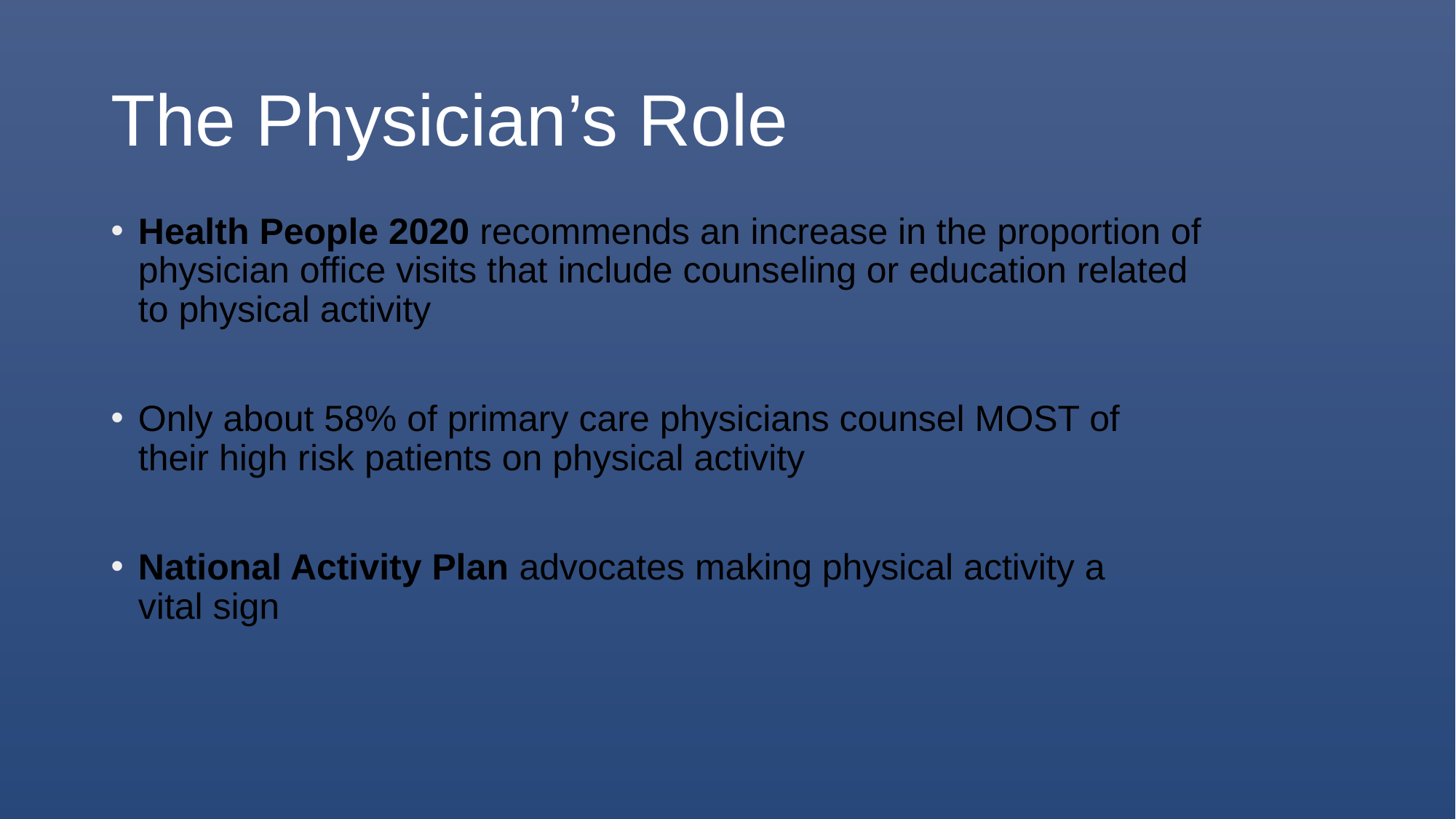

# The Physician’s Role
Health People 2020 recommends an increase in the proportion of physician office visits that include counseling or education related to physical activity
Only about 58% of primary care physicians counsel MOST of their high risk patients on physical activity
National Activity Plan advocates making physical activity a vital sign

## Slide 20
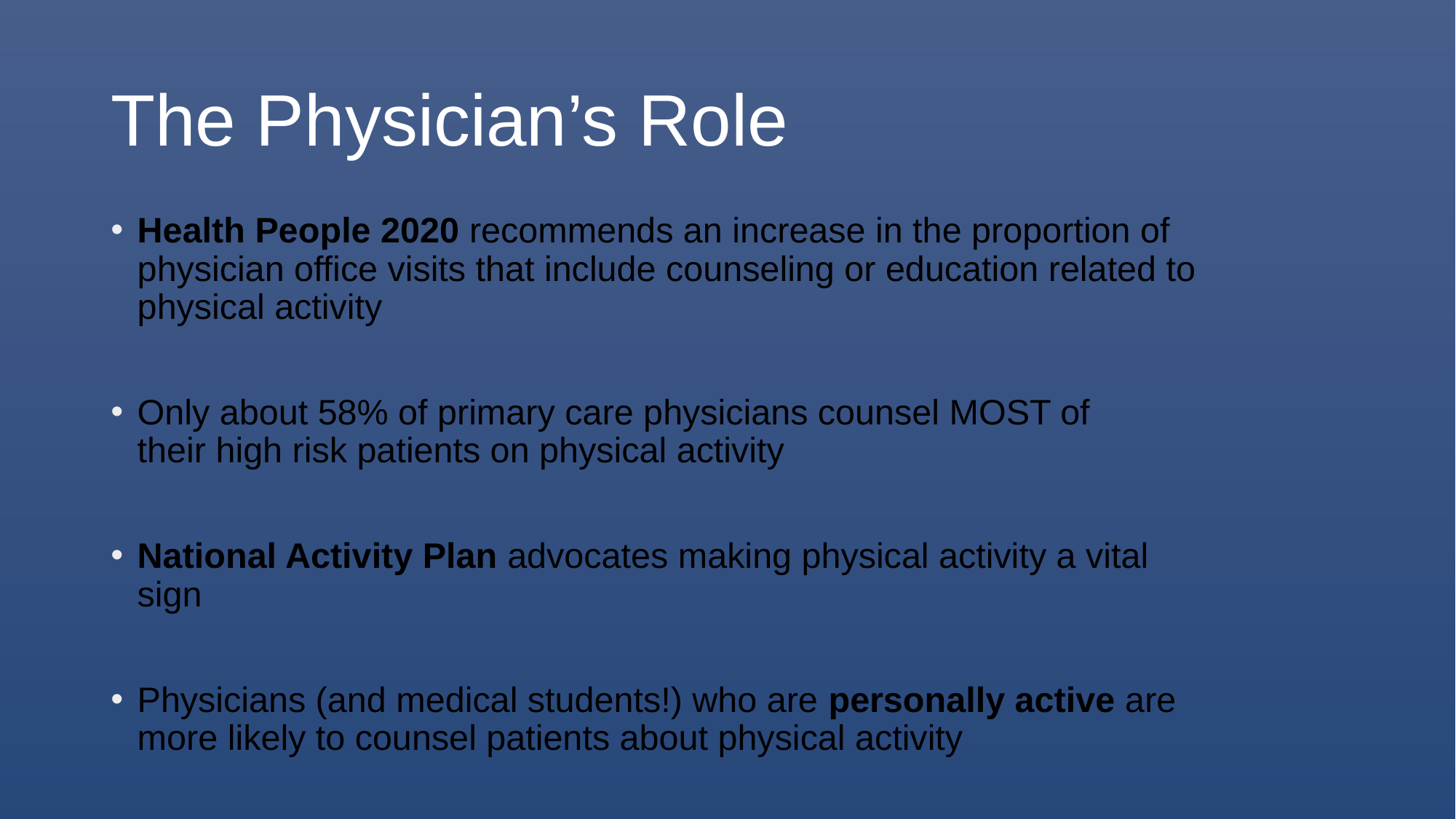

# The Physician’s Role
Health People 2020 recommends an increase in the proportion of physician office visits that include counseling or education related to physical activity
Only about 58% of primary care physicians counsel MOST of their high risk patients on physical activity
National Activity Plan advocates making physical activity a vital sign
Physicians (and medical students!) who are personally active are more likely to counsel patients about physical activity

## Slide 21
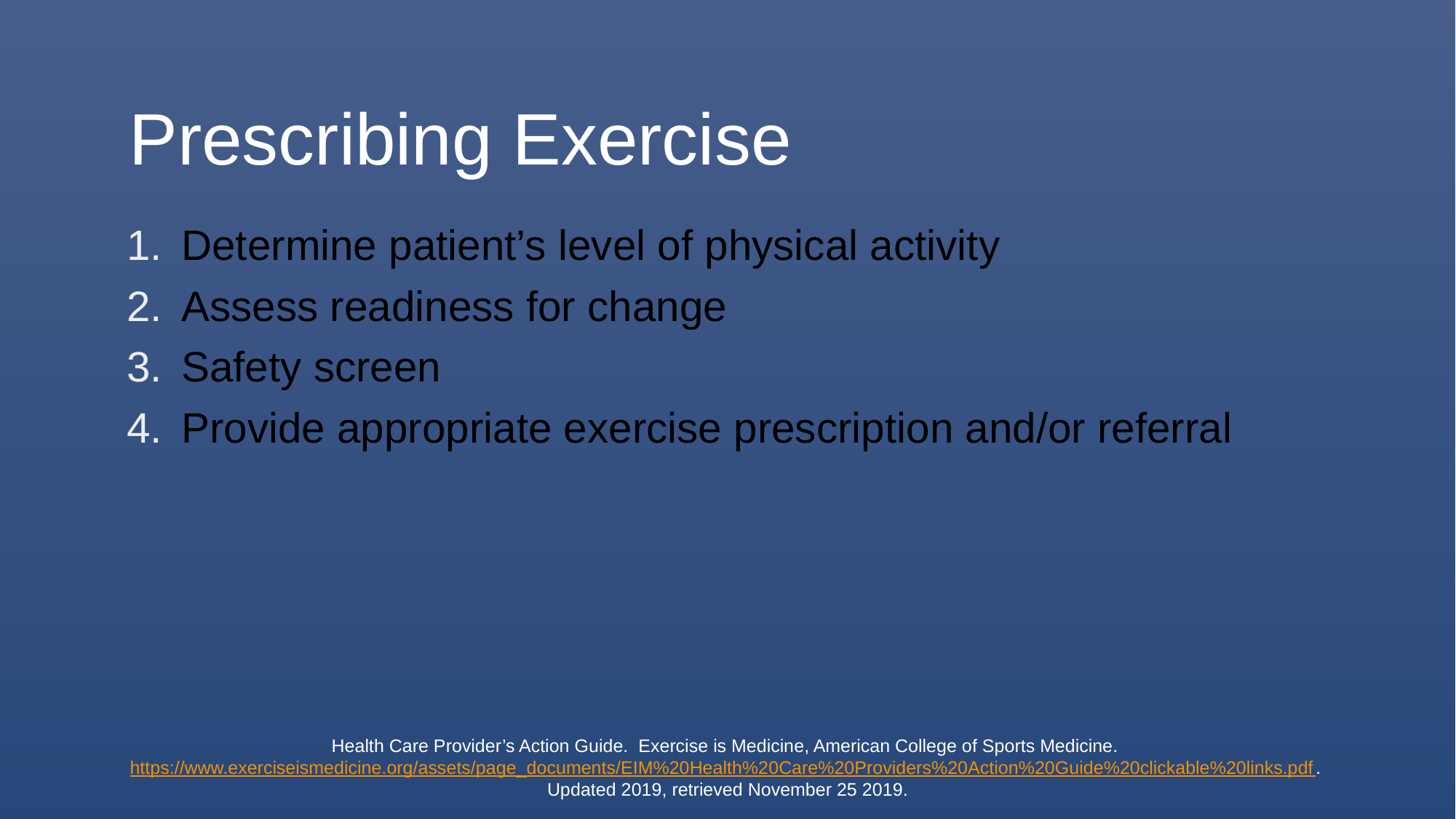

Prescribing Exercise
Determine patient’s level of physical activity
Assess readiness for change
Safety screen
Provide appropriate exercise prescription and/or referral
Health Care Provider’s Action Guide. Exercise is Medicine, American College of Sports Medicine. https://www.exerciseismedicine.org/assets/page_documents/EIM%20Health%20Care%20Providers%20Action%20Guide%20clickable%20links.pdf. Updated 2019, retrieved November 25 2019.

## Slide 22
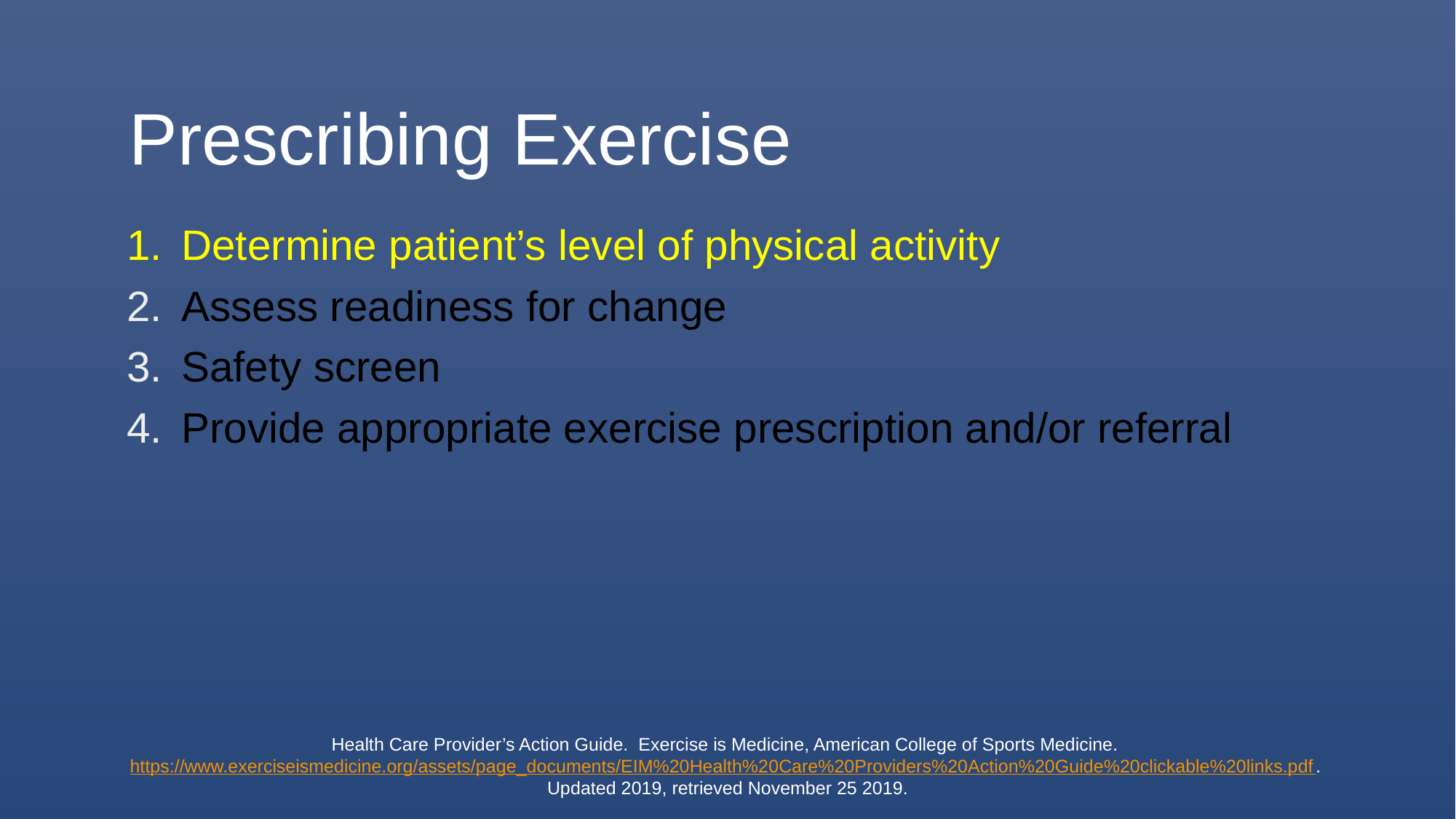

Prescribing Exercise
Determine patient’s level of physical activity
Assess readiness for change
Safety screen
Provide appropriate exercise prescription and/or referral
Health Care Provider’s Action Guide. Exercise is Medicine, American College of Sports Medicine. https://www.exerciseismedicine.org/assets/page_documents/EIM%20Health%20Care%20Providers%20Action%20Guide%20clickable%20links.pdf. Updated 2019, retrieved November 25 2019.

## Slide 23
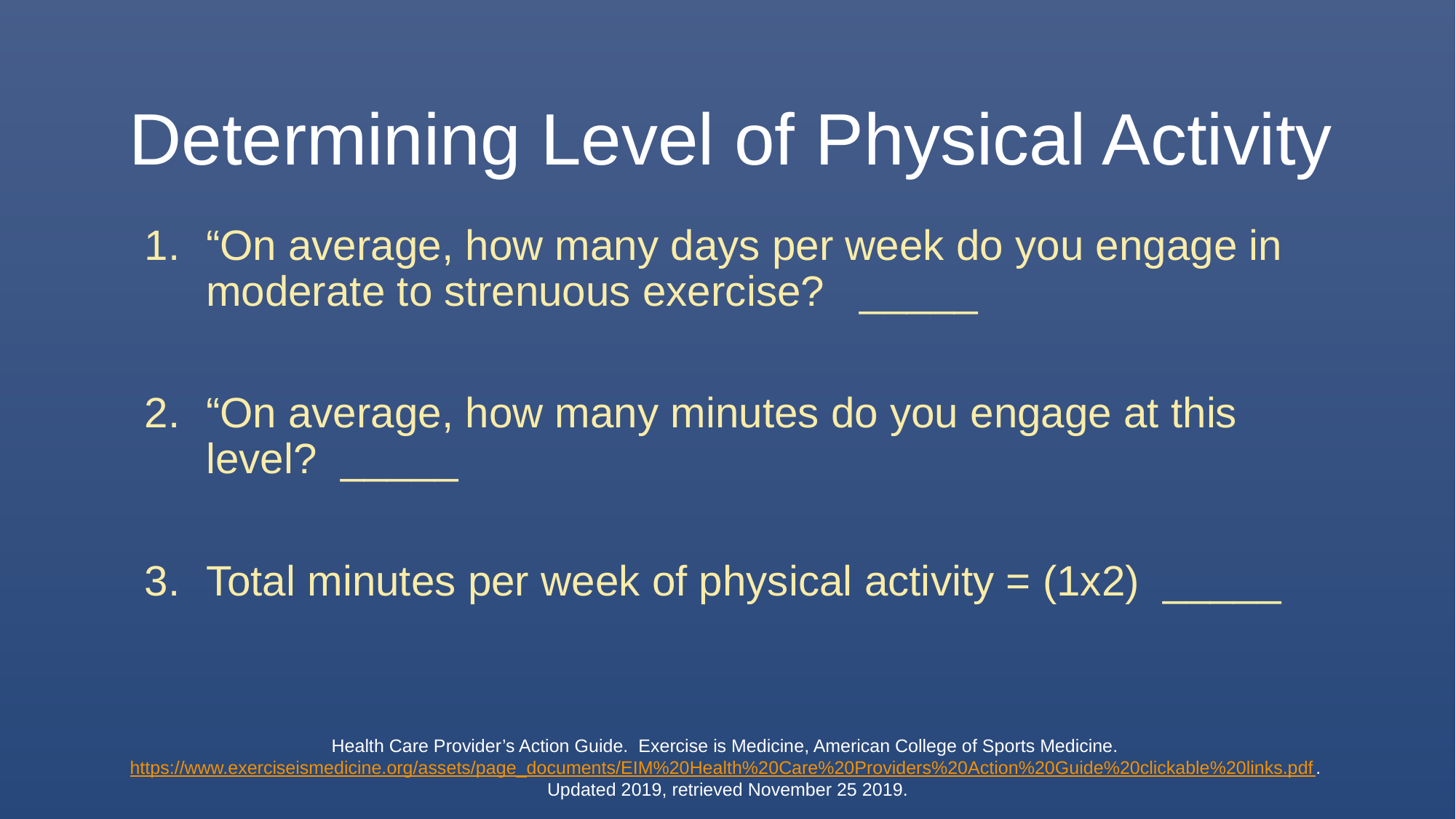

Determining Level of Physical Activity
“On average, how many days per week do you engage in moderate to strenuous exercise? _____
“On average, how many minutes do you engage at this level? _____
Total minutes per week of physical activity = (1x2) _____
Health Care Provider’s Action Guide. Exercise is Medicine, American College of Sports Medicine. https://www.exerciseismedicine.org/assets/page_documents/EIM%20Health%20Care%20Providers%20Action%20Guide%20clickable%20links.pdf. Updated 2019, retrieved November 25 2019.

## Slide 24
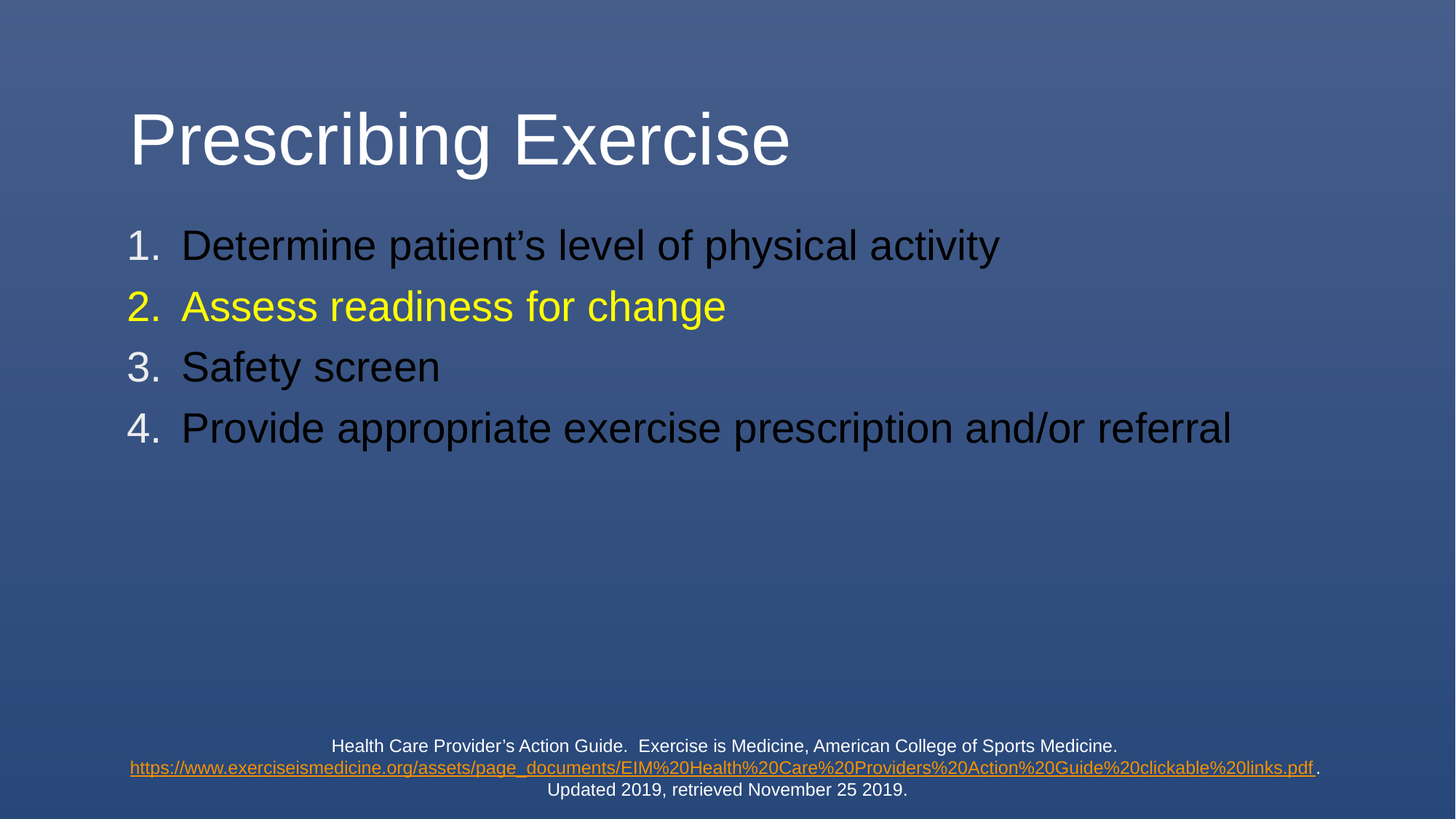

Prescribing Exercise
Determine patient’s level of physical activity
Assess readiness for change
Safety screen
Provide appropriate exercise prescription and/or referral
Health Care Provider’s Action Guide. Exercise is Medicine, American College of Sports Medicine. https://www.exerciseismedicine.org/assets/page_documents/EIM%20Health%20Care%20Providers%20Action%20Guide%20clickable%20links.pdf. Updated 2019, retrieved November 25 2019.

## Slide 25
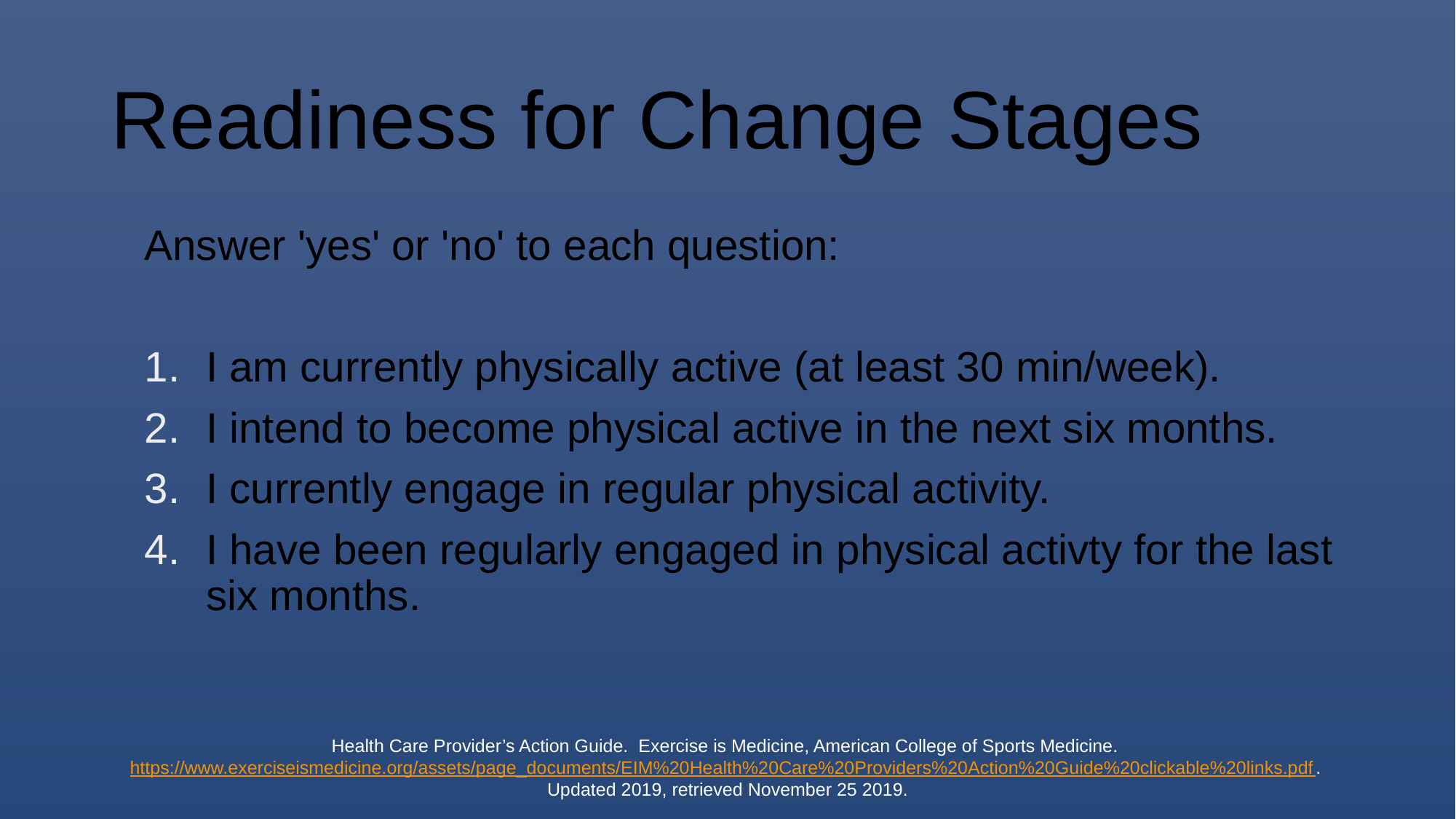

# Readiness for Change Stages
Answer 'yes' or 'no' to each question:
I am currently physically active (at least 30 min/week).
I intend to become physical active in the next six months.
I currently engage in regular physical activity.
I have been regularly engaged in physical activty for the last six months.
Health Care Provider’s Action Guide. Exercise is Medicine, American College of Sports Medicine. https://www.exerciseismedicine.org/assets/page_documents/EIM%20Health%20Care%20Providers%20Action%20Guide%20clickable%20links.pdf. Updated 2019, retrieved November 25 2019.

## Slide 26
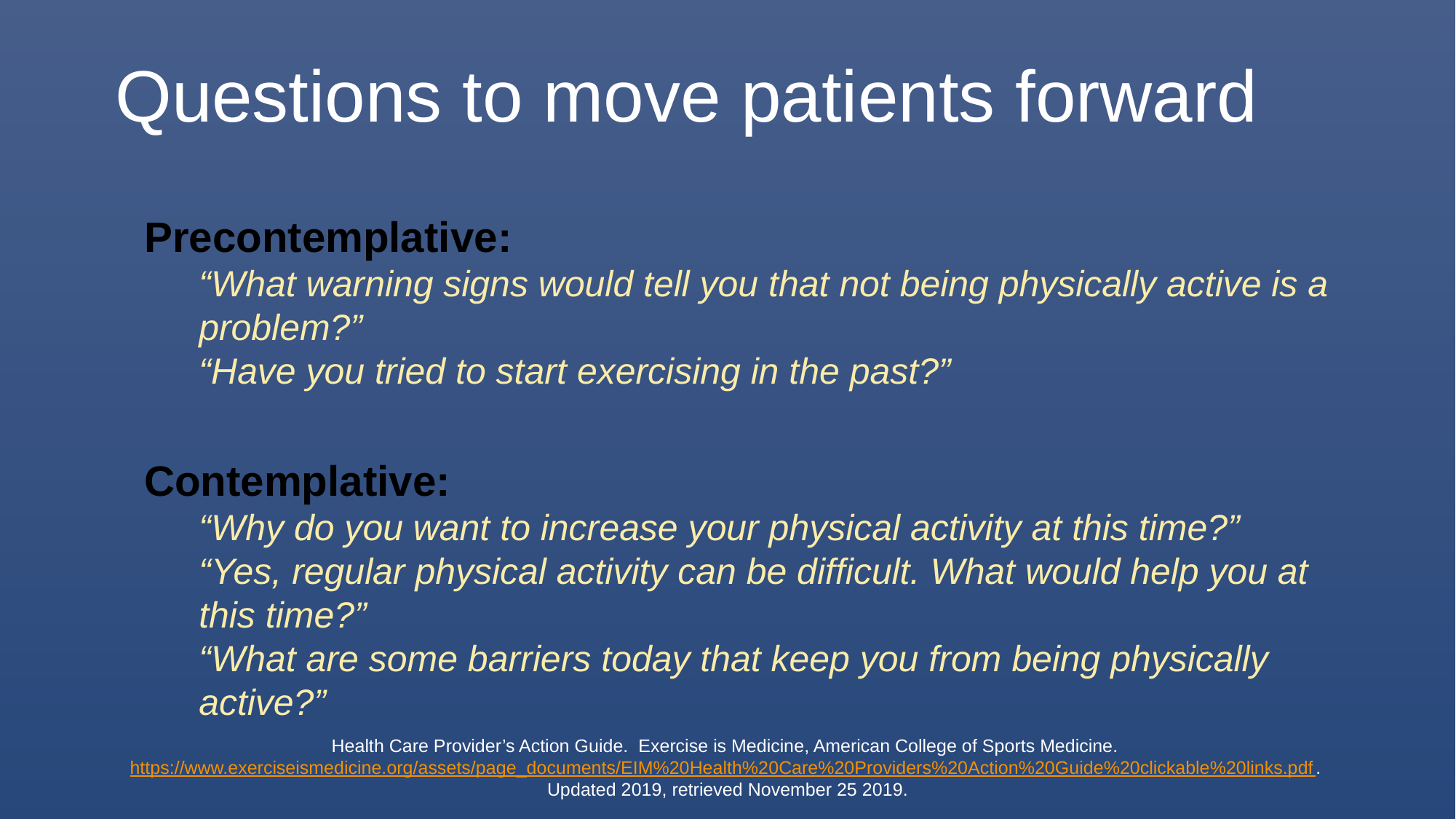

Questions to move patients forward
Precontemplative:
“What warning signs would tell you that not being physically active is a problem?”
“Have you tried to start exercising in the past?”
Contemplative:
“Why do you want to increase your physical activity at this time?”
“Yes, regular physical activity can be difficult. What would help you at this time?”
“What are some barriers today that keep you from being physically active?”
Health Care Provider’s Action Guide. Exercise is Medicine, American College of Sports Medicine. https://www.exerciseismedicine.org/assets/page_documents/EIM%20Health%20Care%20Providers%20Action%20Guide%20clickable%20links.pdf. Updated 2019, retrieved November 25 2019.

## Slide 27
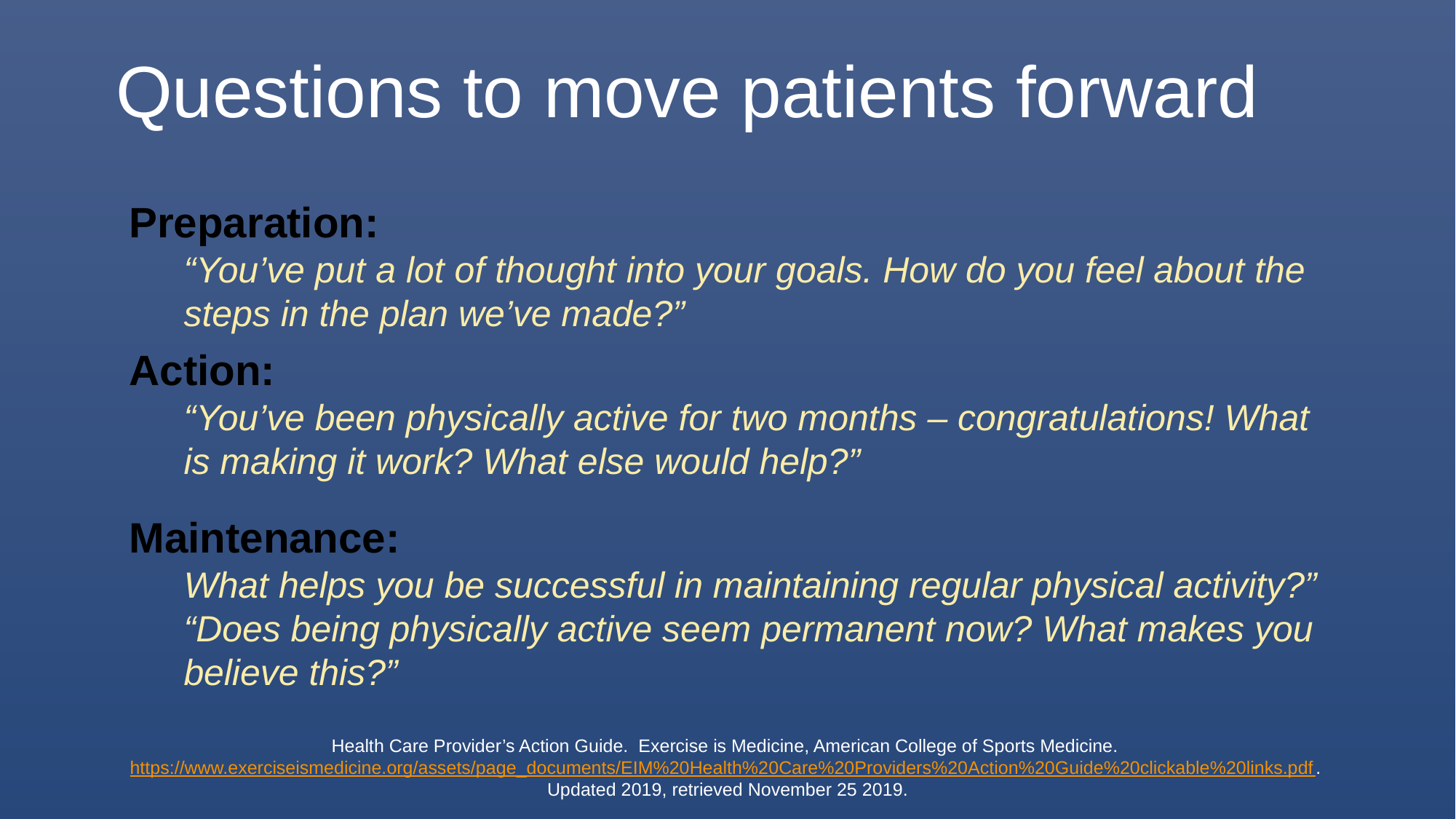

Questions to move patients forward
Preparation:
“You’ve put a lot of thought into your goals. How do you feel about the steps in the plan we’ve made?”
Action:
“You’ve been physically active for two months – congratulations! What is making it work? What else would help?”
Maintenance:
What helps you be successful in maintaining regular physical activity?”
“Does being physically active seem permanent now? What makes you believe this?”
Health Care Provider’s Action Guide. Exercise is Medicine, American College of Sports Medicine. https://www.exerciseismedicine.org/assets/page_documents/EIM%20Health%20Care%20Providers%20Action%20Guide%20clickable%20links.pdf. Updated 2019, retrieved November 25 2019.

## Slide 28
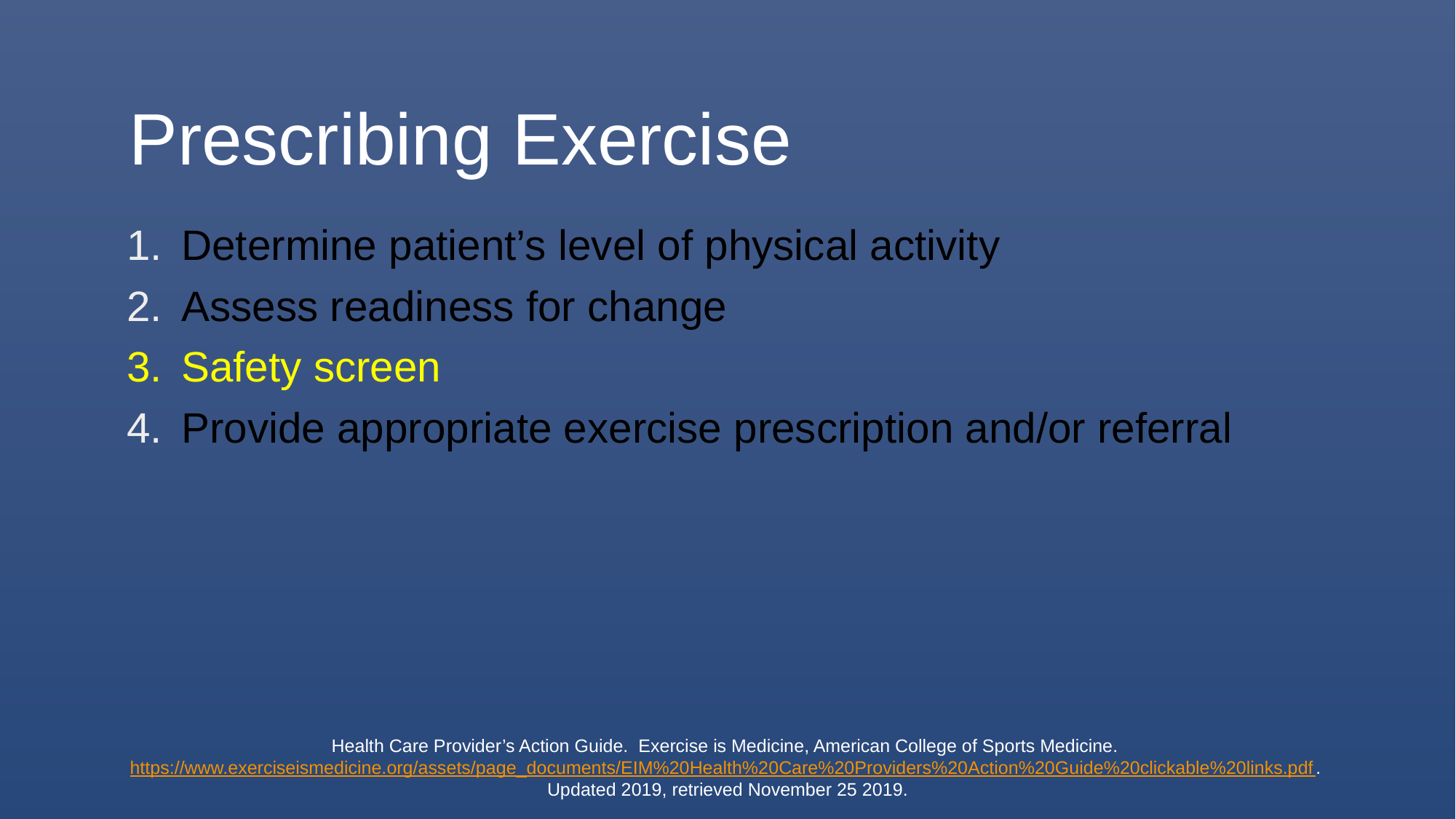

Prescribing Exercise
Determine patient’s level of physical activity
Assess readiness for change
Safety screen
Provide appropriate exercise prescription and/or referral
Health Care Provider’s Action Guide. Exercise is Medicine, American College of Sports Medicine. https://www.exerciseismedicine.org/assets/page_documents/EIM%20Health%20Care%20Providers%20Action%20Guide%20clickable%20links.pdf. Updated 2019, retrieved November 25 2019.

## Slide 29
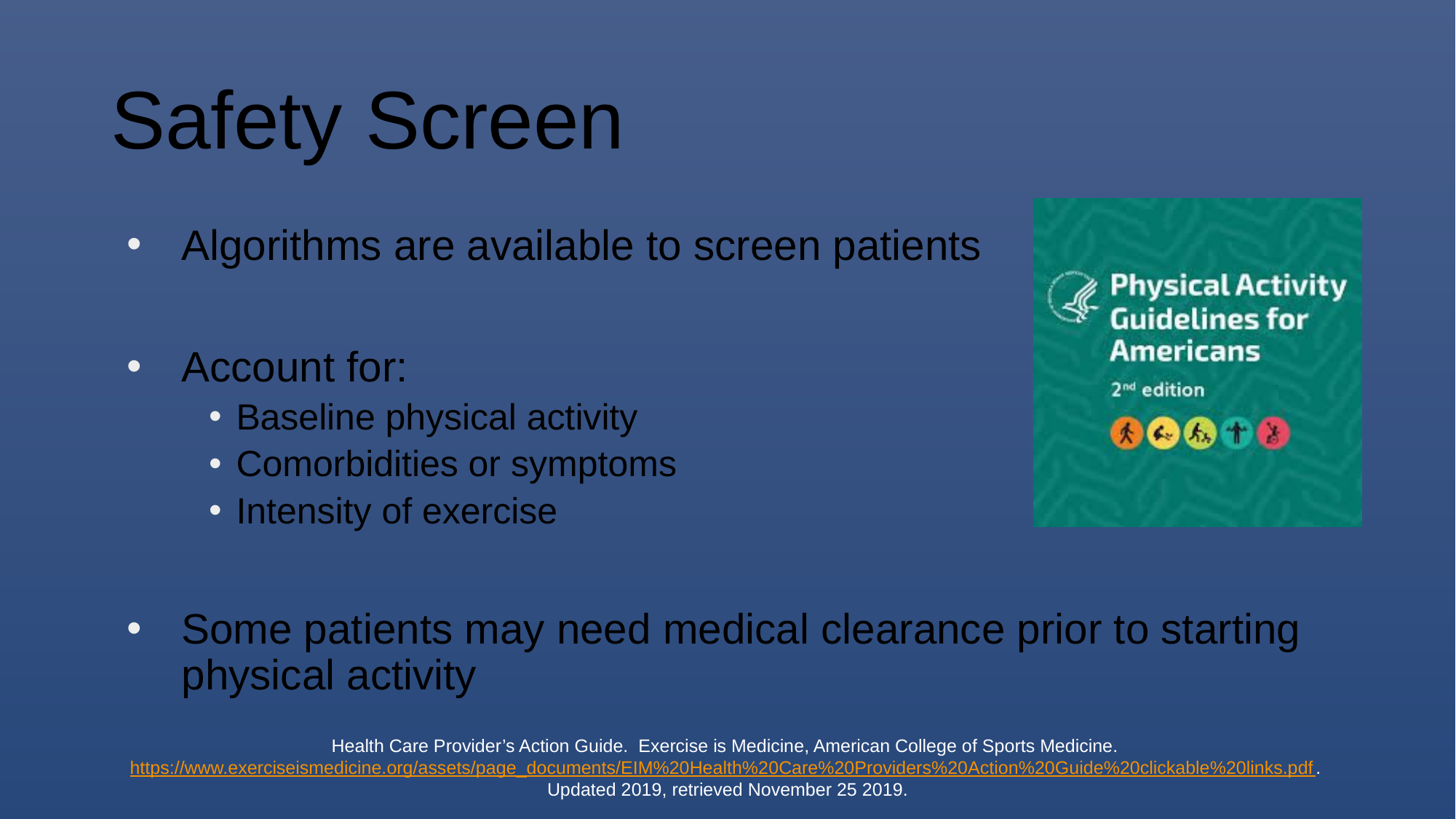

# Safety Screen
Algorithms are available to screen patients
Account for:
Baseline physical activity
Comorbidities or symptoms
Intensity of exercise
Some patients may need medical clearance prior to starting physical activity
Health Care Provider’s Action Guide. Exercise is Medicine, American College of Sports Medicine. https://www.exerciseismedicine.org/assets/page_documents/EIM%20Health%20Care%20Providers%20Action%20Guide%20clickable%20links.pdf. Updated 2019, retrieved November 25 2019.

## Slide 30
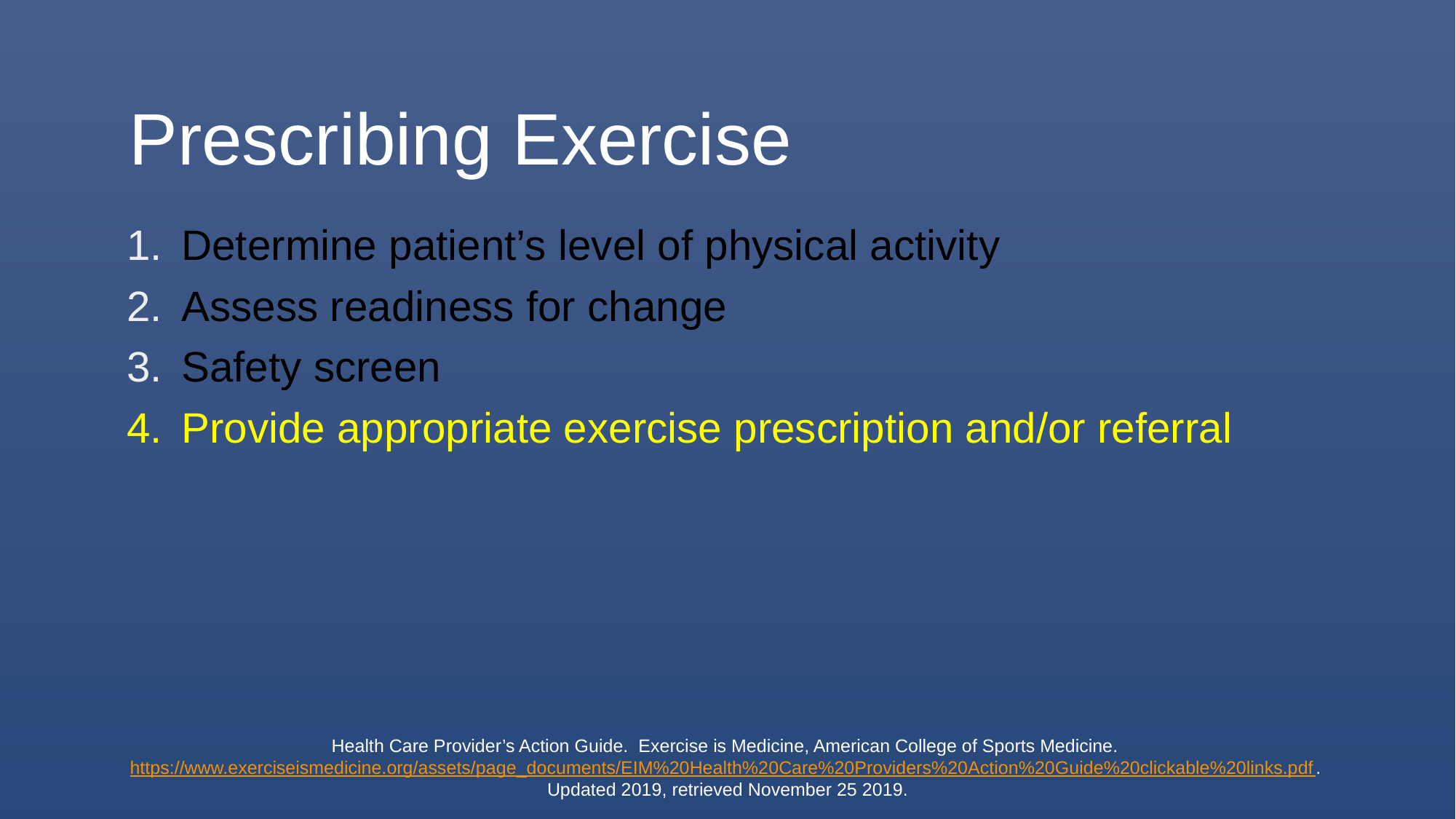

Prescribing Exercise
Determine patient’s level of physical activity
Assess readiness for change
Safety screen
Provide appropriate exercise prescription and/or referral
Health Care Provider’s Action Guide. Exercise is Medicine, American College of Sports Medicine. https://www.exerciseismedicine.org/assets/page_documents/EIM%20Health%20Care%20Providers%20Action%20Guide%20clickable%20links.pdf. Updated 2019, retrieved November 25 2019.

## Slide 31
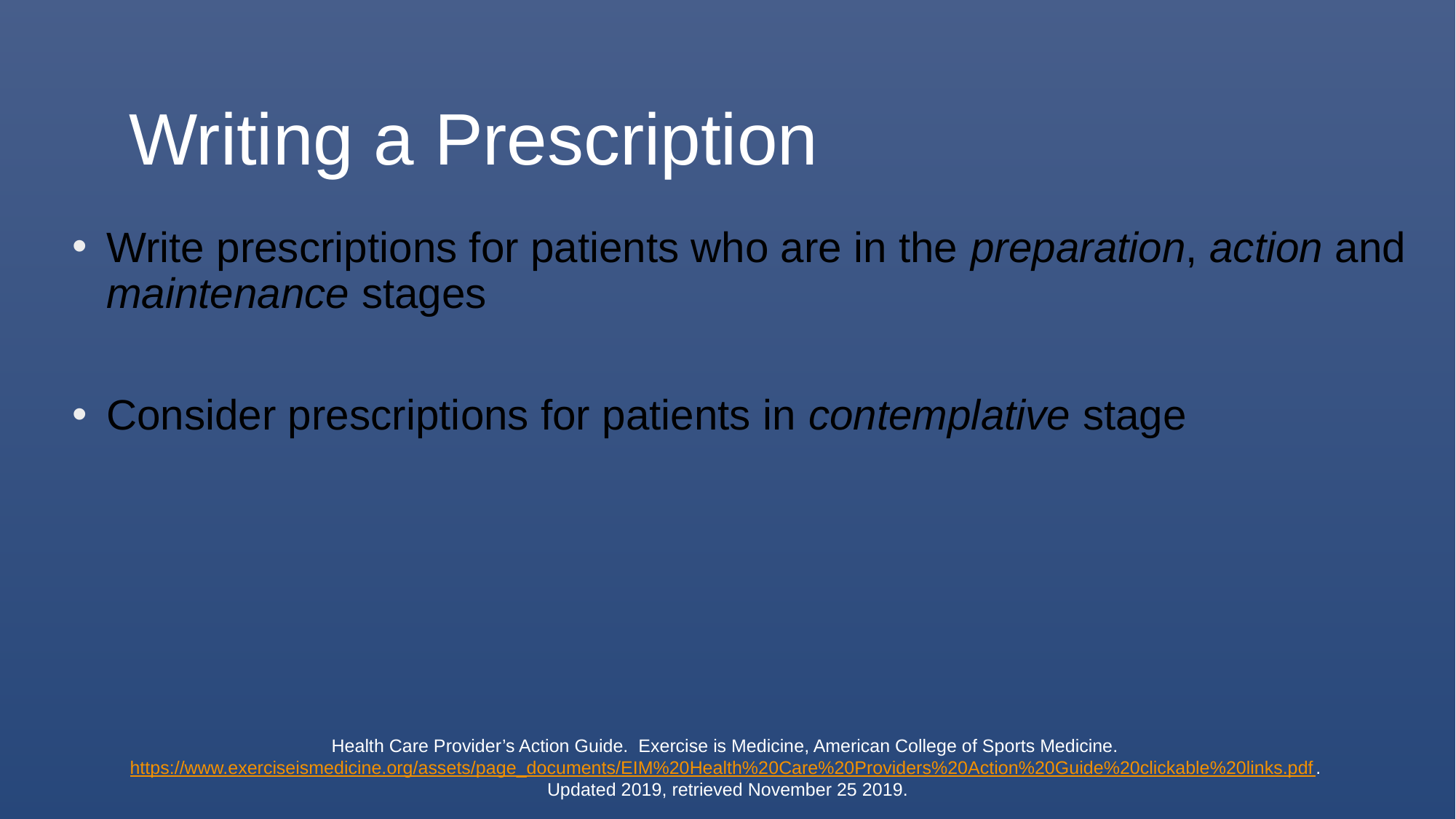

Writing a Prescription
Write prescriptions for patients who are in the preparation, action and maintenance stages
Consider prescriptions for patients in contemplative stage
Health Care Provider’s Action Guide. Exercise is Medicine, American College of Sports Medicine. https://www.exerciseismedicine.org/assets/page_documents/EIM%20Health%20Care%20Providers%20Action%20Guide%20clickable%20links.pdf. Updated 2019, retrieved November 25 2019.

## Slide 32
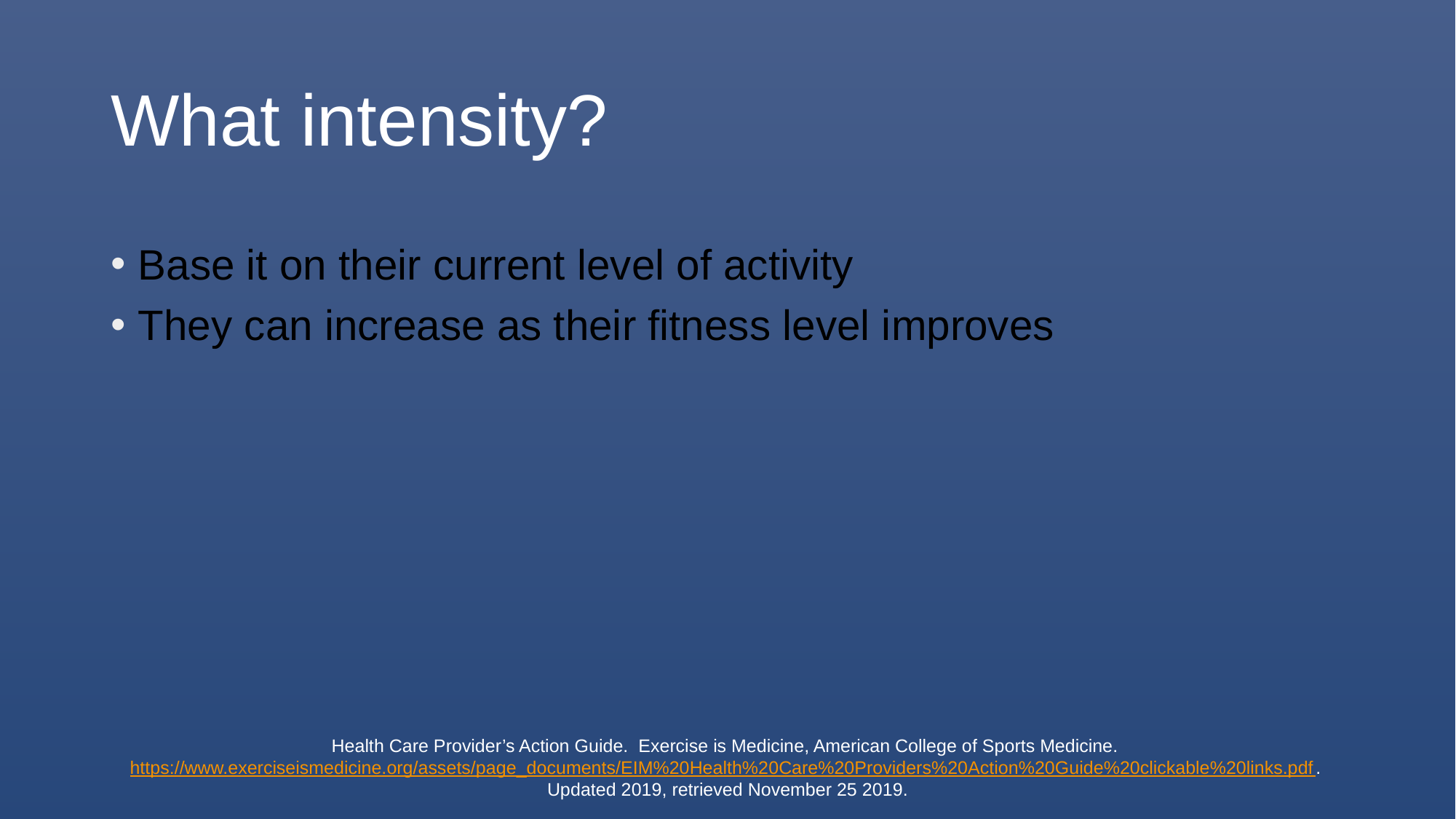

# What intensity?
Base it on their current level of activity
They can increase as their fitness level improves
Health Care Provider’s Action Guide. Exercise is Medicine, American College of Sports Medicine. https://www.exerciseismedicine.org/assets/page_documents/EIM%20Health%20Care%20Providers%20Action%20Guide%20clickable%20links.pdf. Updated 2019, retrieved November 25 2019.

## Slide 33
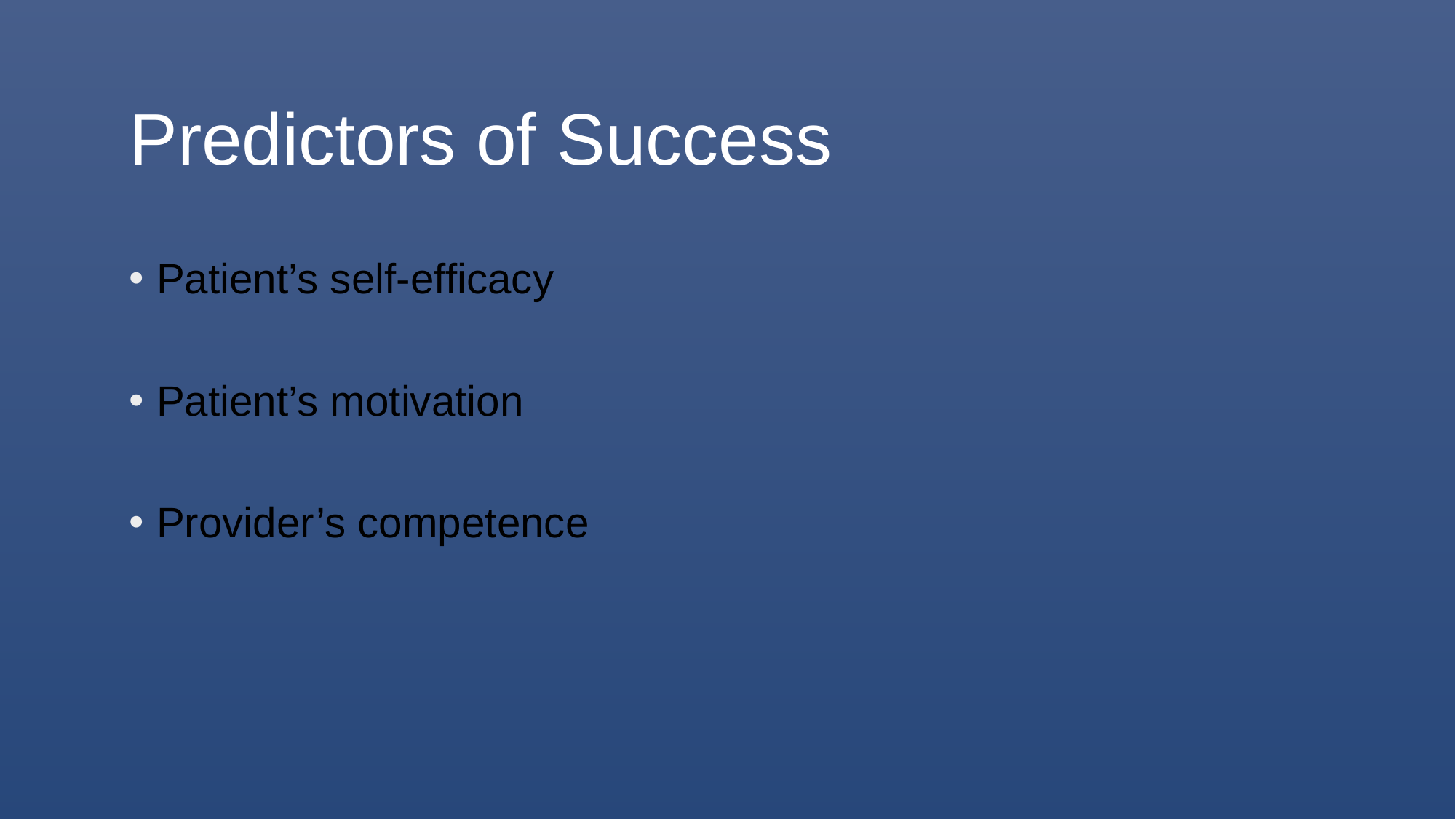

Predictors of Success
Patient’s self-efficacy
Patient’s motivation
Provider’s competence

## Slide 34
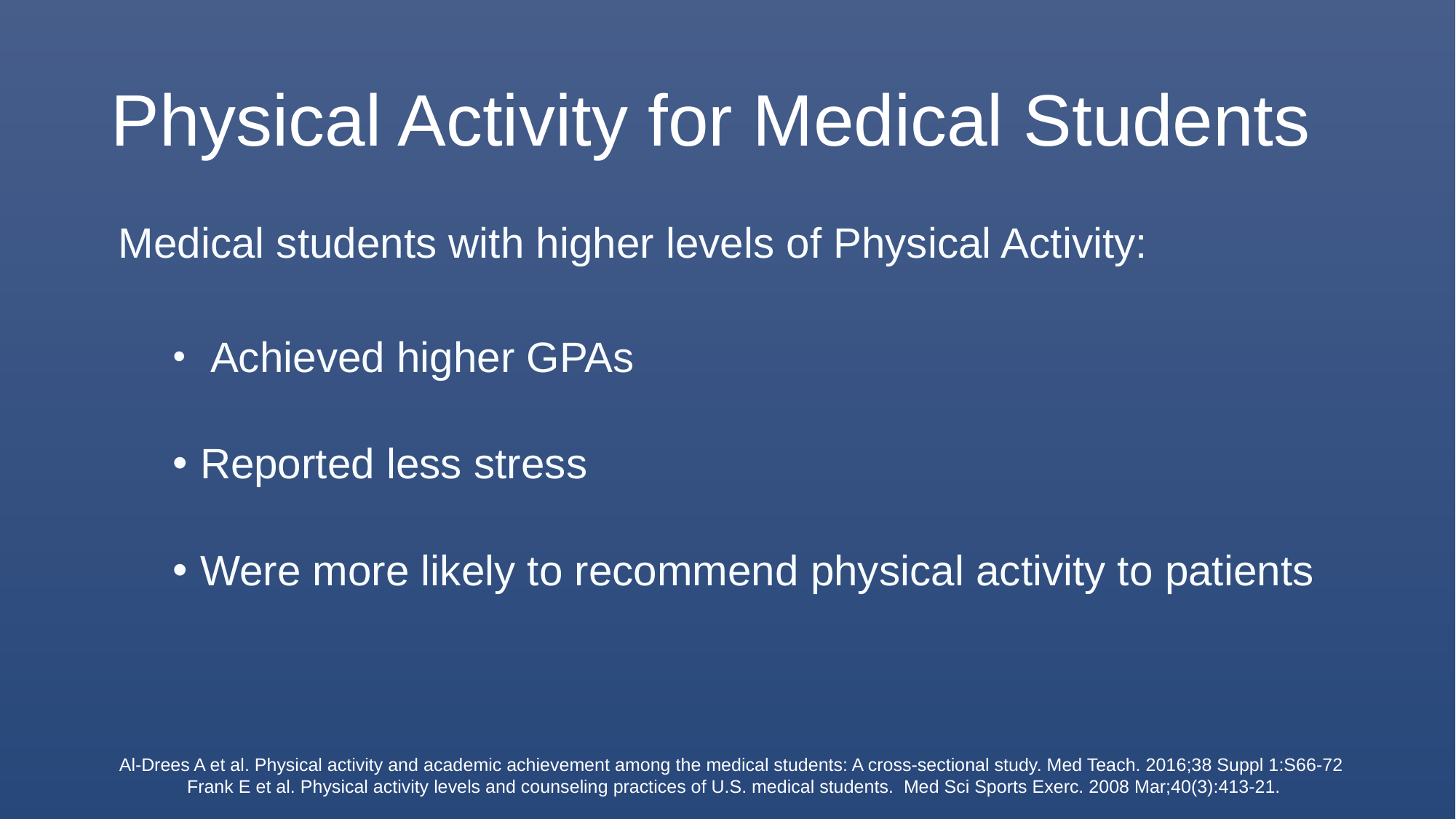

# Physical Activity for Medical Students
Medical students with higher levels of Physical Activity:
 Achieved higher GPAs
Reported less stress
Were more likely to recommend physical activity to patients
Al-Drees A et al. Physical activity and academic achievement among the medical students: A cross-sectional study. Med Teach. 2016;38 Suppl 1:S66-72
Frank E et al. Physical activity levels and counseling practices of U.S. medical students. Med Sci Sports Exerc. 2008 Mar;40(3):413-21.

## Slide 35
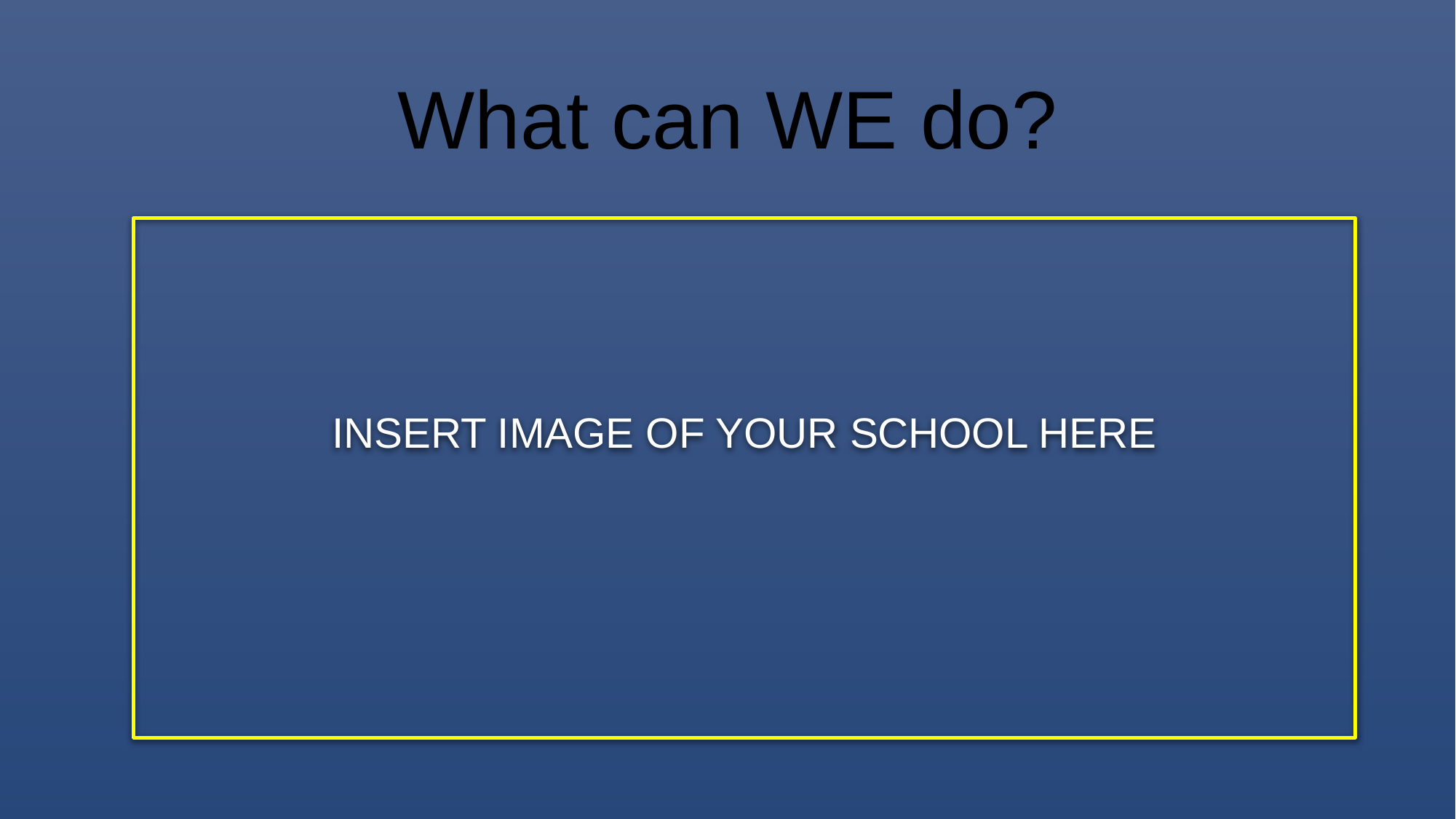

# What can WE do?
INSERT IMAGE OF YOUR SCHOOL HERE
