# Supplementary material for: An Interactive Lifestyle Medicine Curriculum for Third-Year Medical Students to Promote Student and Patient Wellness
Source: MedEdPORTAL. 2020 Sep 18;16:10972. doi: 10.15766/mep_2374-8265.10972 (PMC7499809; doi:10.15766/mep_2374-8265.10972)
Supplement: Supplementary file 1 — Introduction & Stress Management Presentation.pptxIntroduction & Stress Management Facilitator Guide.docxUnhealthy Thoughts Handout.pdfGood Things Worksheet.pdfNutrition Presentation.pptxNutrition Facilitator Guide.docxPhysical Activity Presentation.pptxPhysical Activity Facilitator Guide.docxPresession Evaluation.docxPostsession Evaluation.docxSession Evaluation.docx [file mep_2374-8265.10972-s001.zip › H. Physical Activity Facilitator Guide.docx]

**Facilitator Guide**

**Lifestyle Medicine & Student Wellness: Physical Activity**

Materials needed:

- AV Equipment for powerpoint presentation
- Space to walk around after class (ask students to wear comfortable clothes in advance)

Slides 1-2:  Introduction/Objectives (5 minutes)

Slides 3-7: Benefits of Physical Activity on Health (5 minutes)

Slides 8-11: Physical Activity Guidelines for Specific Populations (4 minutes)

Slides 12-16: National data about who’s meeting the guidelines (1 minute)

Slides 17-35: Physician’s Role and Prescribing Exercise (15 minutes)

Slides 34-35: Physical Activity for Medical Students (5 minutes)

Solicit students’ ideas about how to increase physical activity opportunities on campus; we have submitted them to the relevant stakeholders in the past. After the talk, we take the students for a 10-15 minute walk around campus.
