# Supplementary material for: An Interactive Lifestyle Medicine Curriculum for Third-Year Medical Students to Promote Student and Patient Wellness
Source: MedEdPORTAL. 2020 Sep 18;16:10972. doi: 10.15766/mep_2374-8265.10972 (PMC7499809; doi:10.15766/mep_2374-8265.10972)
Supplement: Supplementary file 1 — Introduction & Stress Management Presentation.pptxIntroduction & Stress Management Facilitator Guide.docxUnhealthy Thoughts Handout.pdfGood Things Worksheet.pdfNutrition Presentation.pptxNutrition Facilitator Guide.docxPhysical Activity Presentation.pptxPhysical Activity Facilitator Guide.docxPresession Evaluation.docxPostsession Evaluation.docxSession Evaluation.docx [file mep_2374-8265.10972-s001.zip › I. Presession Evaluation.docx]

**Lifestyle Medicine & Student Wellness Curriculum Pre-Evaluation**

| **Statement** | **Strongly Agree** | **Agree** | **Neutral** | **Disagree** | **Strongly Disagree** |
| --- | --- | --- | --- | --- | --- |
| **As a medical student, I feel that lifestyle factors are an important contributor to my patients’ health and wellness.** |  |  |  |  |  |
| **As a medical student, I feel that lifestyle factors are an important contributor to my own health and wellness.** |  |  |  |  |  |
| **I understand the relationship between lifestyle factors and the health and wellness of my patients.** |  |  |  |  |  |
| **I understand the relationship between lifestyle factors and my own health and wellness.** |  |  |  |  |  |
| **I am confident in my ability to counsel patients about positive behavioral changes.** |  |  |  |  |  |
| **I am confident in my ability to make my own positive behavioral changes.** |  |  |  |  |  |

**Additional Comments:**
