# Supplementary material for: An Interactive Lifestyle Medicine Curriculum for Third-Year Medical Students to Promote Student and Patient Wellness
Source: MedEdPORTAL. 2020 Sep 18;16:10972. doi: 10.15766/mep_2374-8265.10972 (PMC7499809; doi:10.15766/mep_2374-8265.10972)
Supplement: Supplementary file 1 — Introduction & Stress Management Presentation.pptxIntroduction & Stress Management Facilitator Guide.docxUnhealthy Thoughts Handout.pdfGood Things Worksheet.pdfNutrition Presentation.pptxNutrition Facilitator Guide.docxPhysical Activity Presentation.pptxPhysical Activity Facilitator Guide.docxPresession Evaluation.docxPostsession Evaluation.docxSession Evaluation.docx [file mep_2374-8265.10972-s001.zip › K. Session Evaluation.docx]

**Lifestyle Medicine & Medical Student Wellness**

**1. Overall rating of session:**

5-Excellent 4-Very Good 3-Good 2-Fair 1-Poor

**2. What did you like best about this session?**

**3. What would you change about this session?**

**Additional comments:**

**Thank you!**
